# Supplementary material for: Synthesis and Density Functional Theory Studies of Azirinyl and Oxiranyl Functionalized Isoindigo and (3Z,3’Z)-3,3’-(ethane-1,2-diylidene)bis(indolin-2-one) Derivatives
Source: Molecules. 2019 Oct 10;24(20):3649. doi: 10.3390/molecules24203649 (PMC6832541; doi:10.3390/molecules24203649)
Supplement: Supplementary file 1 [file molecules-24-03649-s001.pdf]

Synthesis and density functional theory studies of azirinyl and oxiranyl functionalized isoindigo and (3Z,3'Z)-3,3'-(ethane-1,2-diylidene)bis(indolin-2-one) derivatives

Gholamhossein Khalili *[1,2], Patrick M. McCosker [1,3], Timothy Clark [3], Paul A. Keller *[1]

[1] School of Chemistry and Molecular Biosciences, Molecular Horizons, Illawarra Health and Medical Research Institute University of Wollongong, Wollongong, New South Wales, 2522, Australia; keller@uow.edu.au

[2] Chemistry Department, Bushehr Branch, Islamic Azad University, PO Box 7519619555, Bushehr, Iran; khalili_gh1352@yahoo.com

[3] Department of Chemistry and Pharmacy, Computer-Chemistry-Center (CCC), Friedrich-Alexander-Universität Erlangen-Nürnberg (FAU), Nägelsbachstrasse 25, 91052 Erlangen, Germany

* Correspondence: khalili_gh1352@yahoo.com; Tel.: +987715682305

Supporting Information

Table of Contents

^{1}H- and ^{13}C-NMR spectra for compounds **3**……………...SI2

^{1}H- and ^{13}C-NMR spectra for compounds **5**…………..………..SI3

^{1}H, ^{13}C, and HSQC-NMR spectra for compounds **7a-7i**…...SI4

^{1}H- and ^{13}C-NMR spectra for compounds **6d-6i**……..SI23

TD-DFT Absorption Spectra Modelling………………………………………………………….........SI29

DFT Calculated HOMO and LUMO Energies………………………………………………….........SI31

DFT HOMO and LUMO for Selected Dimers Modelled.……………………………………...…........SI32

DFT Optimised XYZ coordinates..……………………………………………………………...........SI33

Figure S1. ^{1}H and ^{13}C NMR spectra of **3**

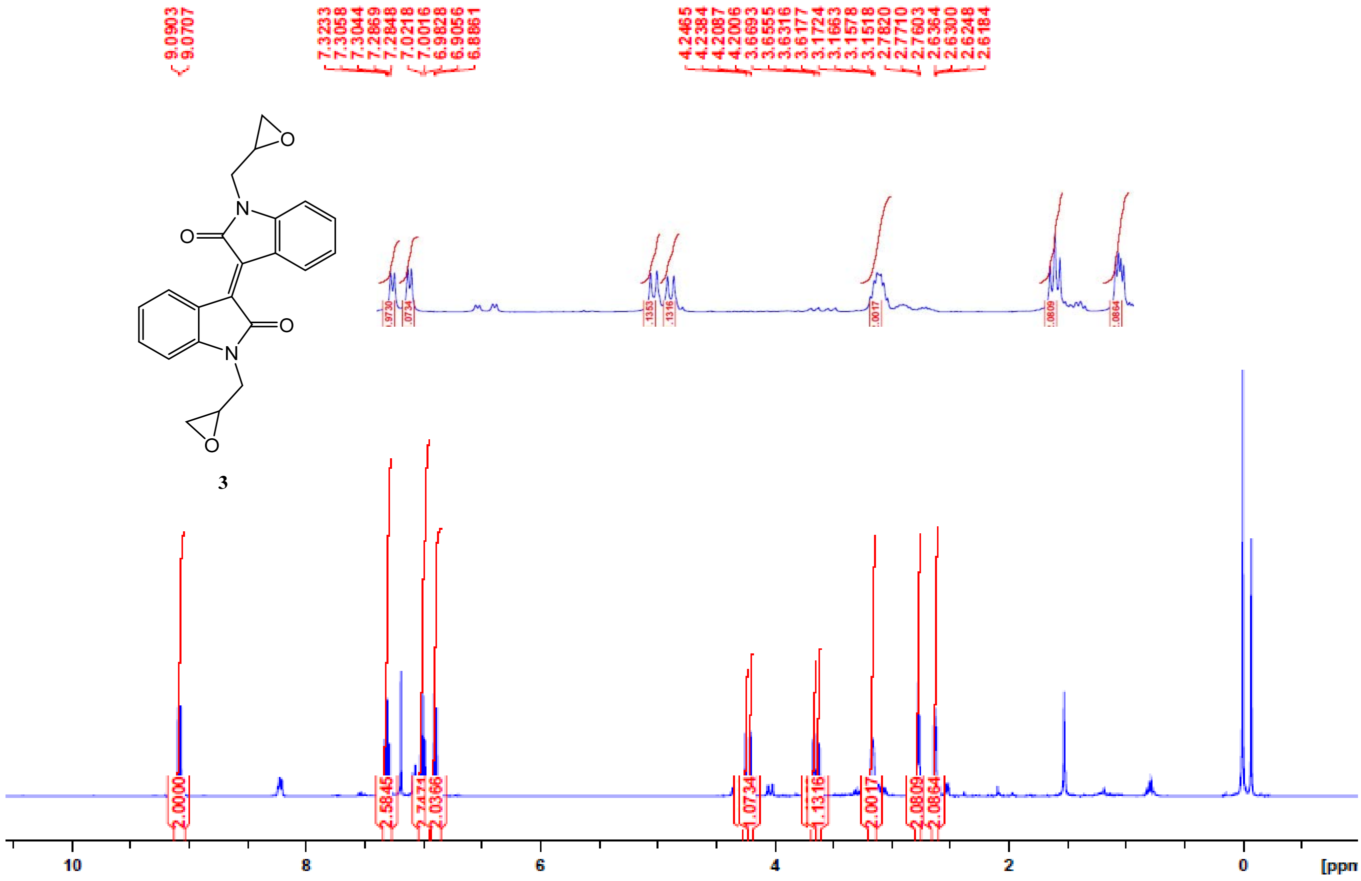

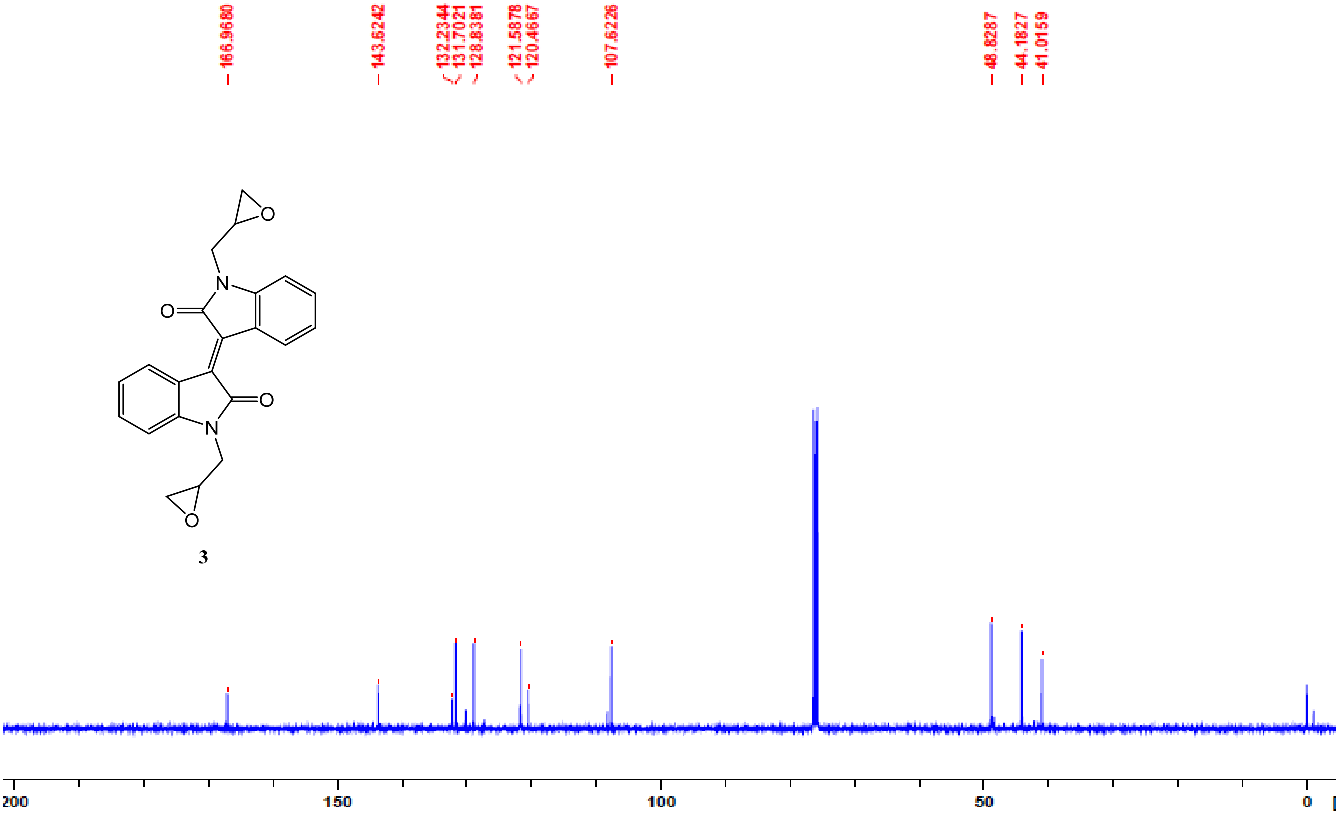

Figure S2. ^{1}H and ^{13}C NMR spectra of **5**

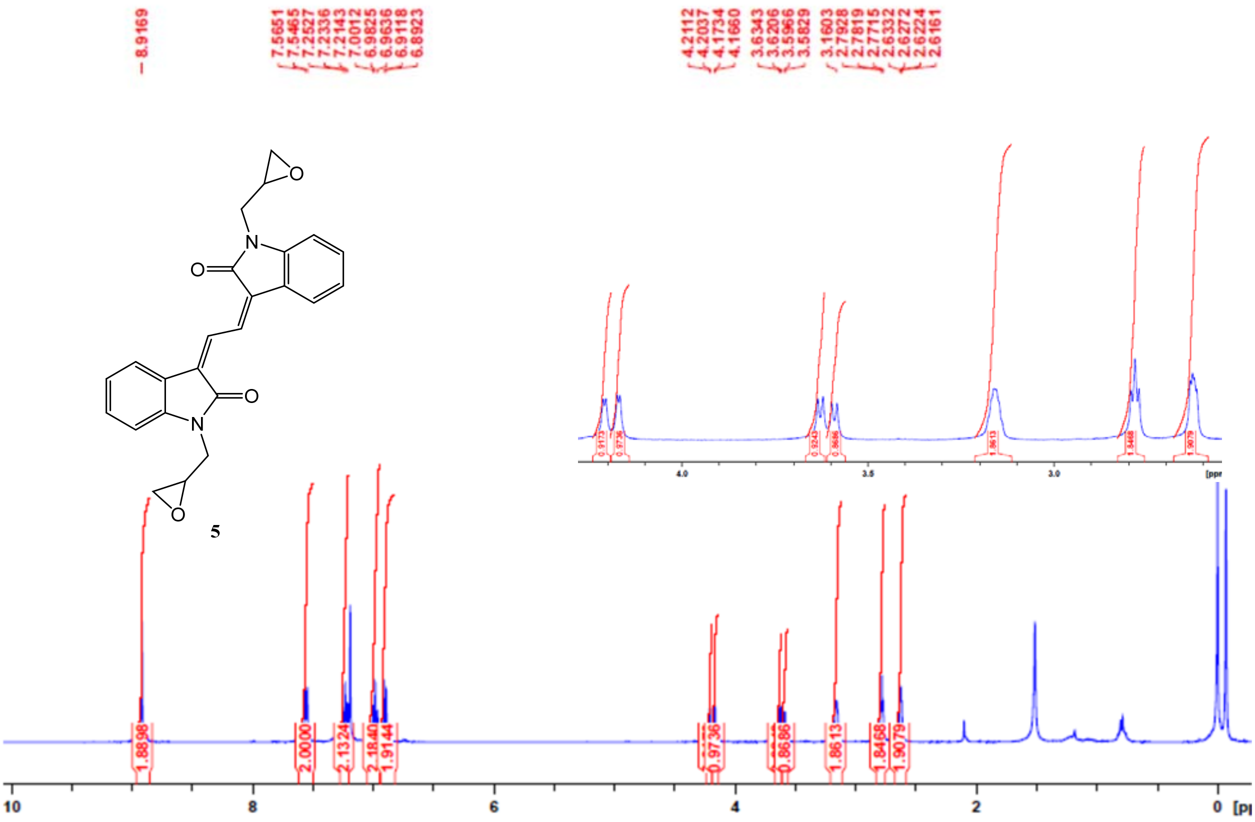

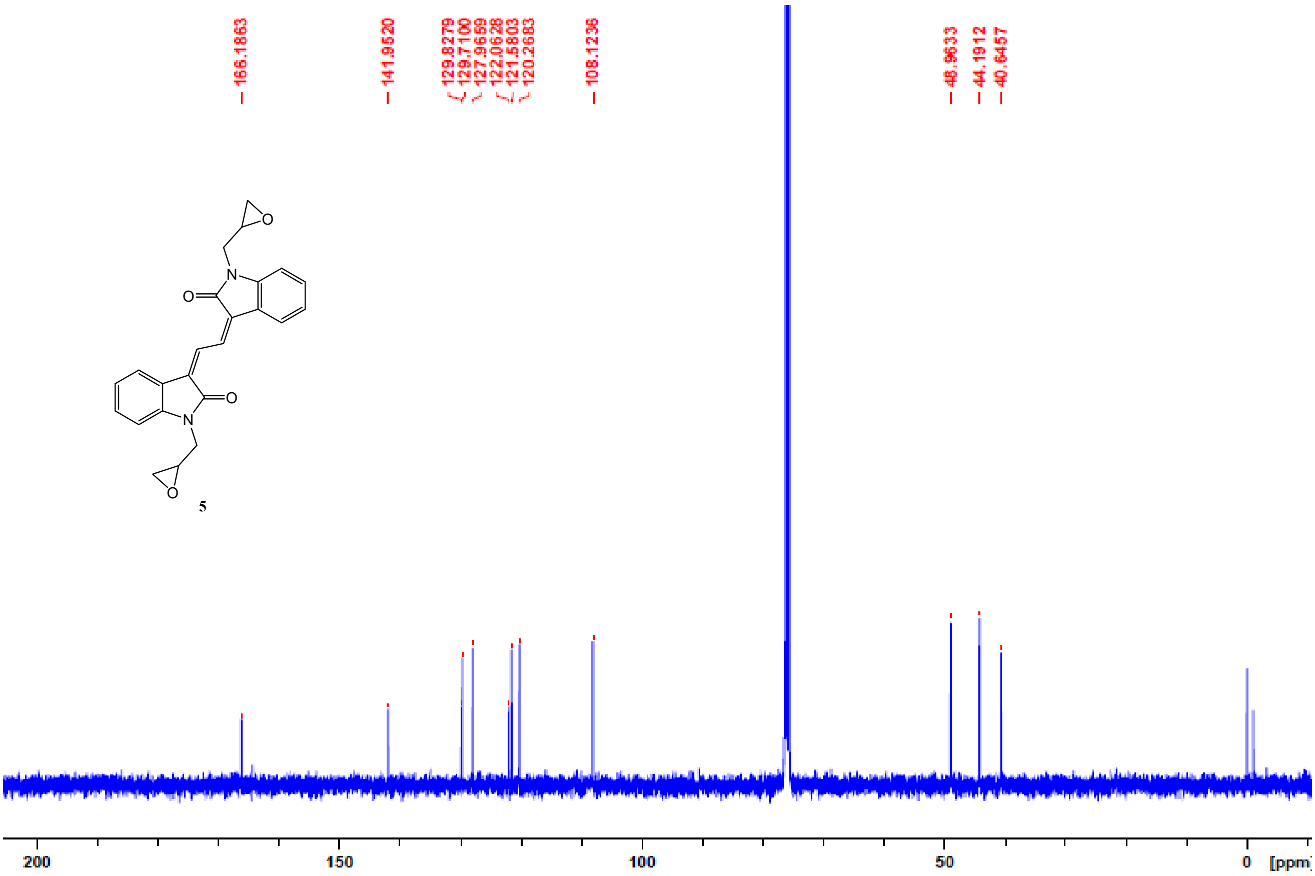

Figure S3. ^{1}H and ^{13}C NMR spectra of **7a**

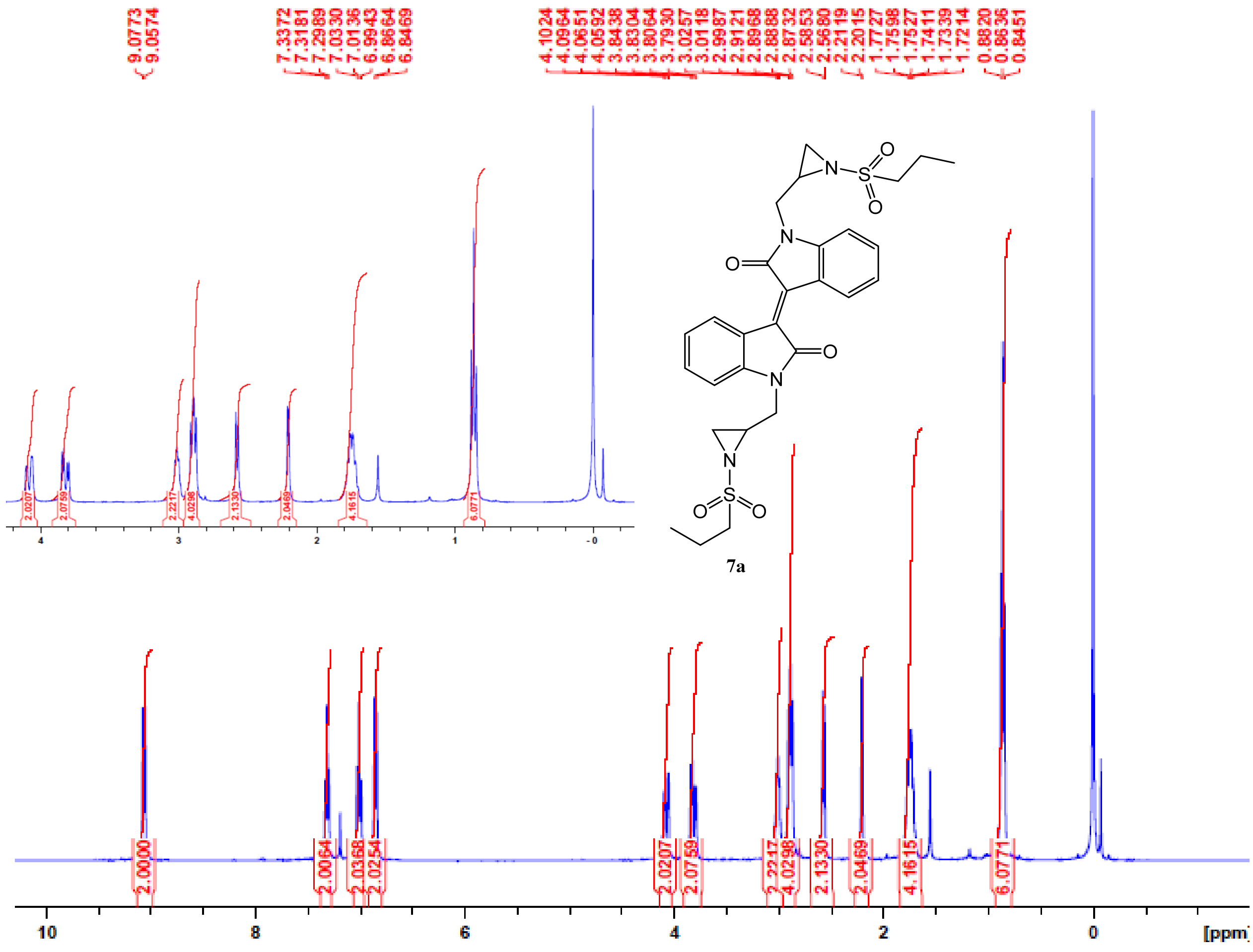

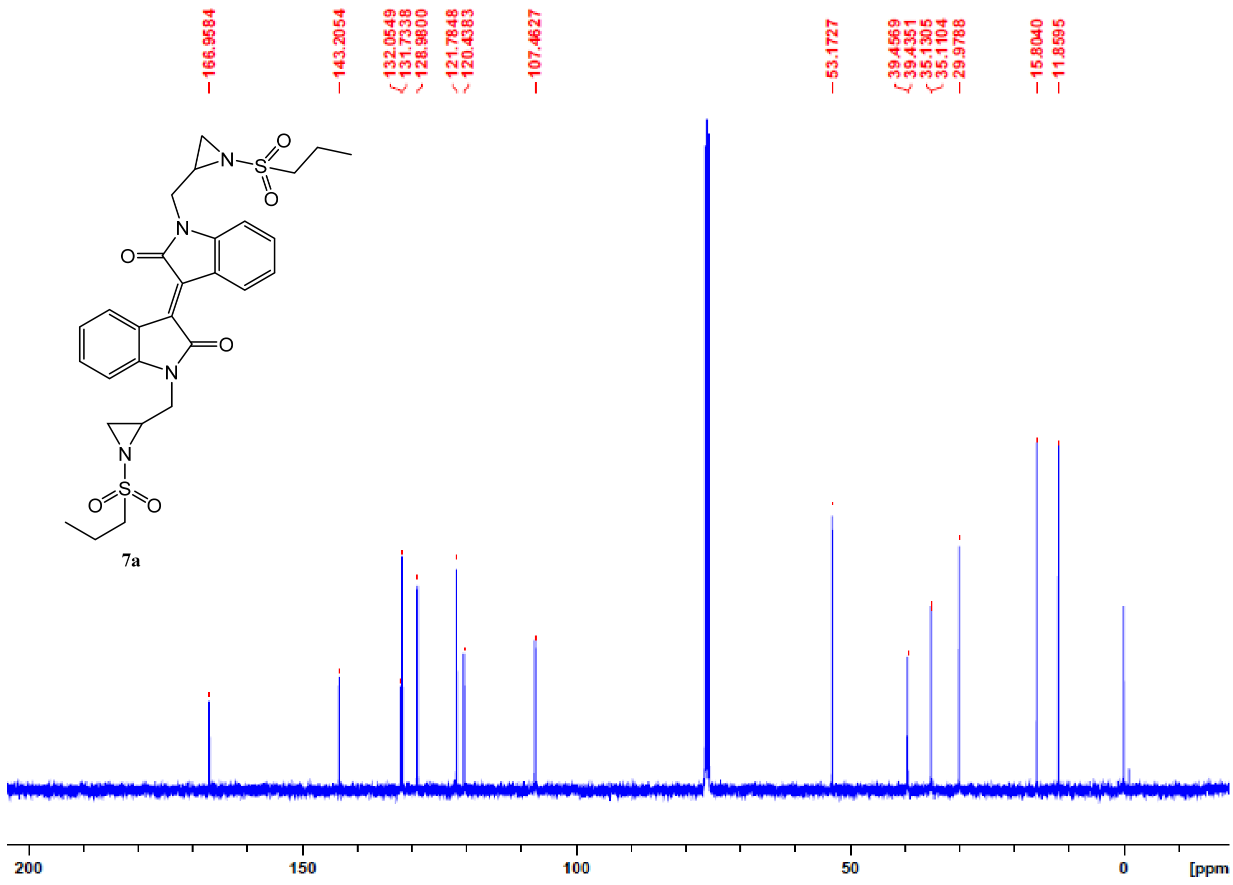

Figure S4. HSQC-NMR spectrum of **7a**

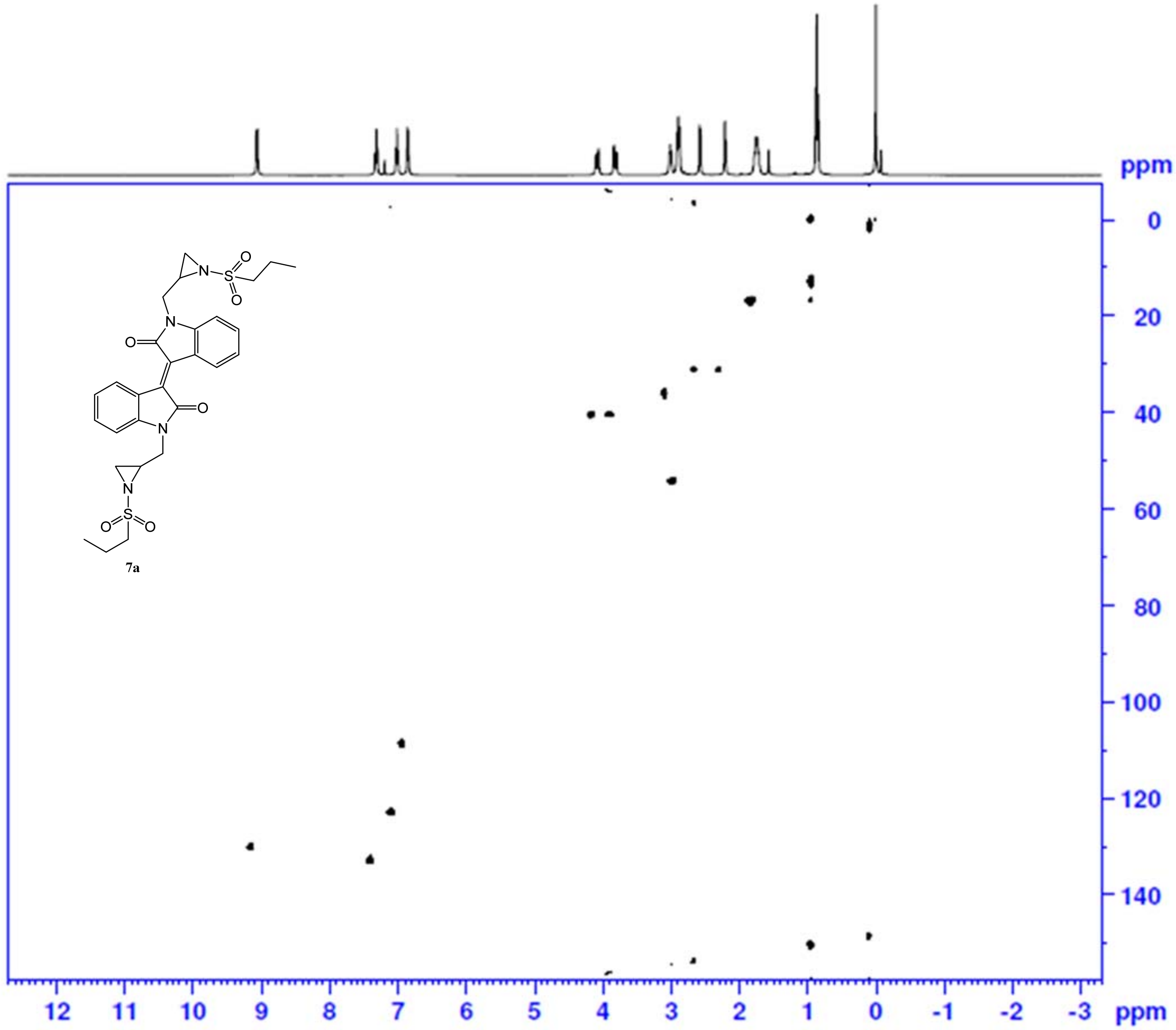

Figure S5. ^{1}H and ^{13}C NMR spectra of **7b**

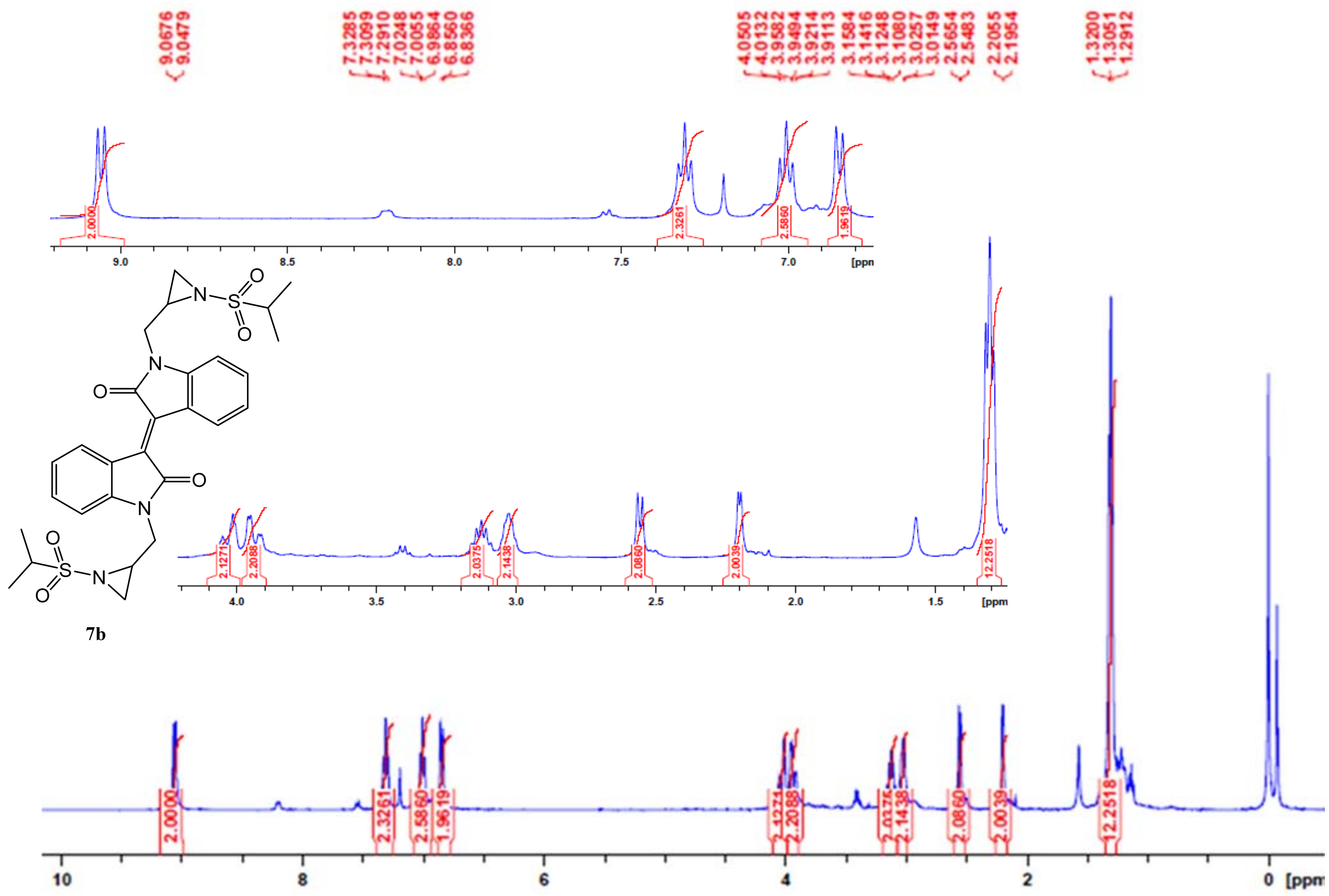

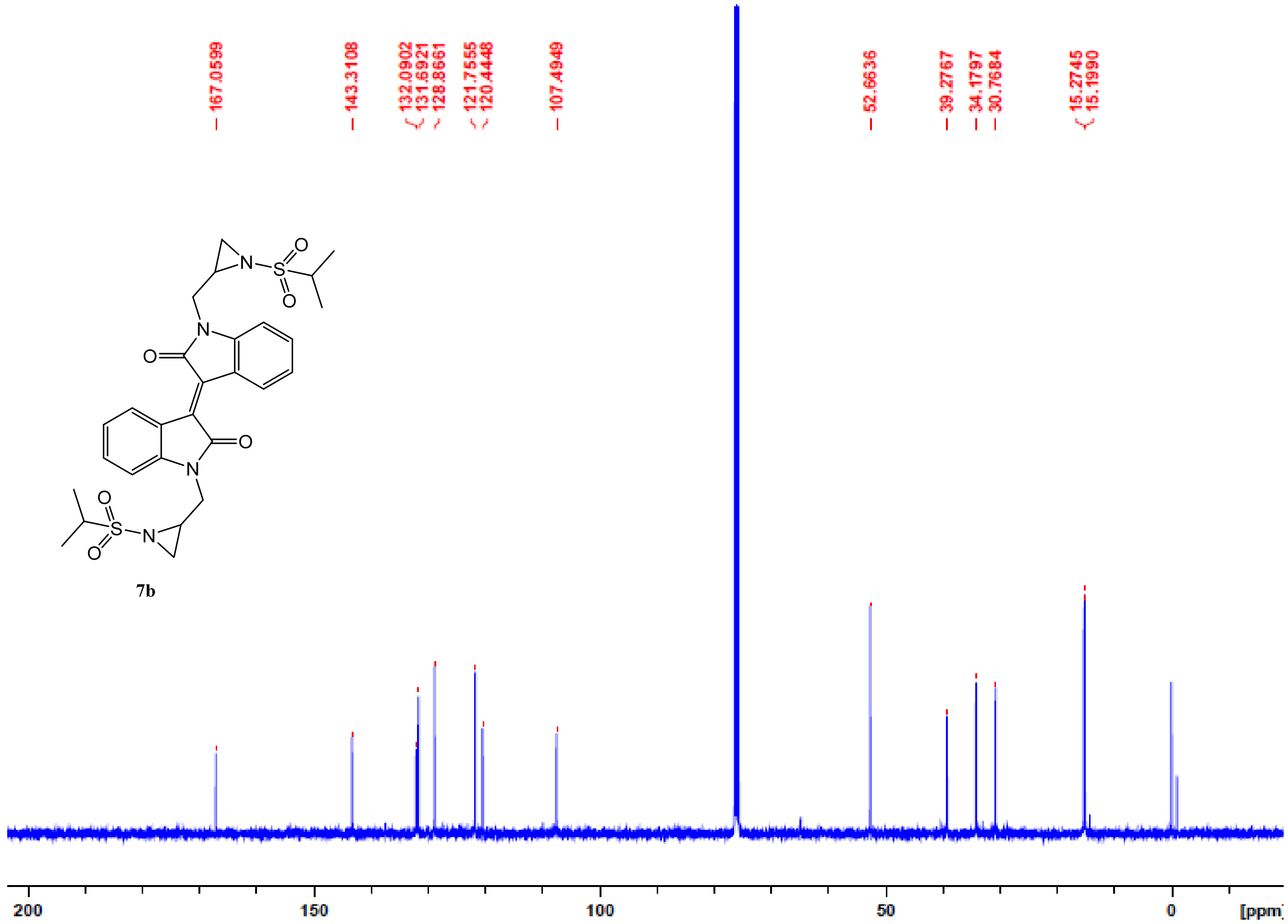

Figure S6. HSQC-NMR spectrum of **7b**

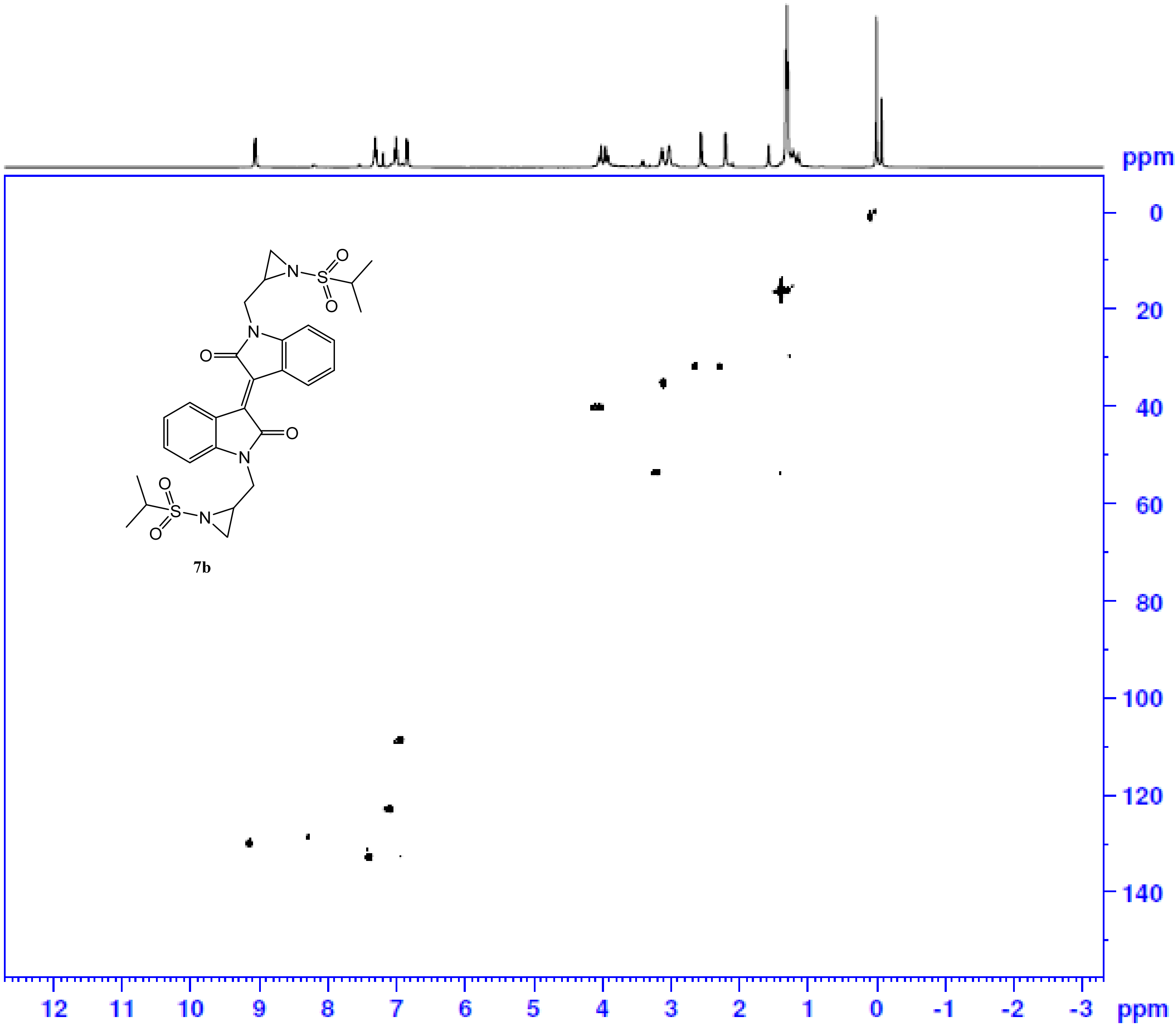

Figure S7. ^{1}H and ^{13}C NMR spectra of **7c**

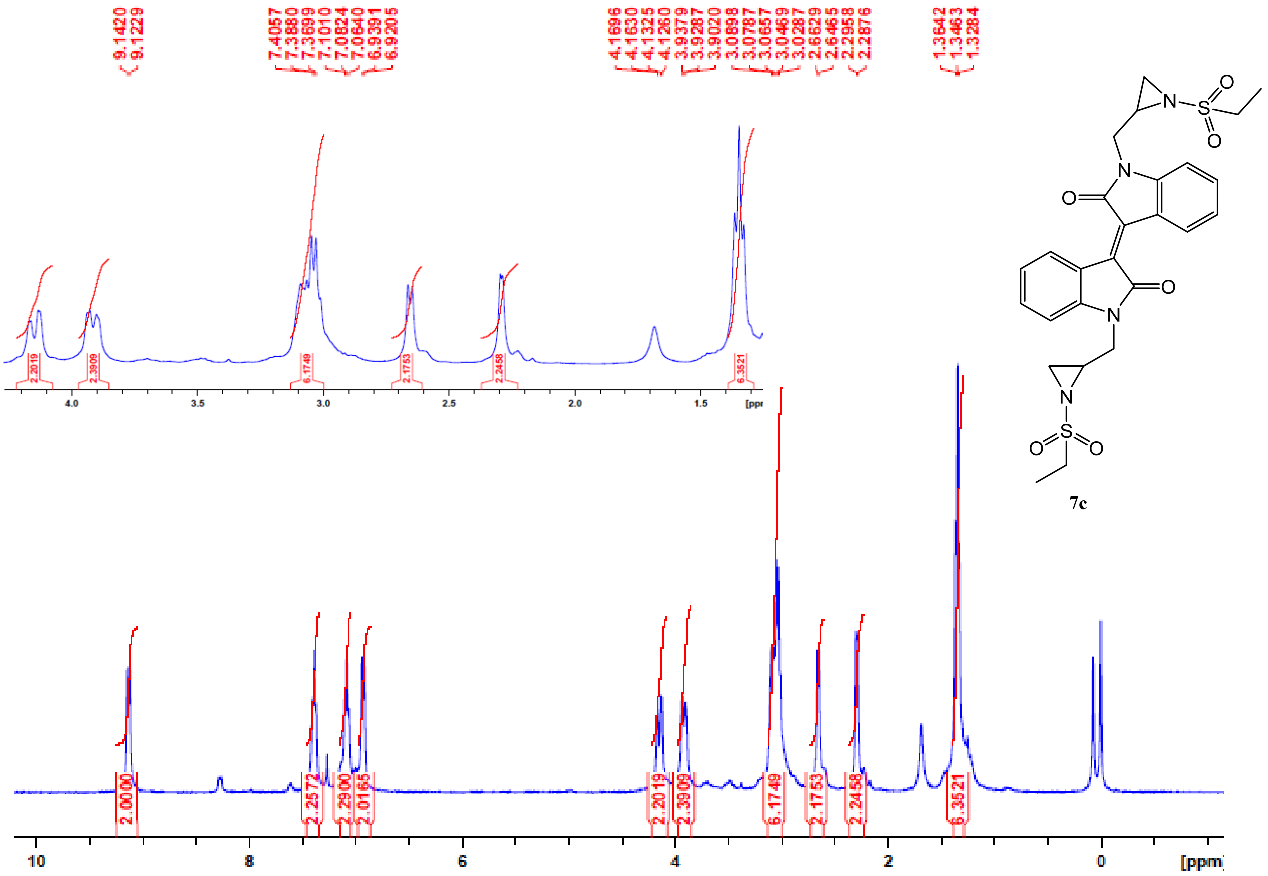

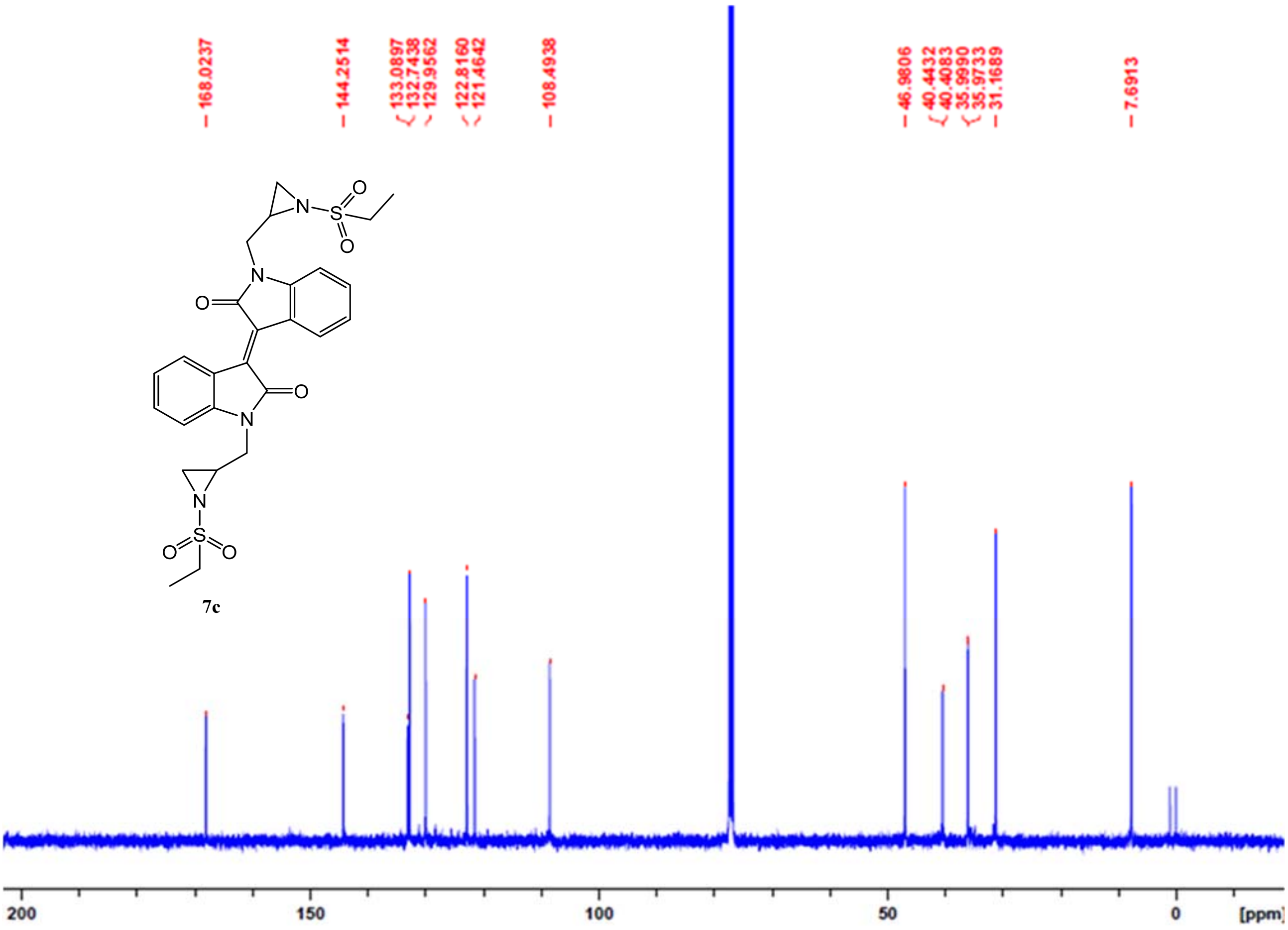

Figure S8. HSQC-NMR spectrum of **7c**

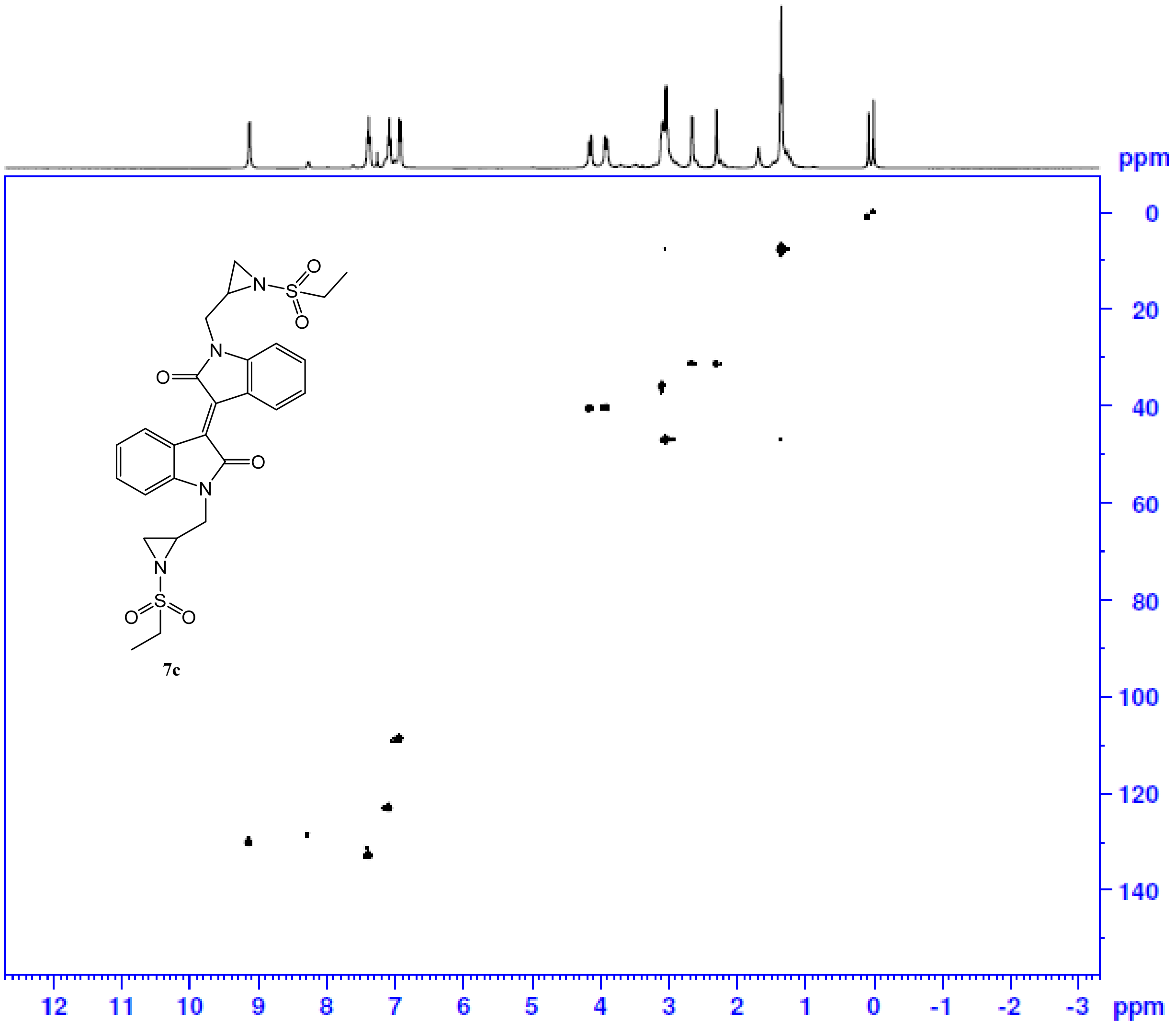

Figure S9. ^{1}H and ^{13}C NMR spectra of **7d**

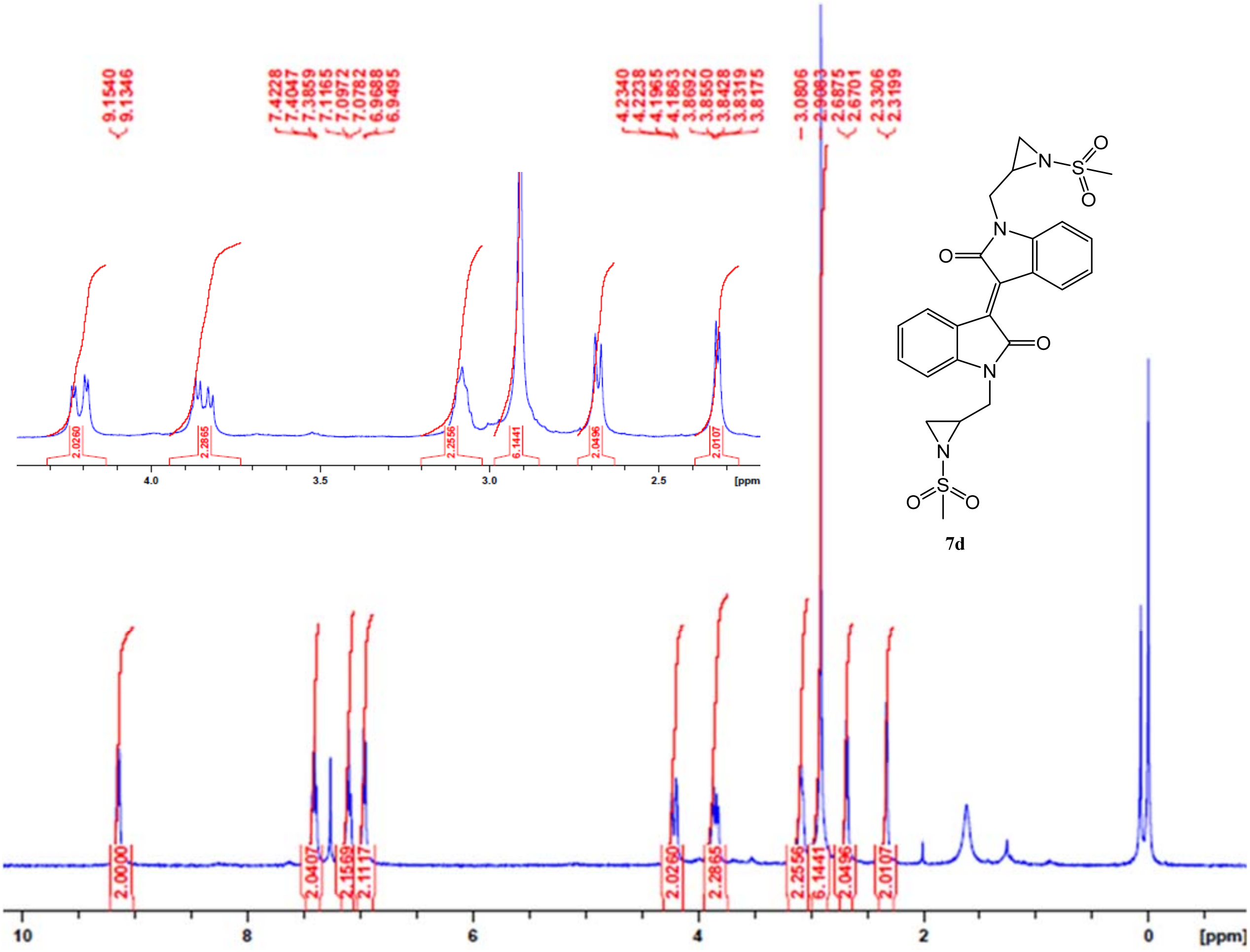

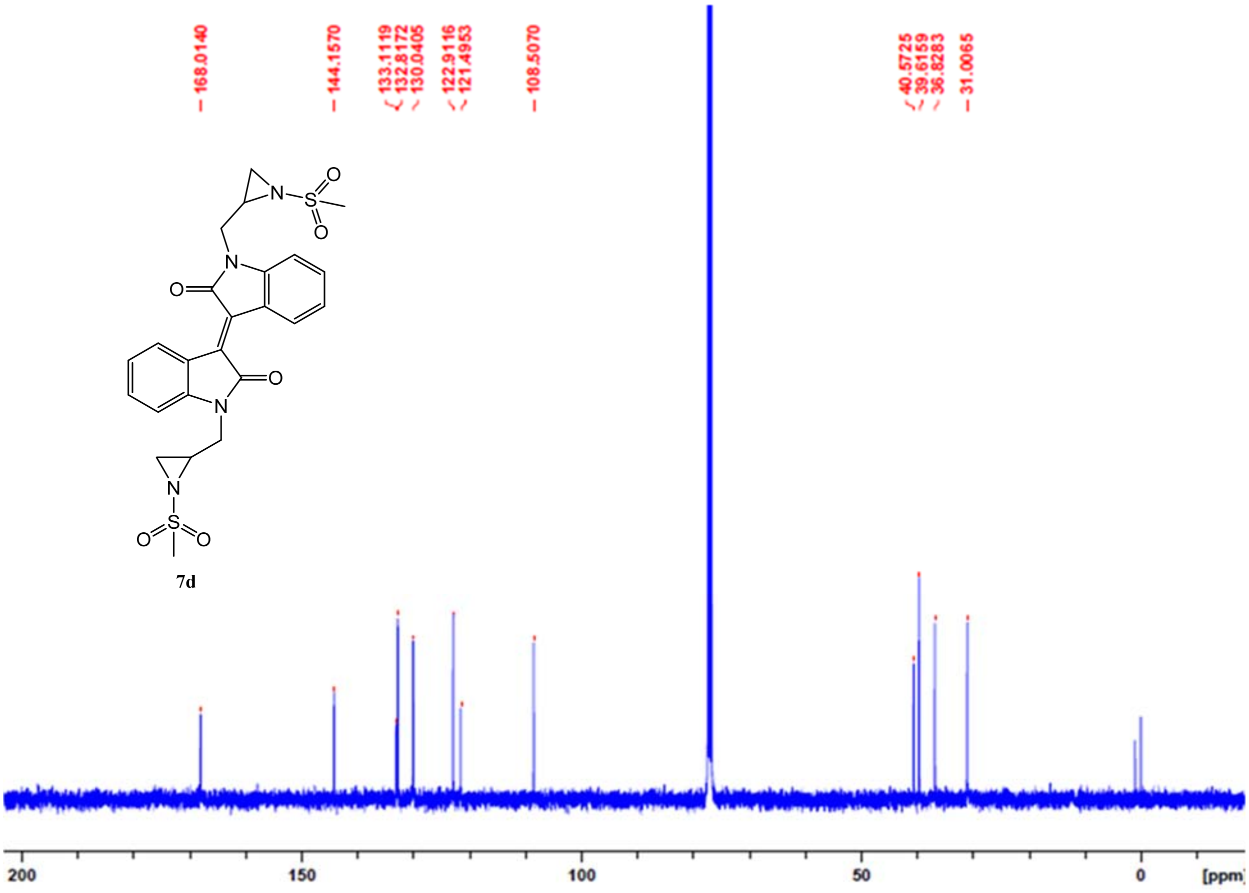

Figure S10. HSQC-NMR spectrum of **7d**

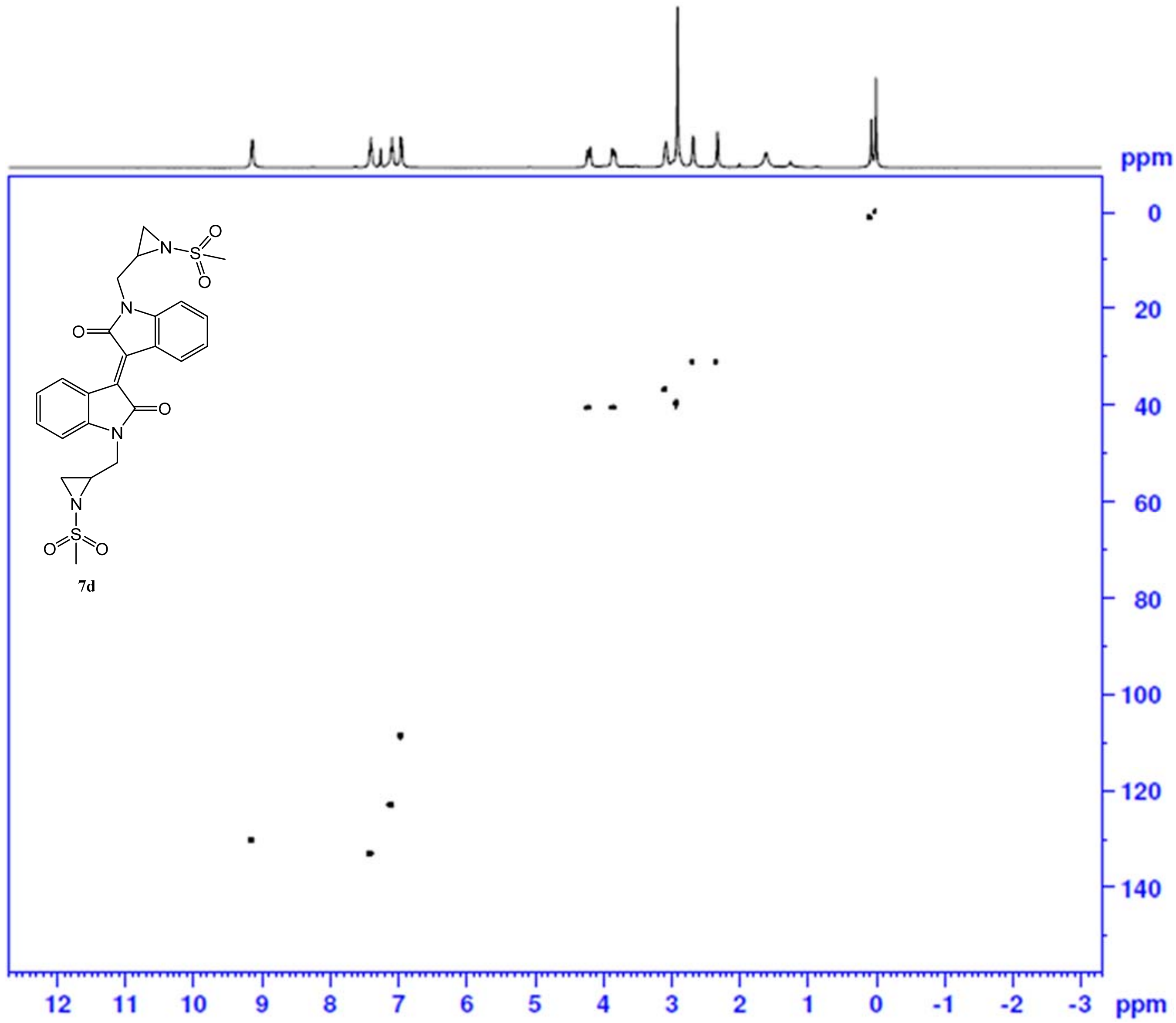

Figure S11. ^{1}H and ^{13}C NMR spectra of **7e**

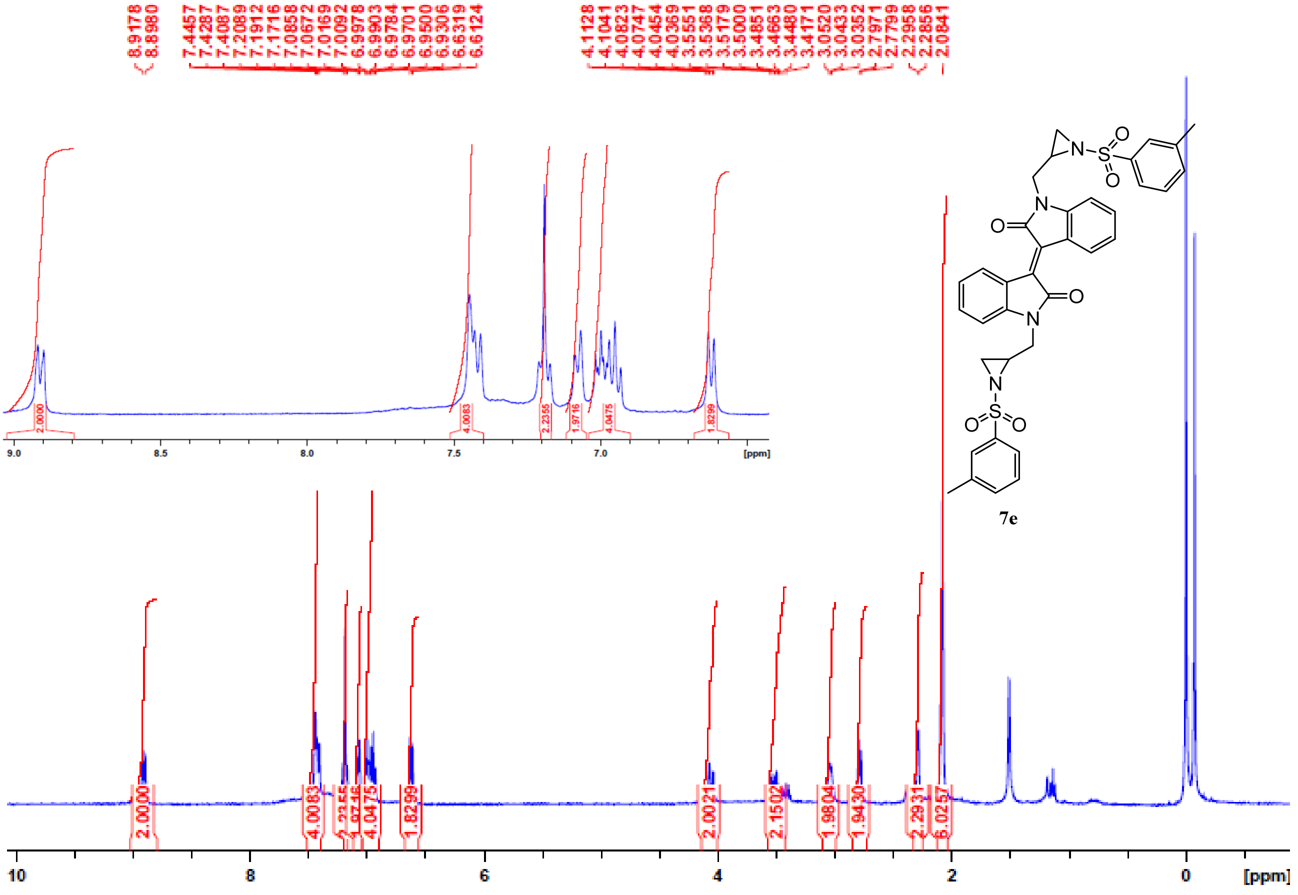

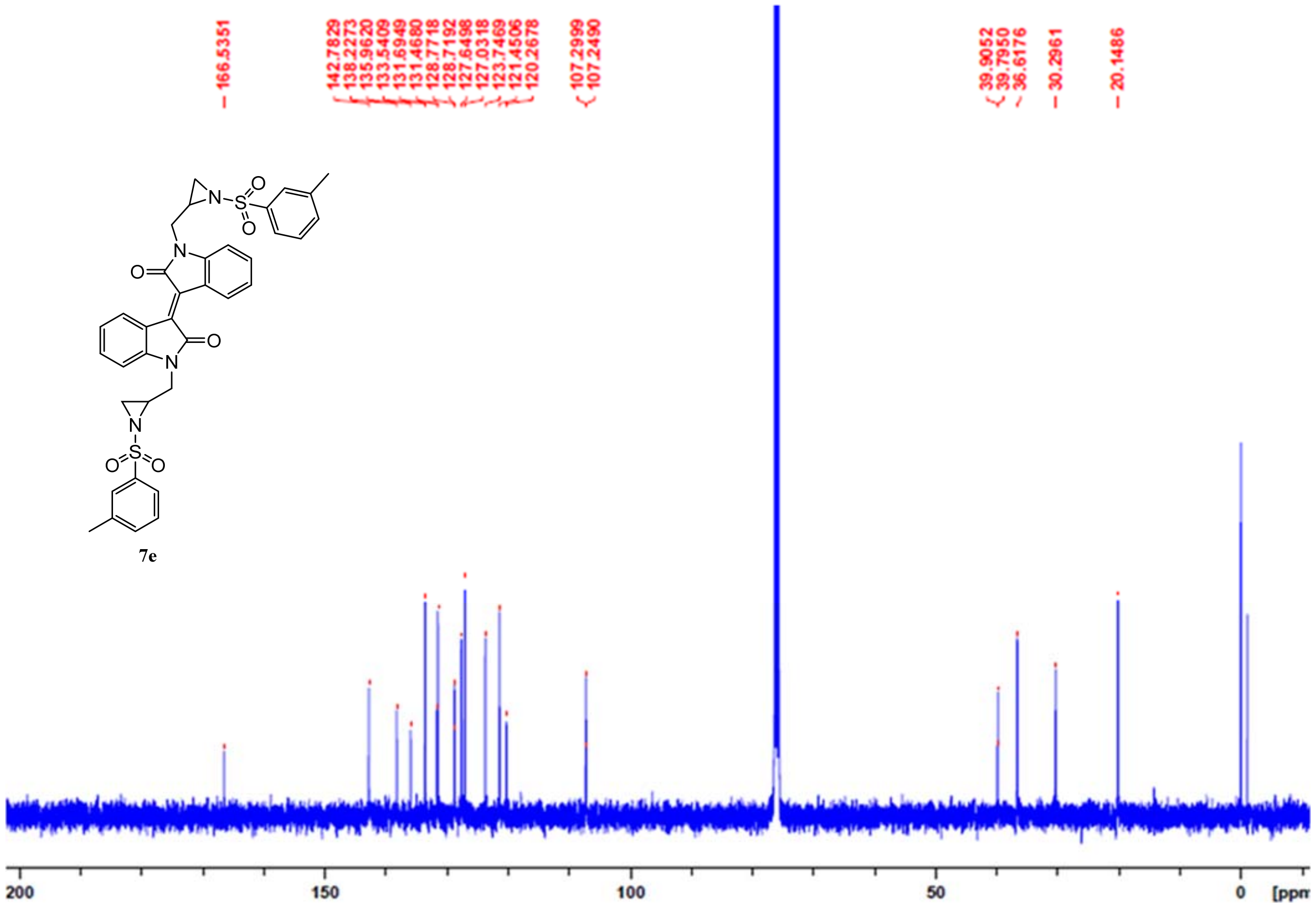

Figure S12. HSQC-NMR spectrum of **7e**

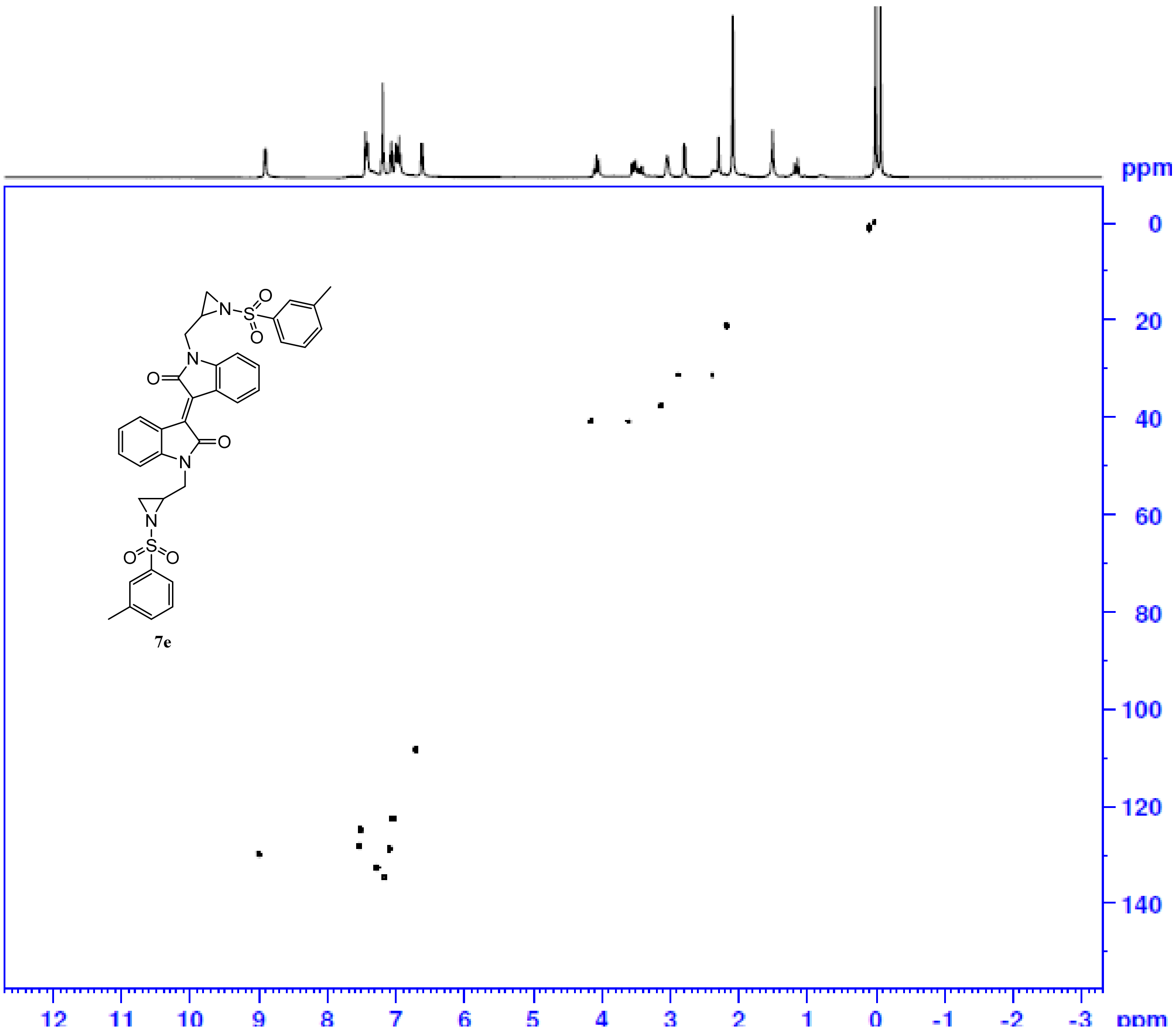

Figure S13. ^{1}H and ^{13}C NMR spectra of **7f**

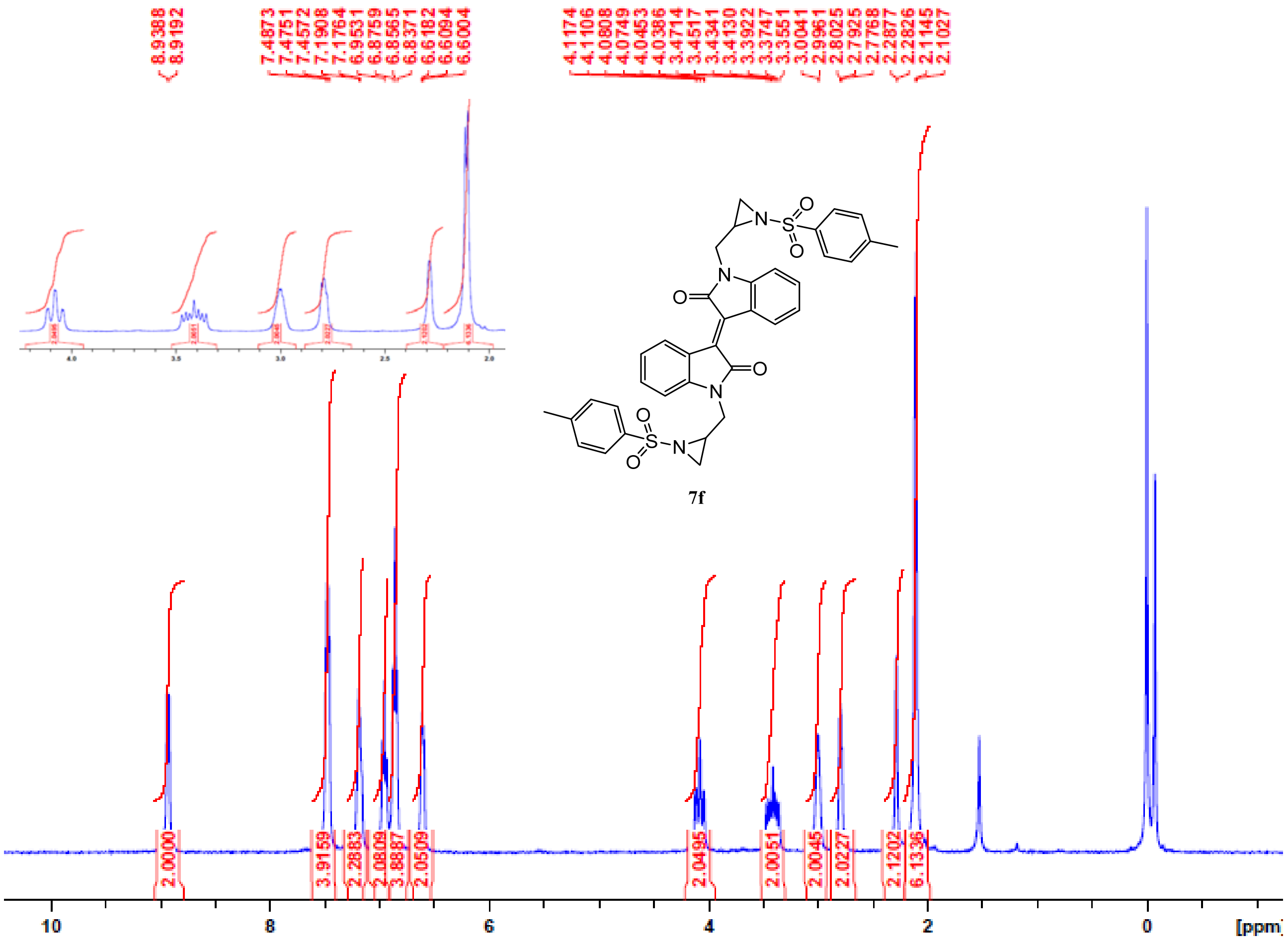

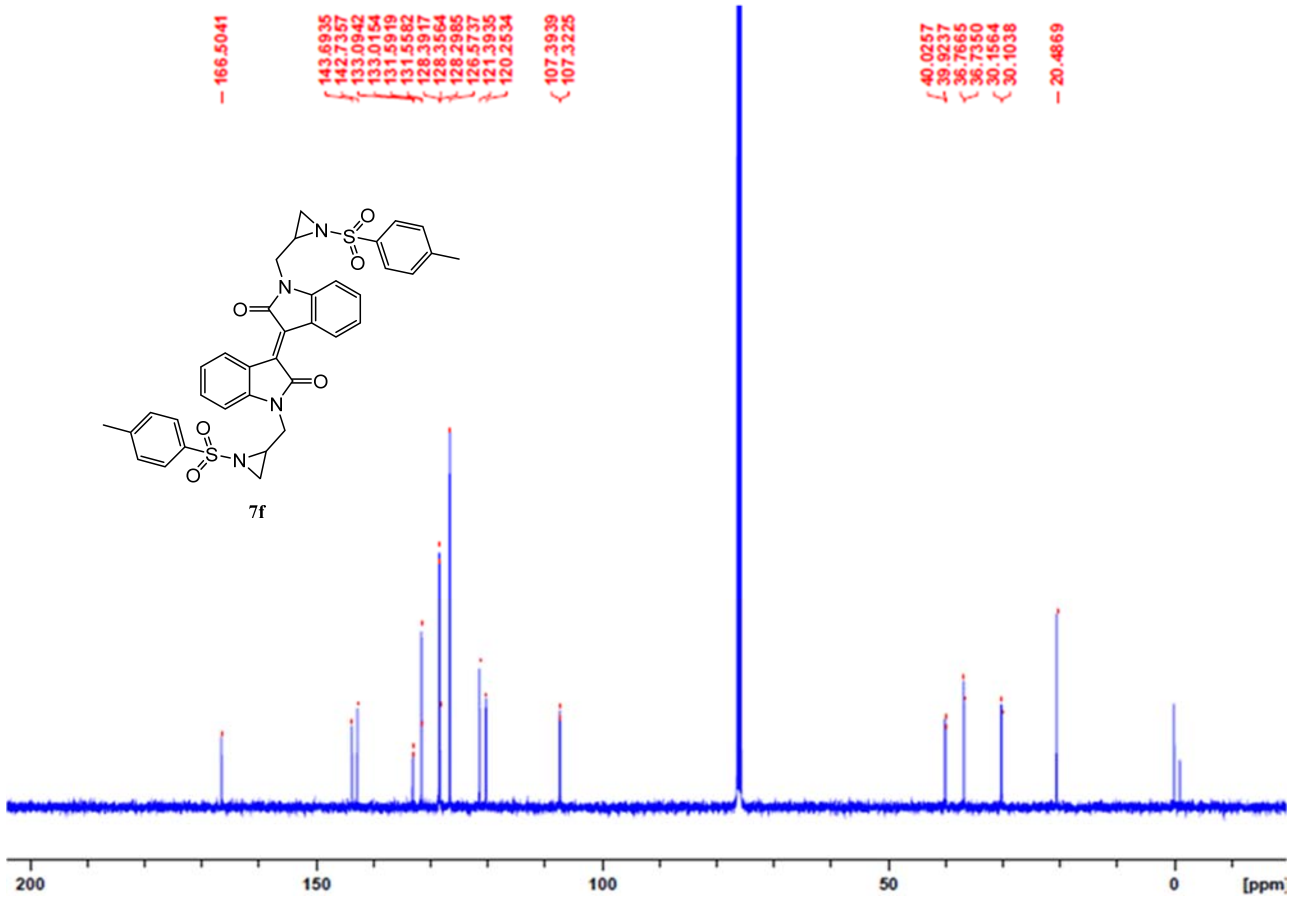

Figure S14. HSQC-NMR spectrum of **7f**

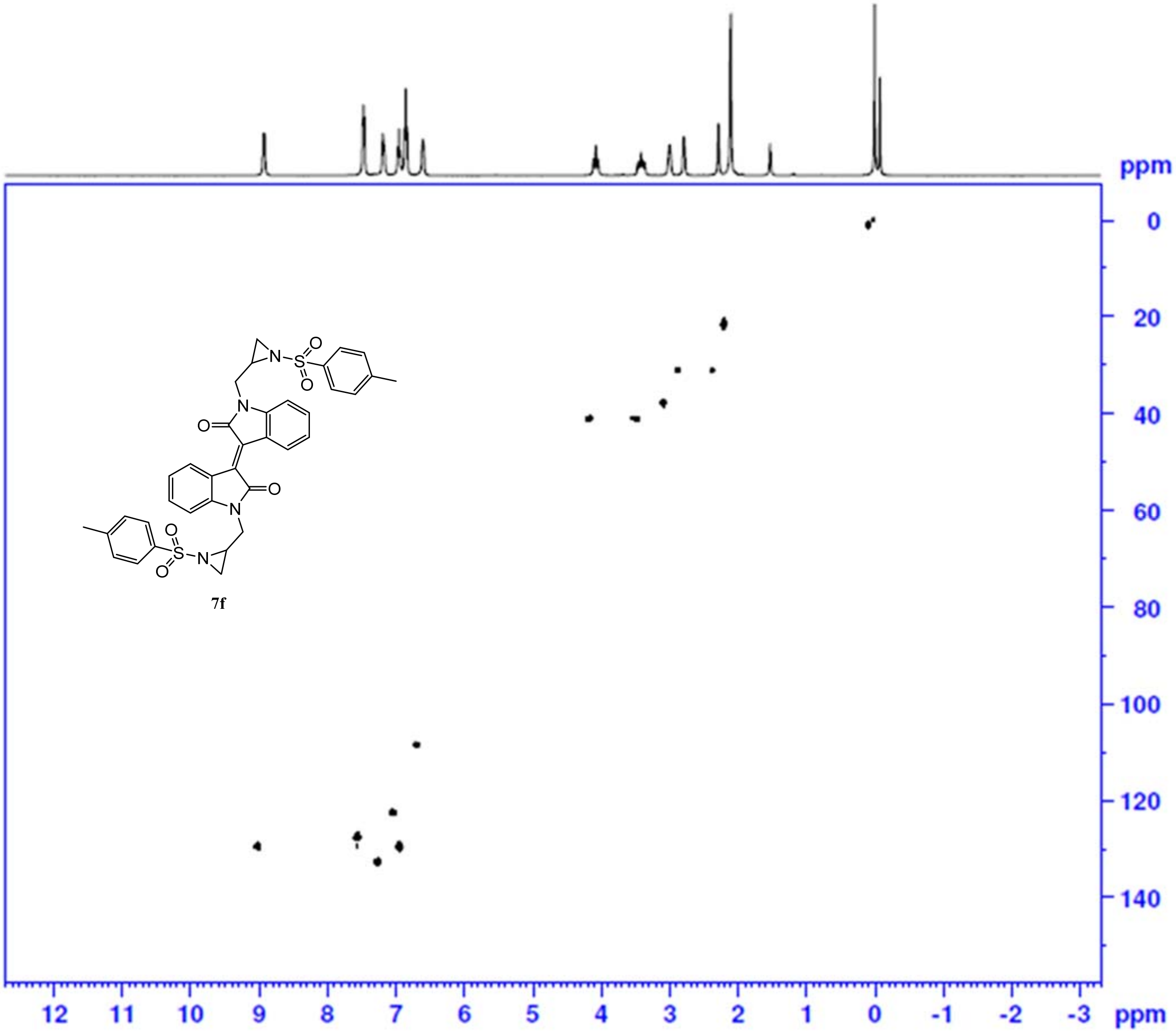

Figure S15. ^{1}H and ^{13}C NMR spectra of **7g**

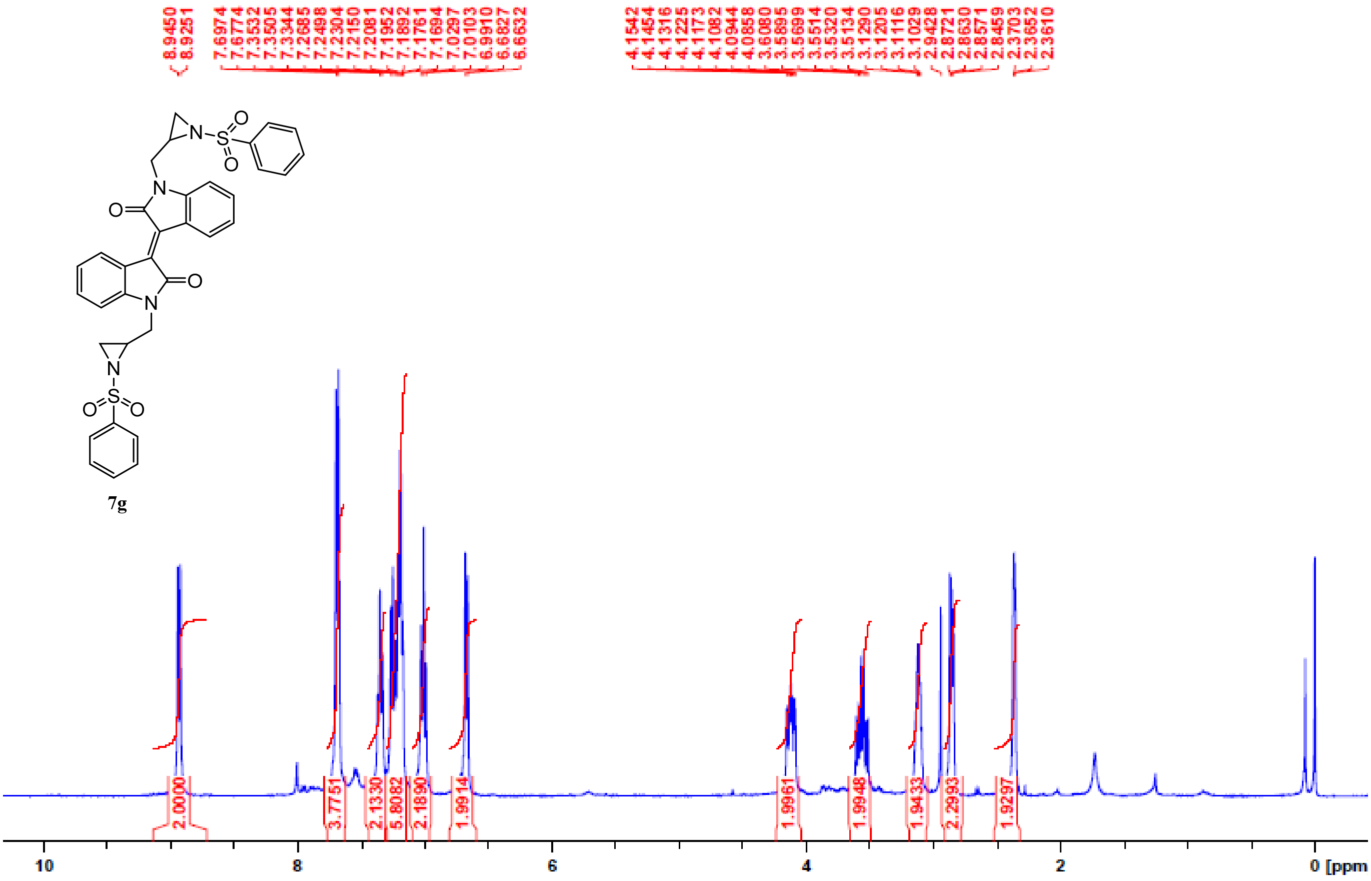

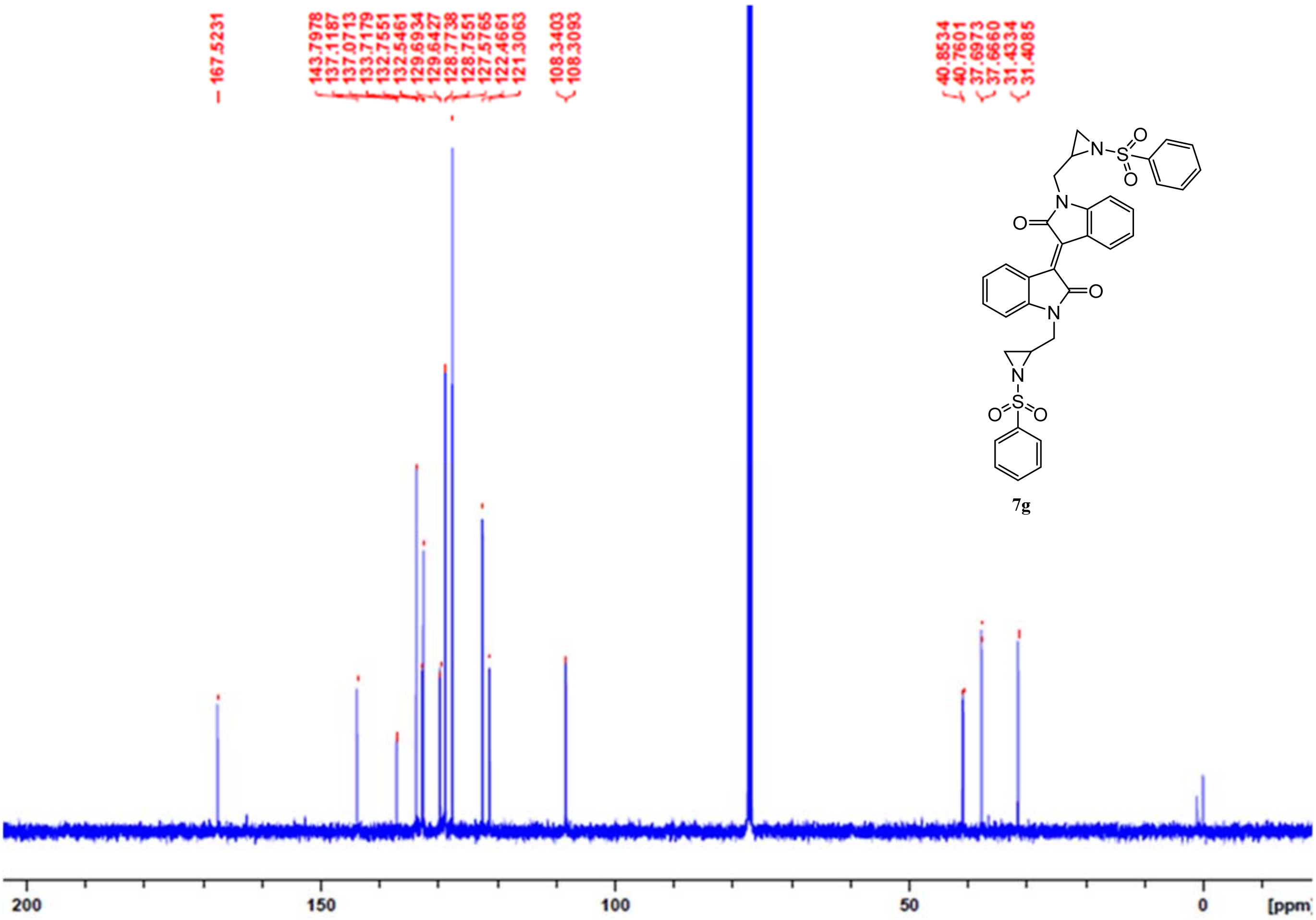

7g

Figure S16. HSQC and COSY-NMR spectrum of **7g**

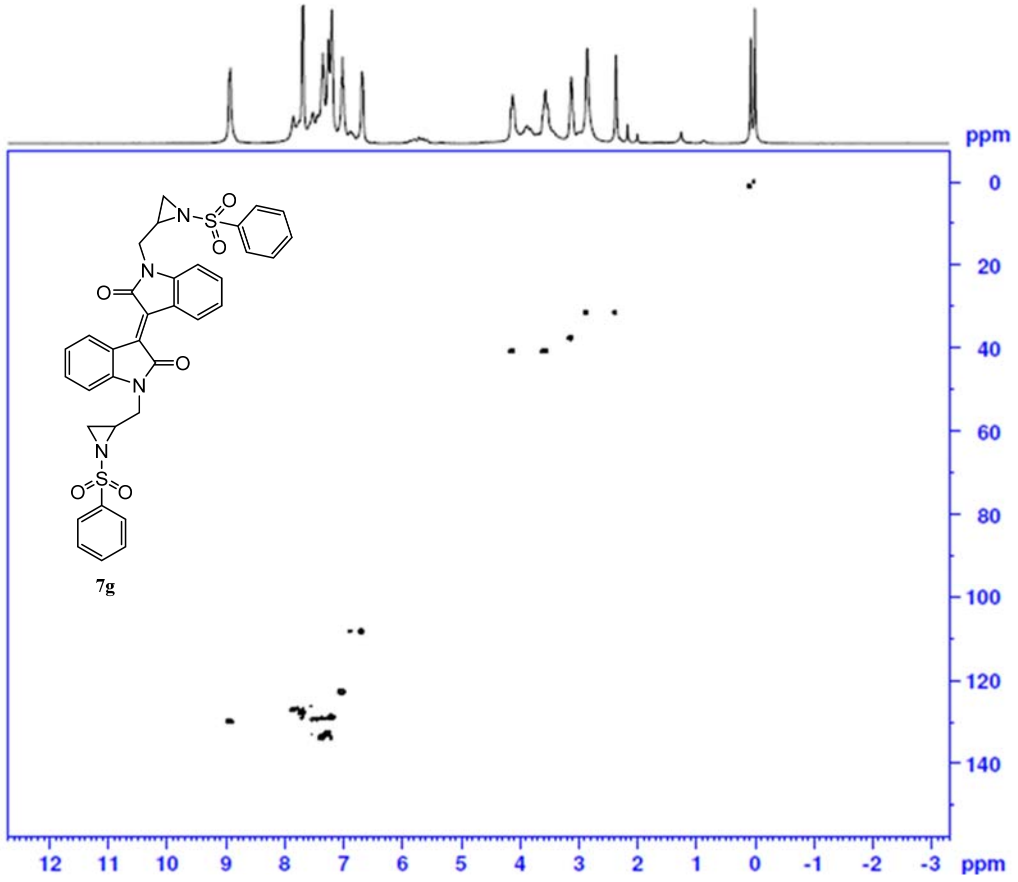

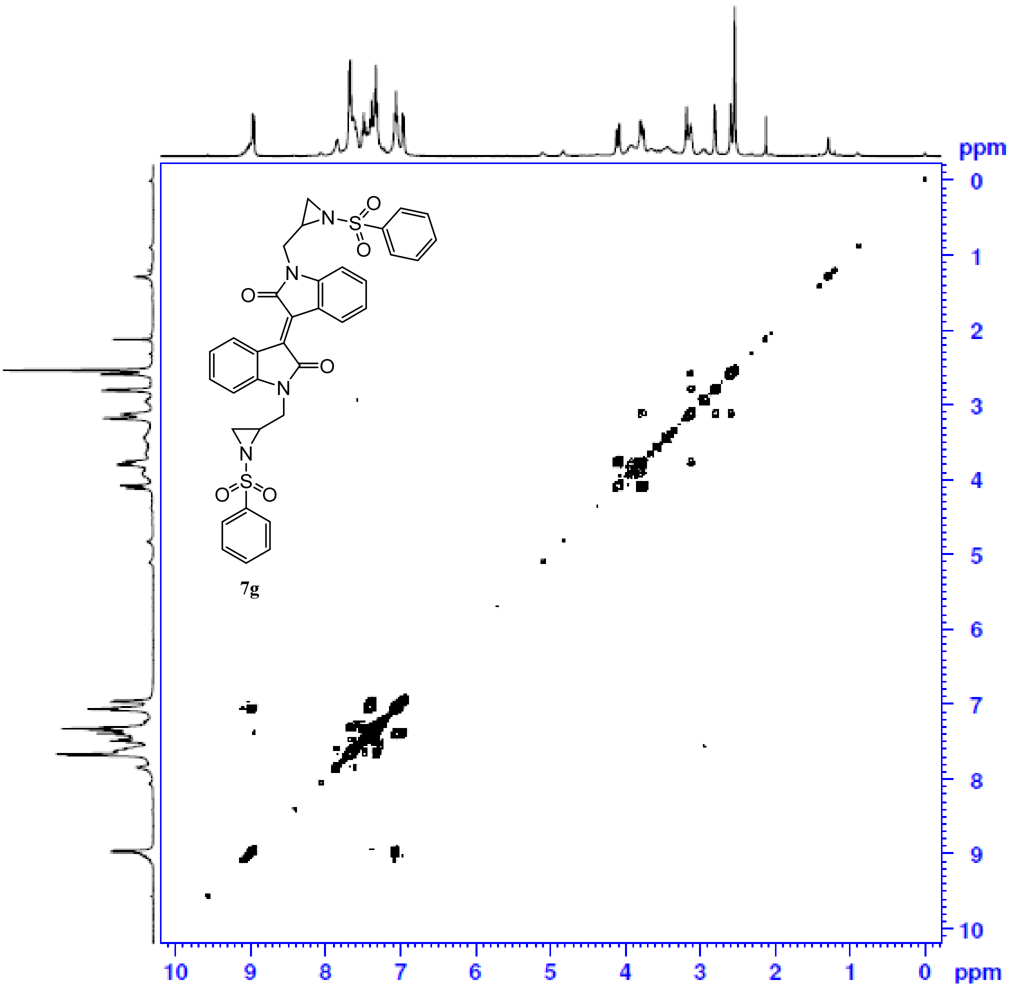

Figure S17. ^{1}H and ^{13}C NMR spectra of **7h**

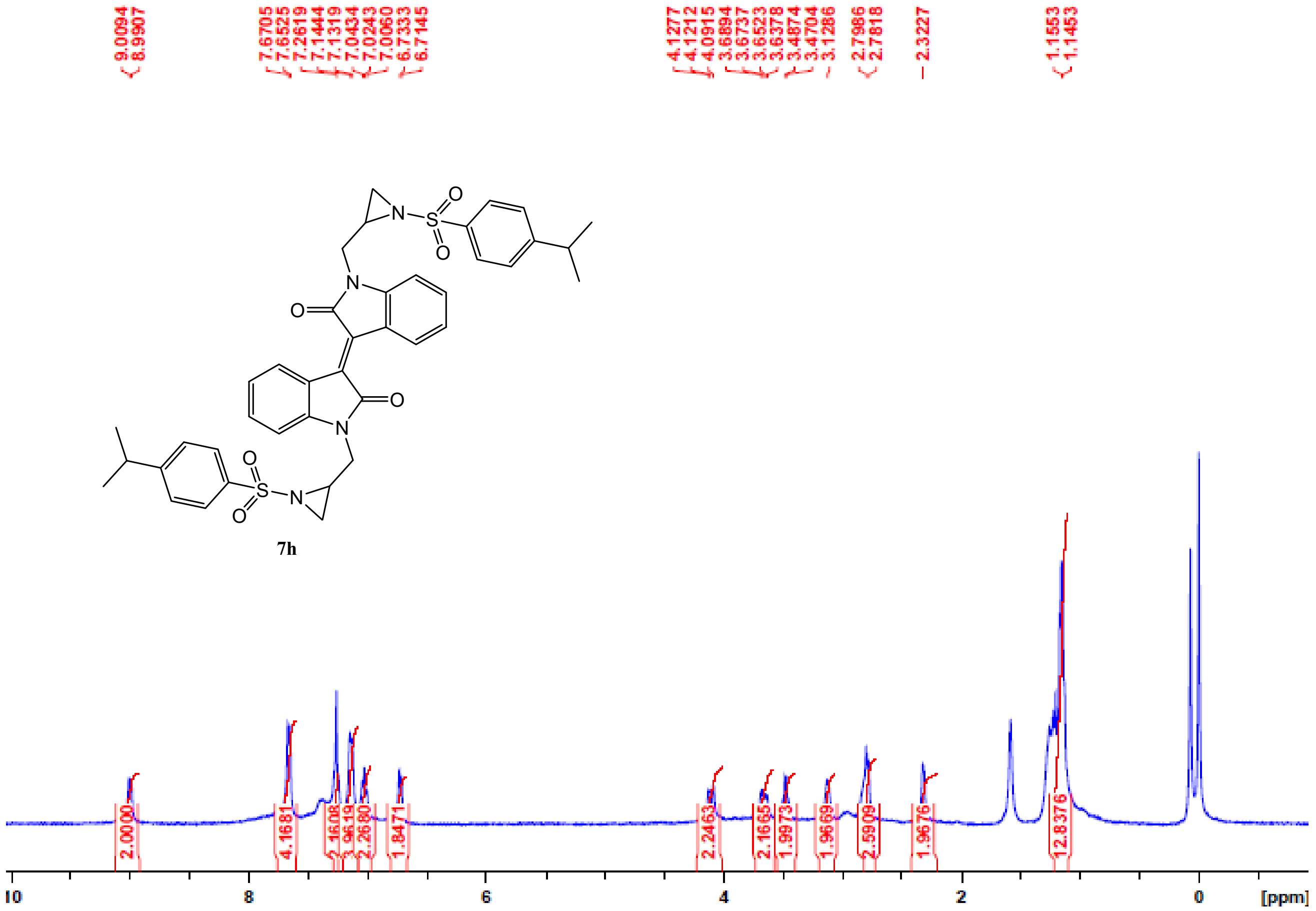

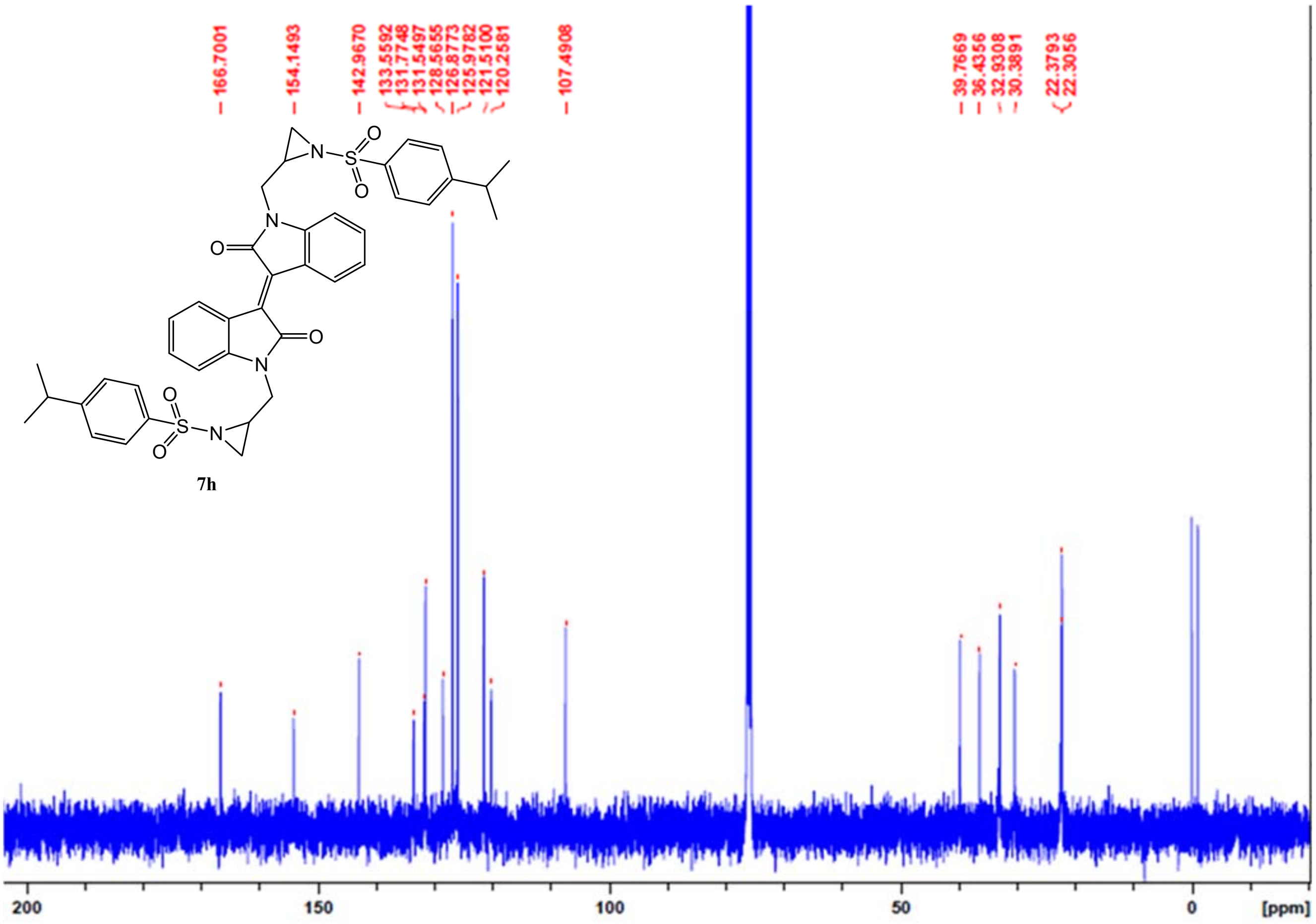

Figure S18. HSQC -NMR spectrum of **7h**

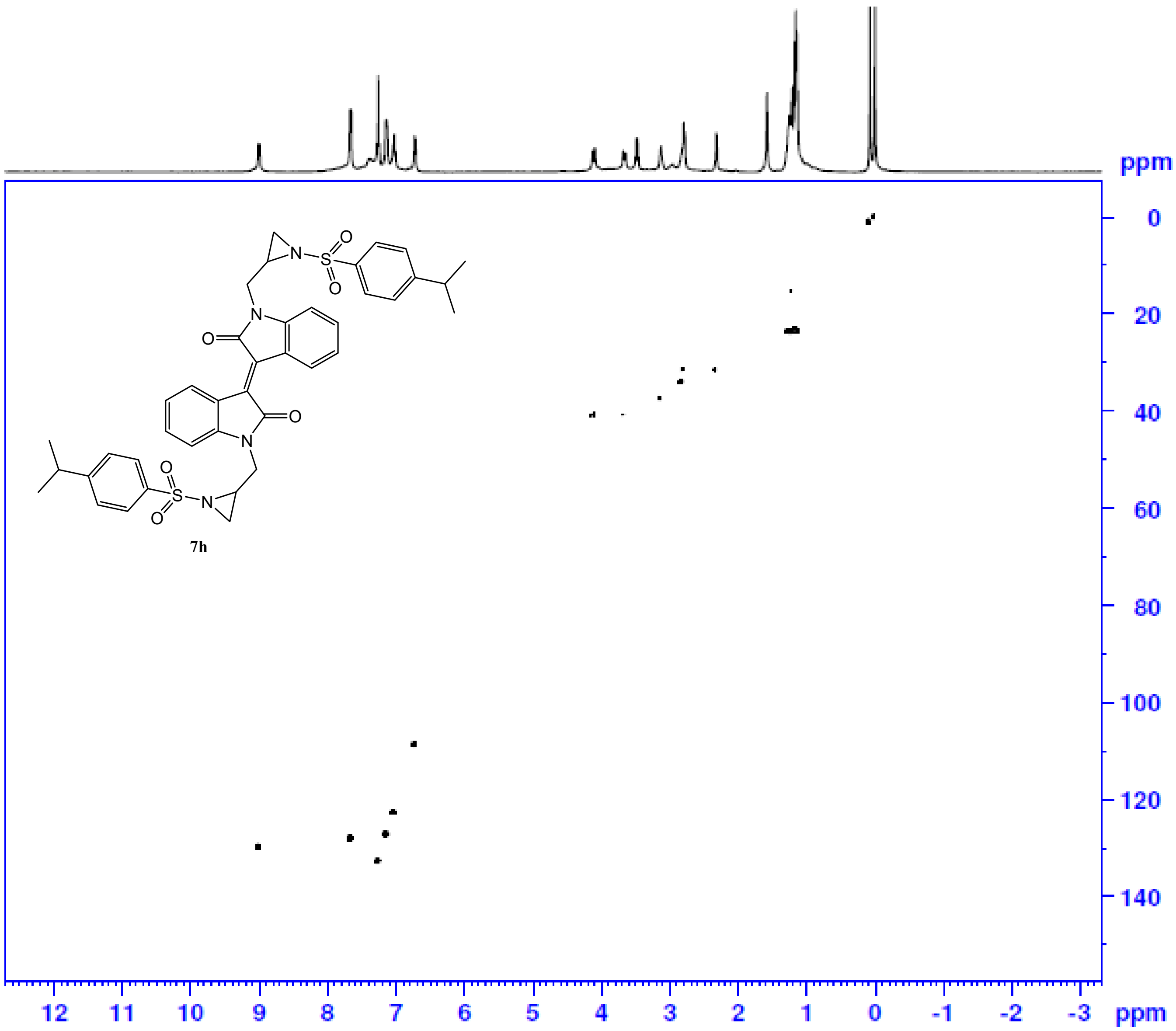

Figure S19. ^1H and ^{13}C NMR spectra of **7i**

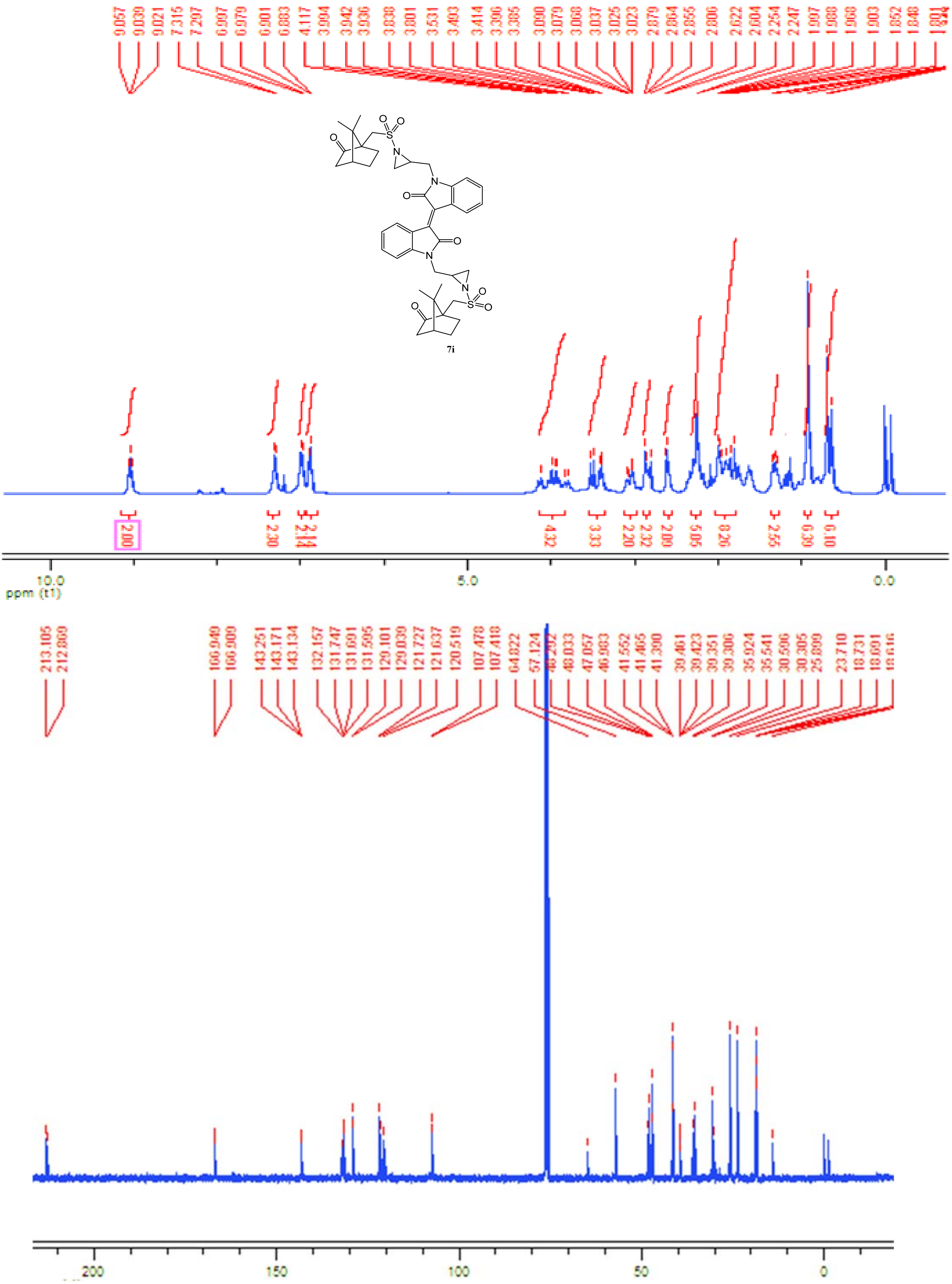

Figure S20. ^{1}H NMR spectra expansions of **7i**

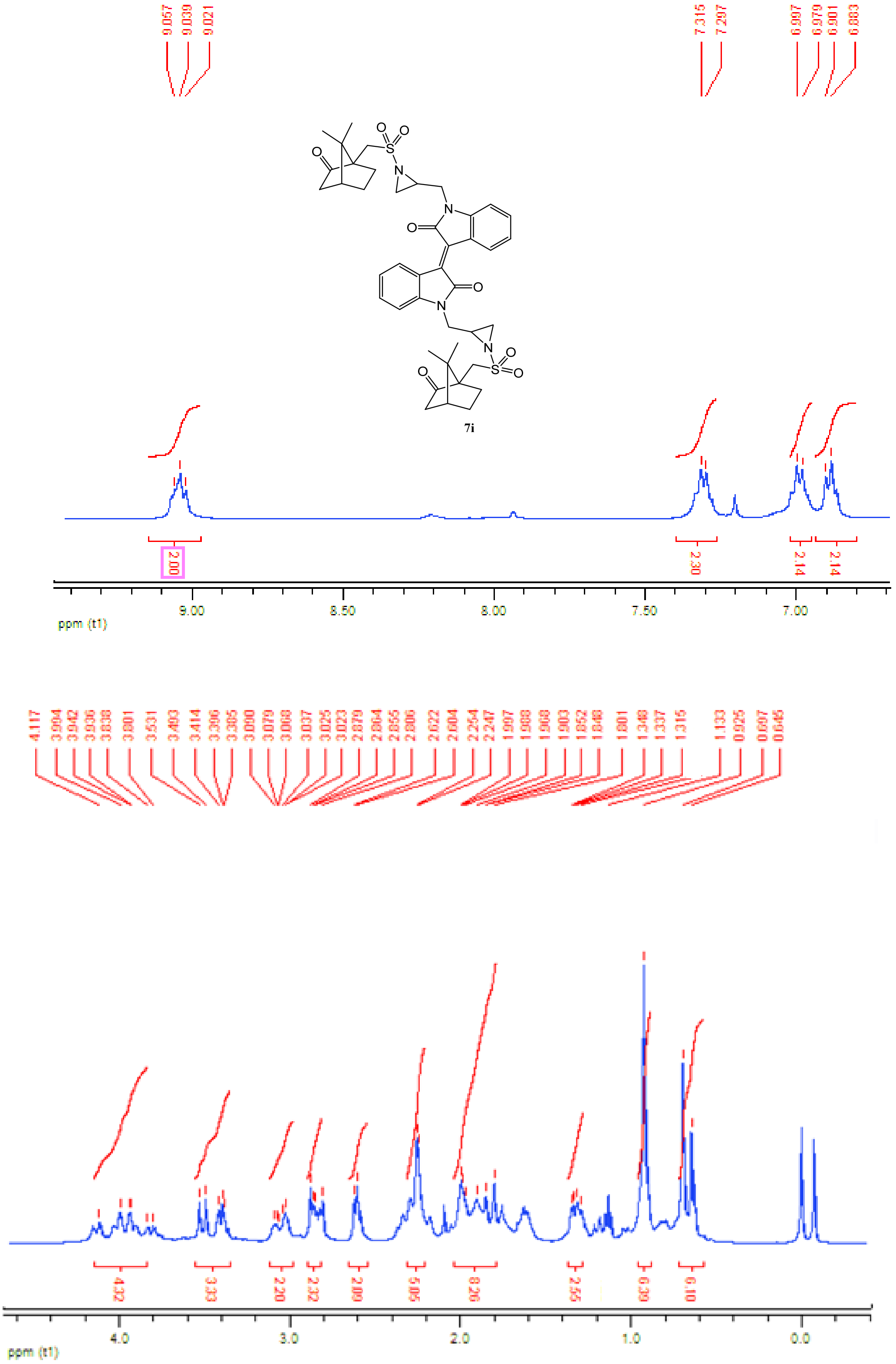

Figure S21. HSQC-NMR spectrum of **7i**

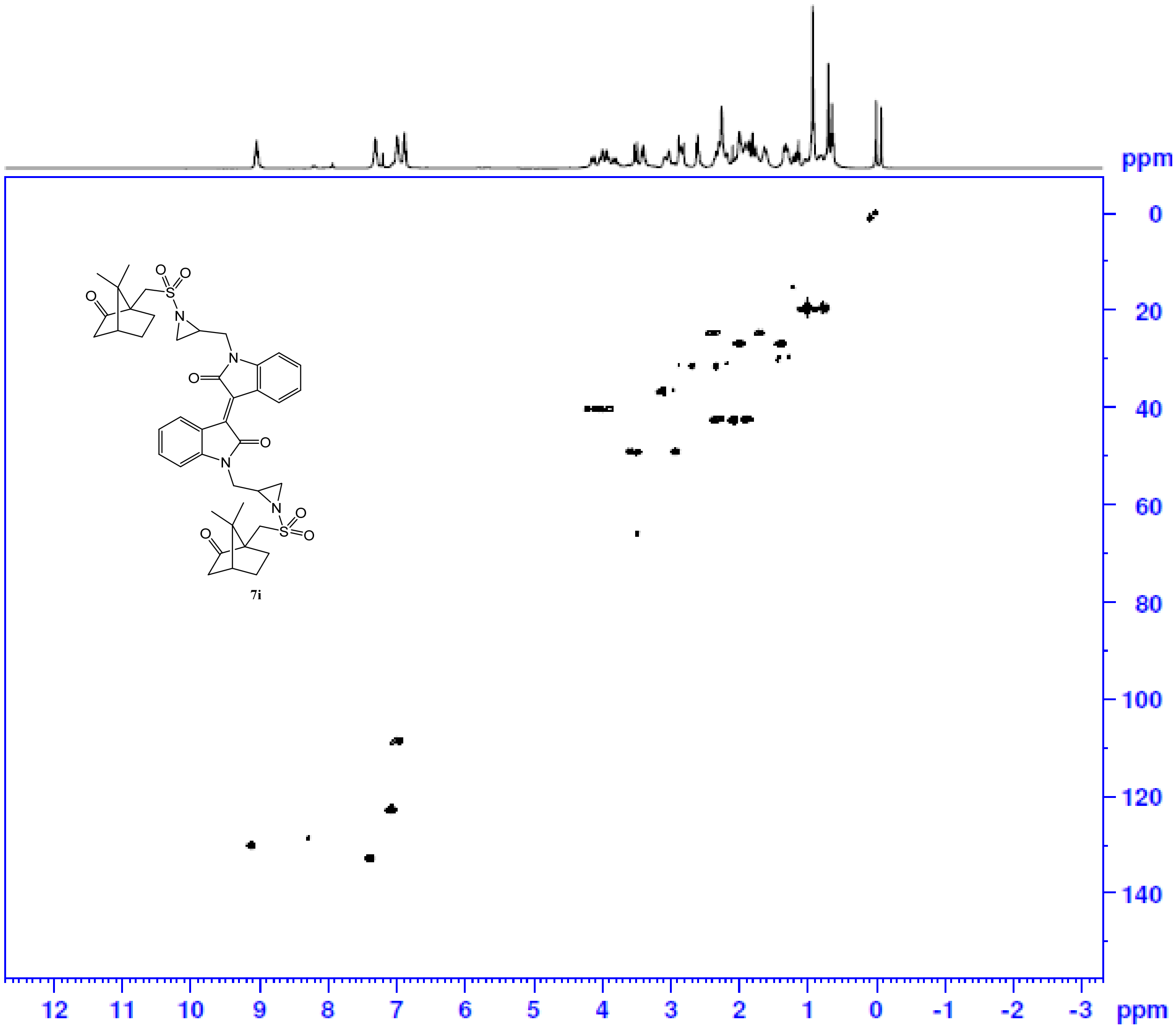

Figure S22. ^{1}H and ^{13}C NMR spectra of **6d**

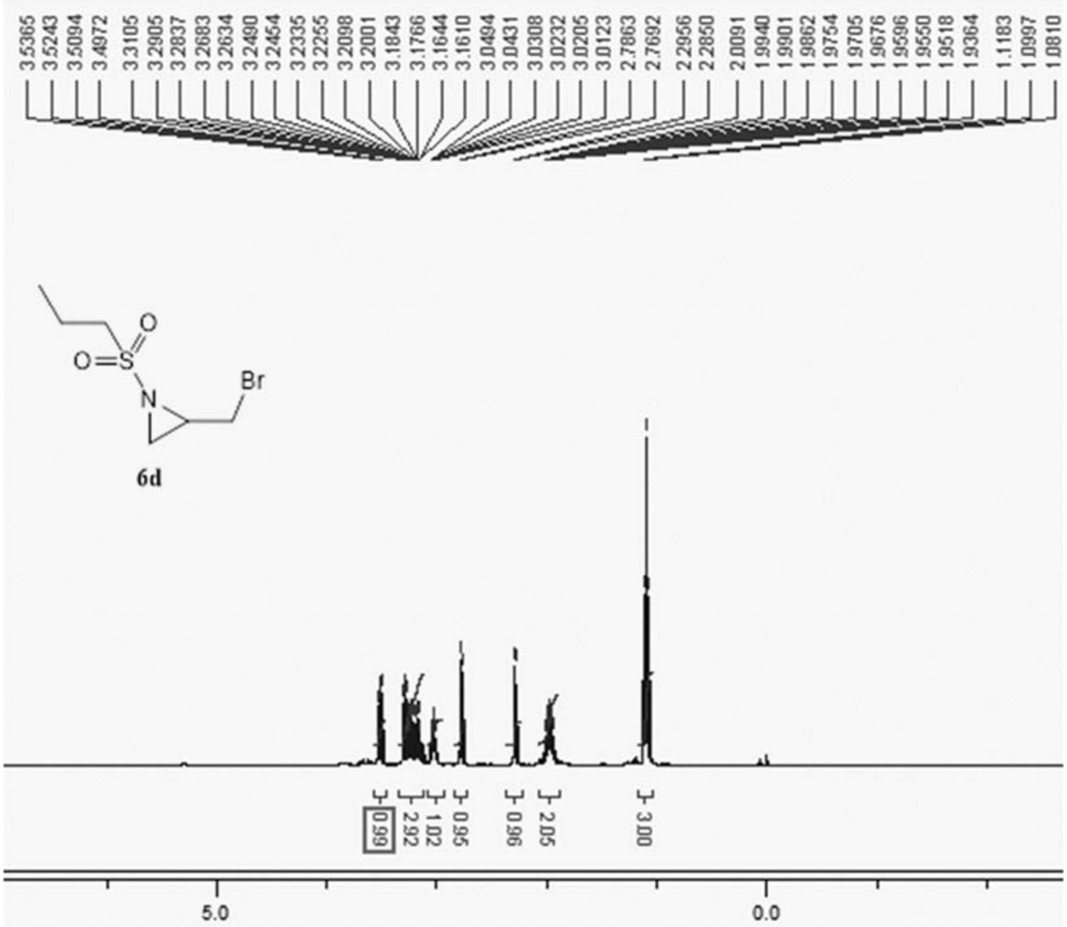

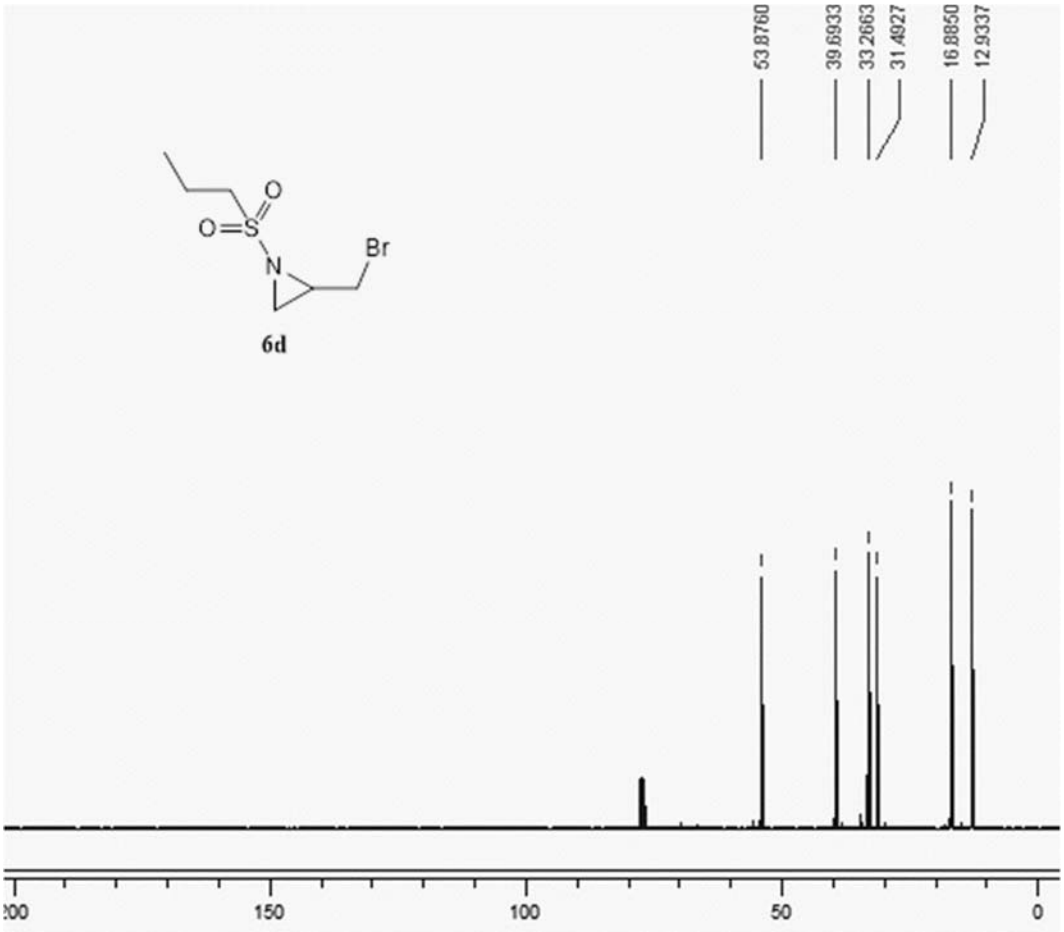

Figure S23. ^1H and ^{13}C NMR spectra of **6e**

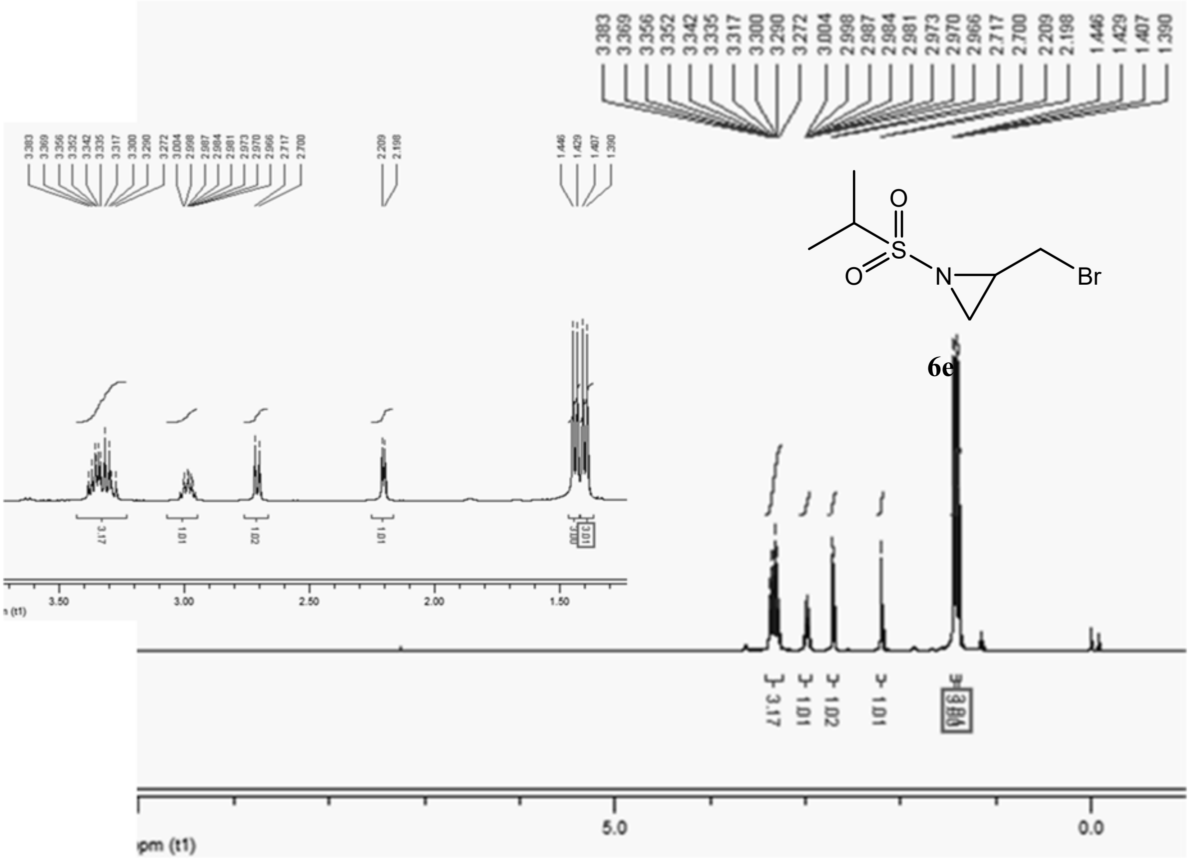

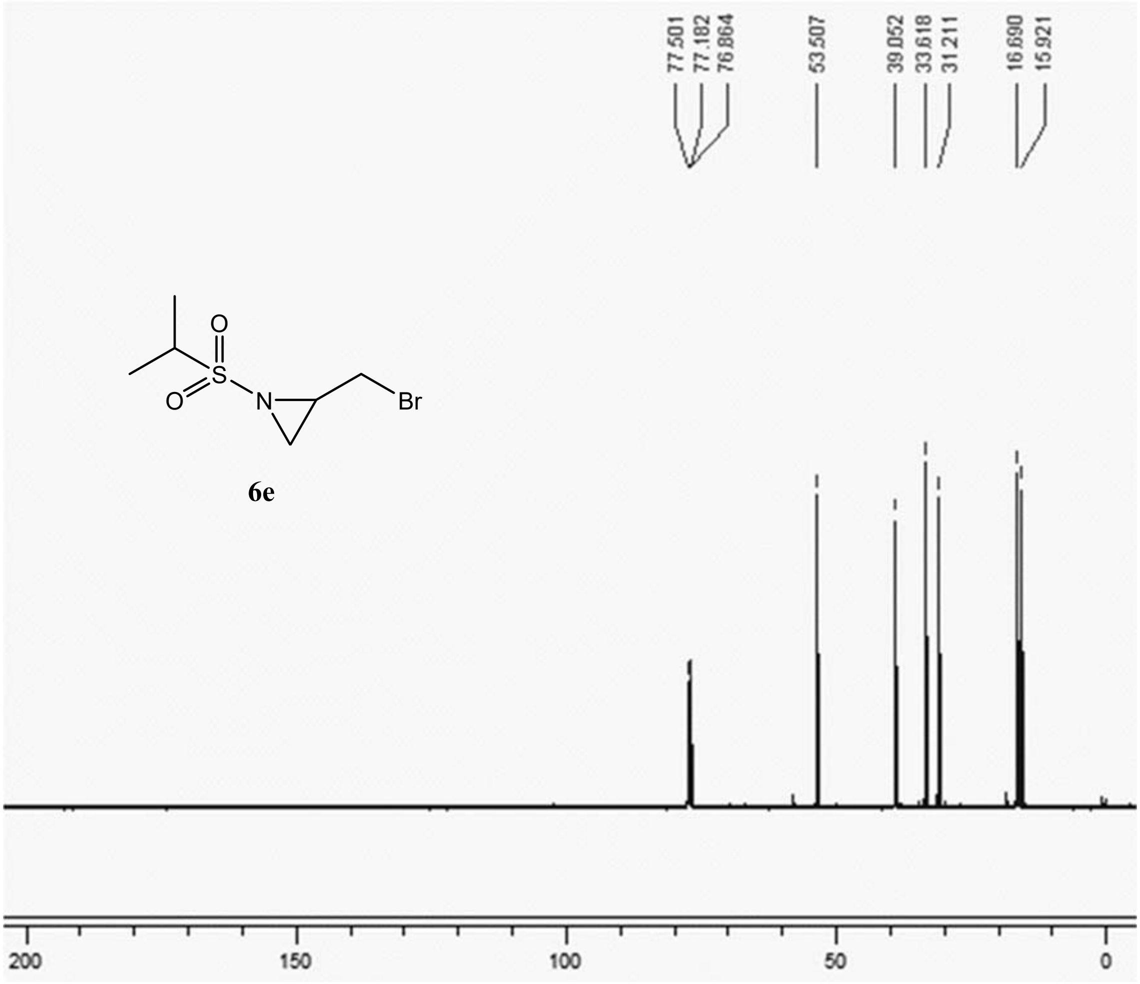

Figure S24. ^{1}H and ^{13}C NMR spectra of **6f**

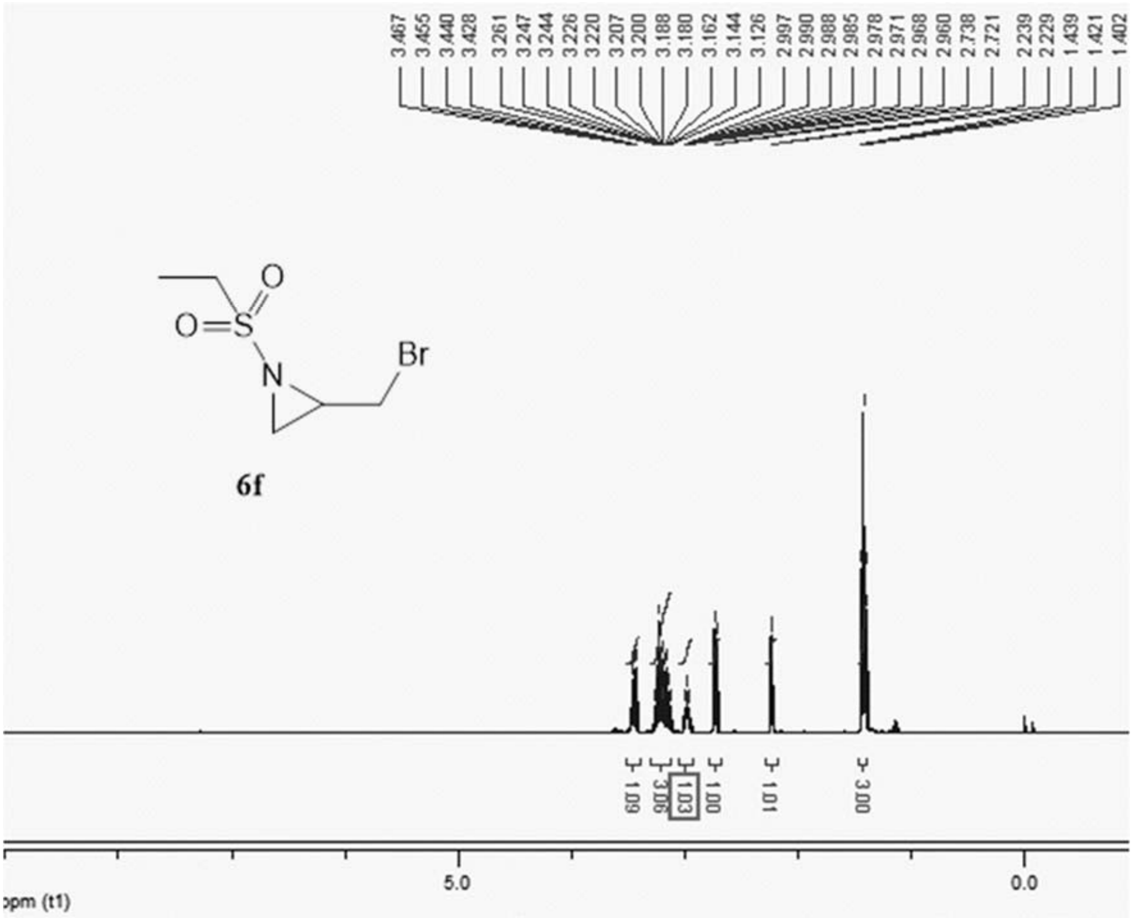

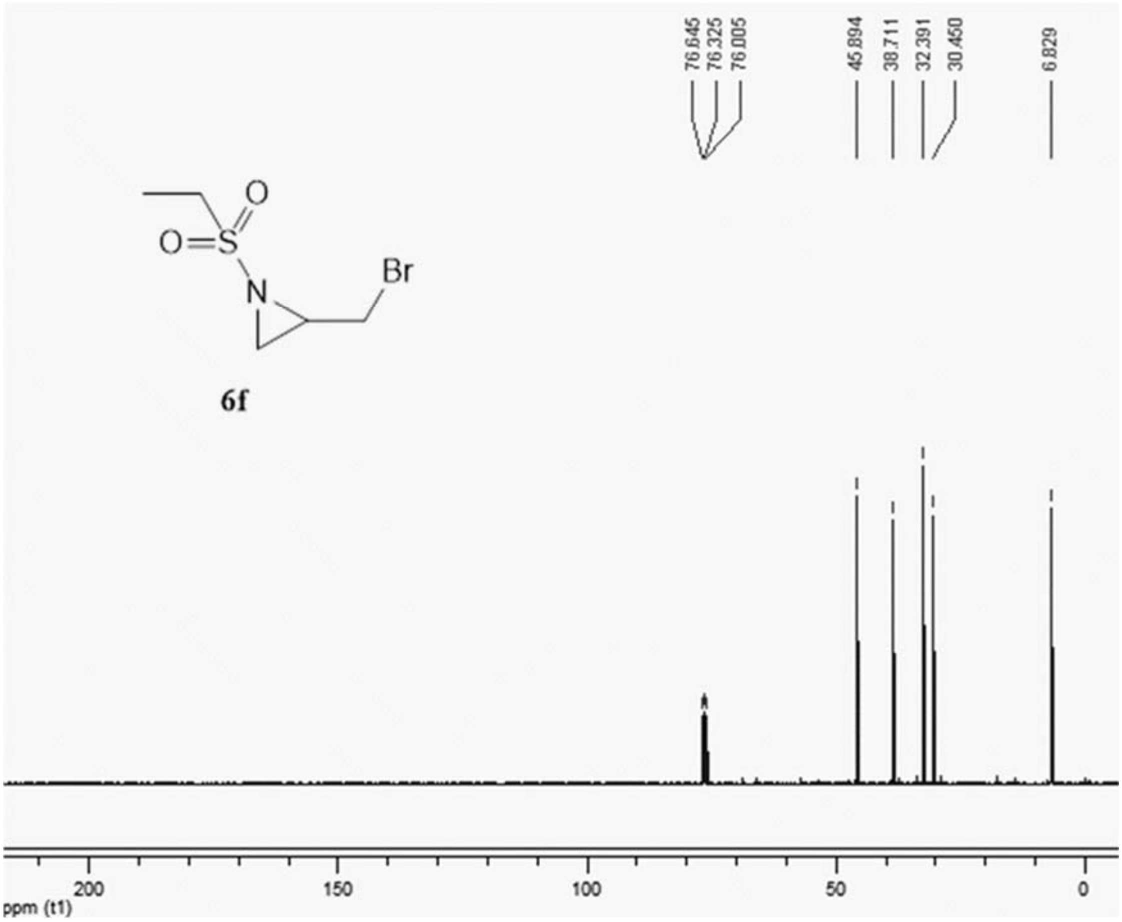

Figure S25. ^{1}H and ^{13}C NMR spectra of **6g**

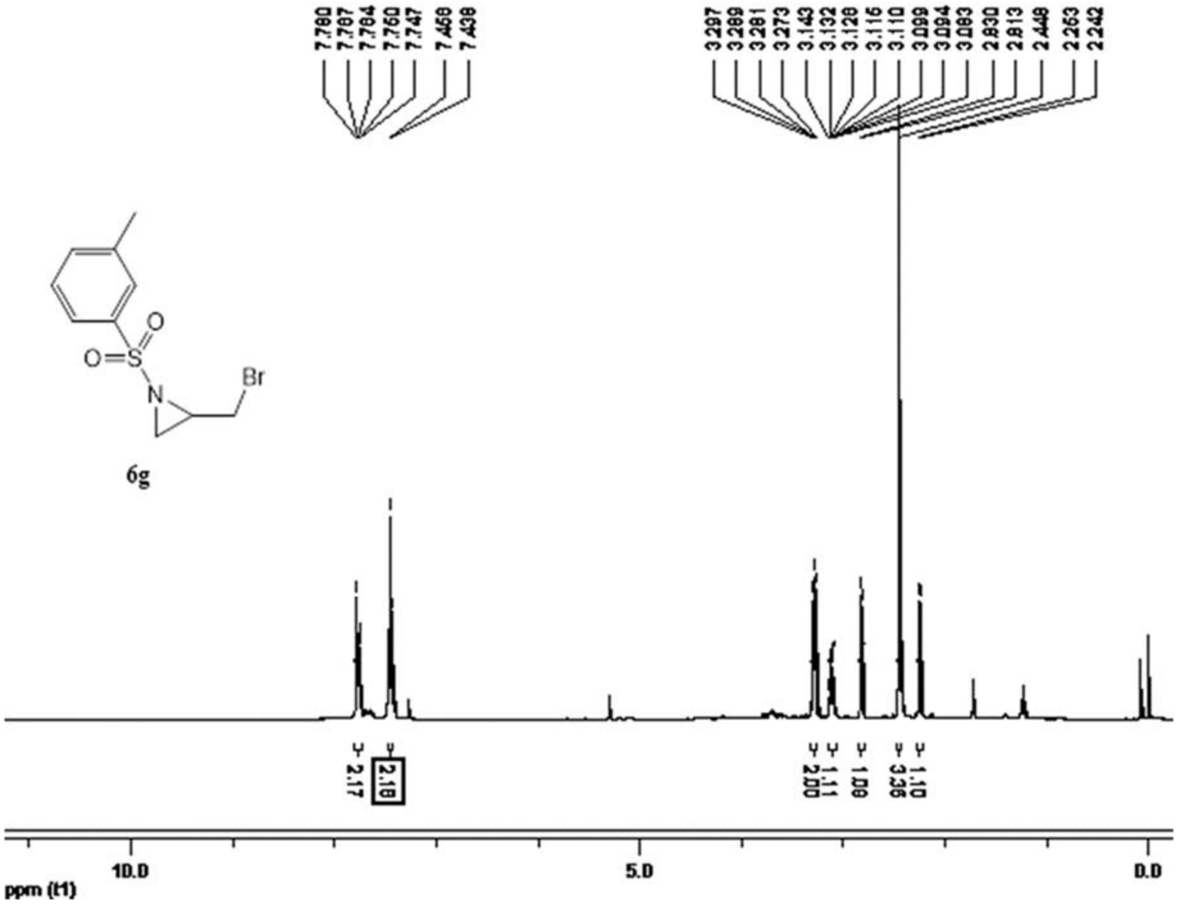

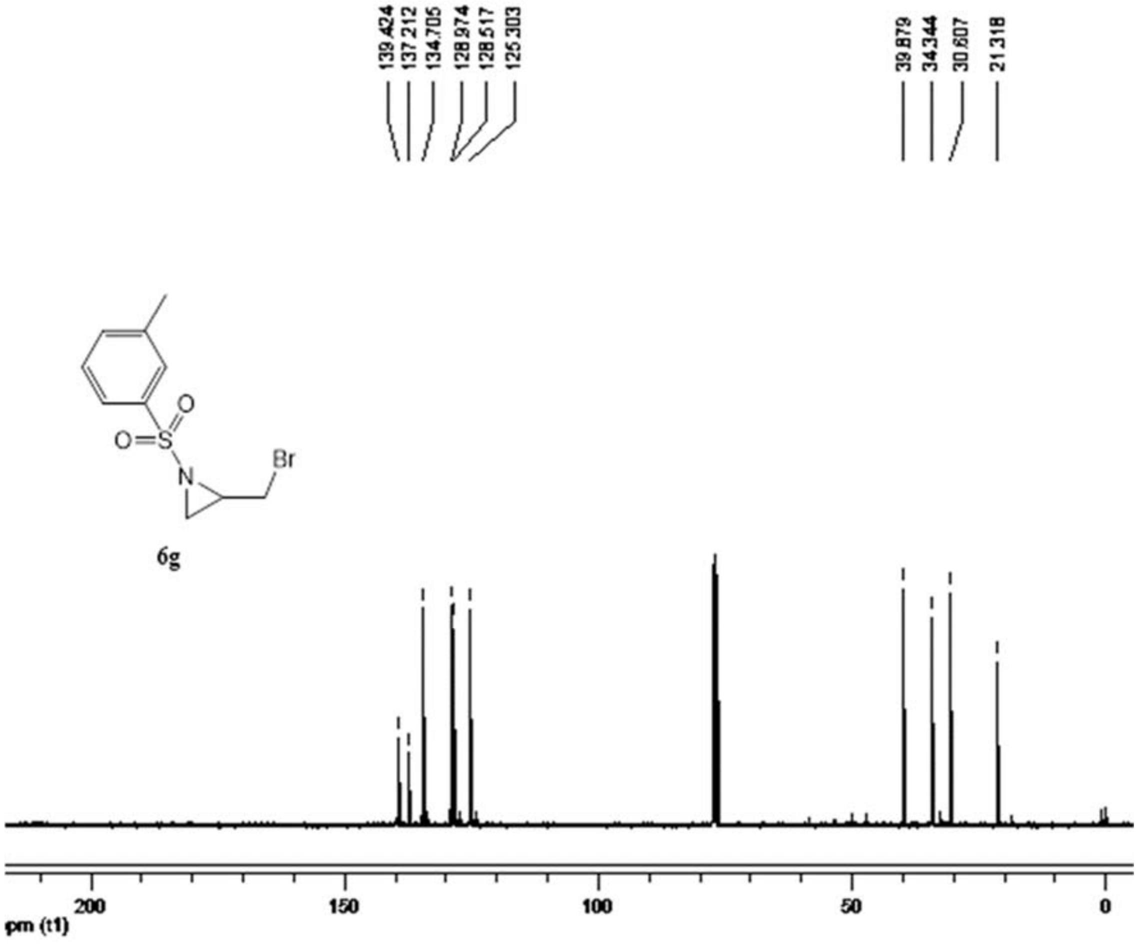

Figure S26. ^{1}H and ^{13}C NMR spectra of **6h**

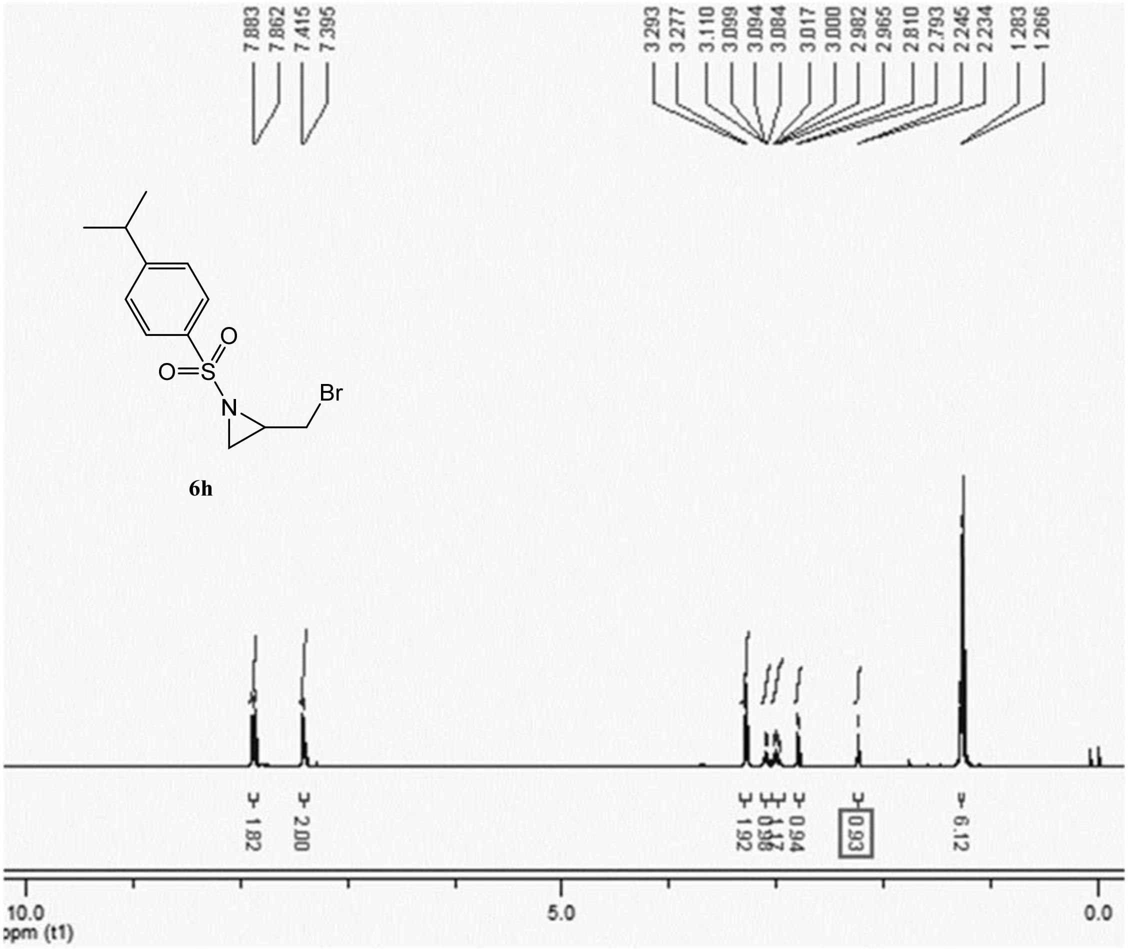

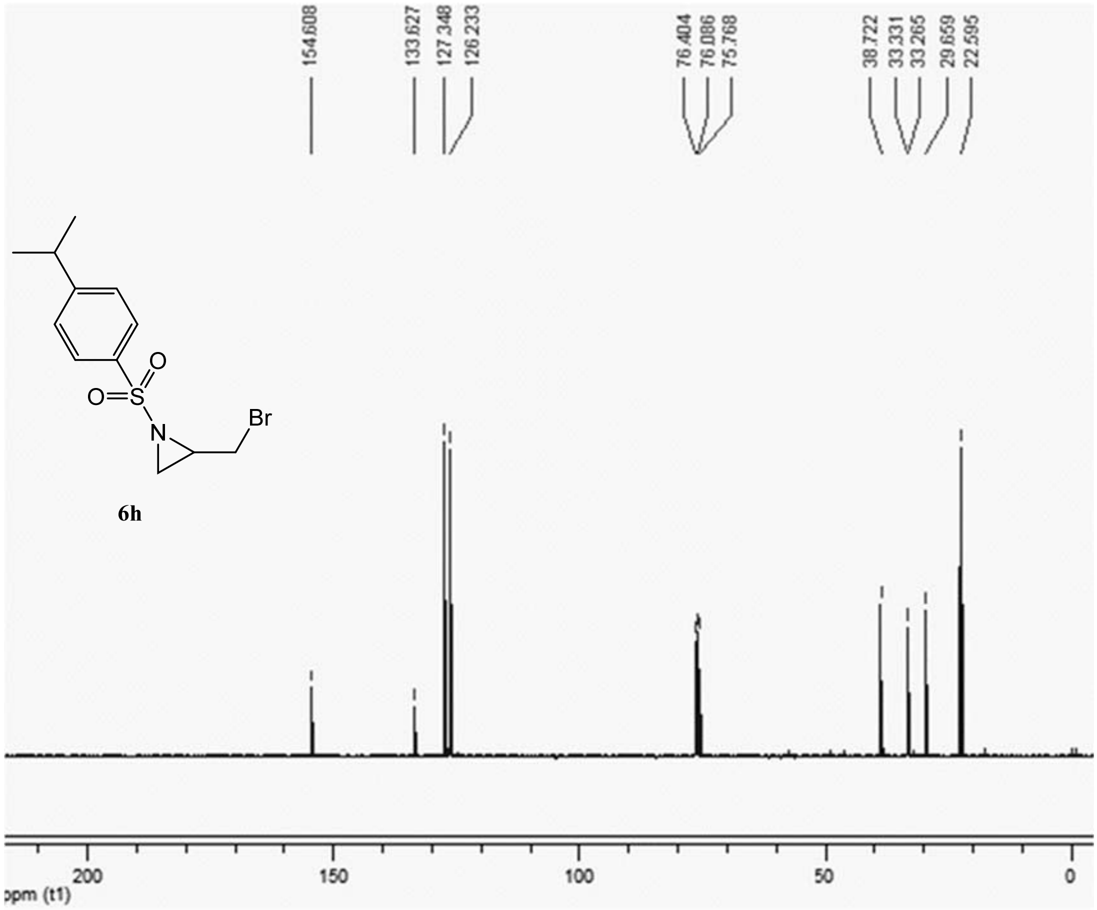

Figure S27. ^{1}H and ^{13}C NMR spectra of **6i**

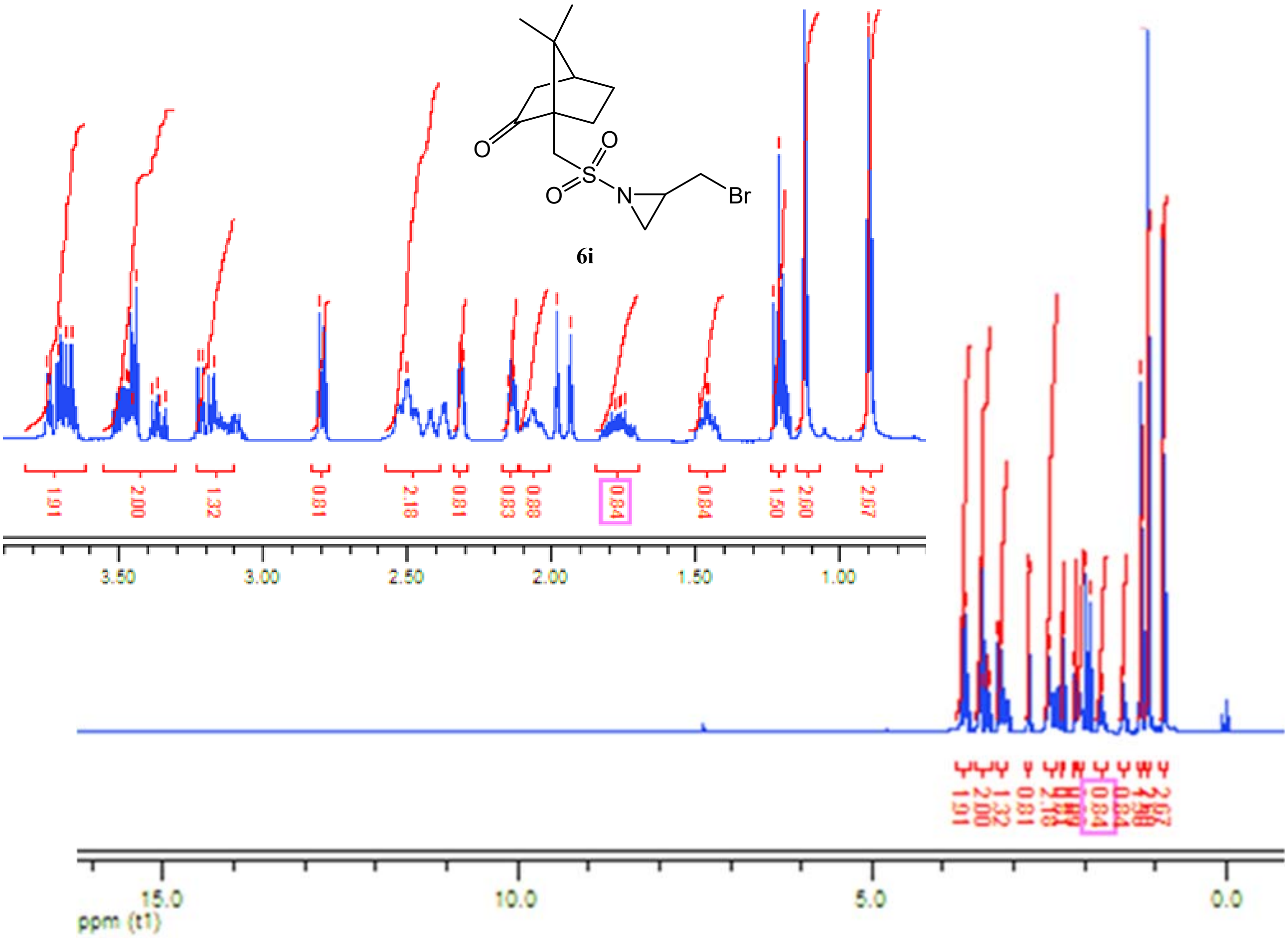

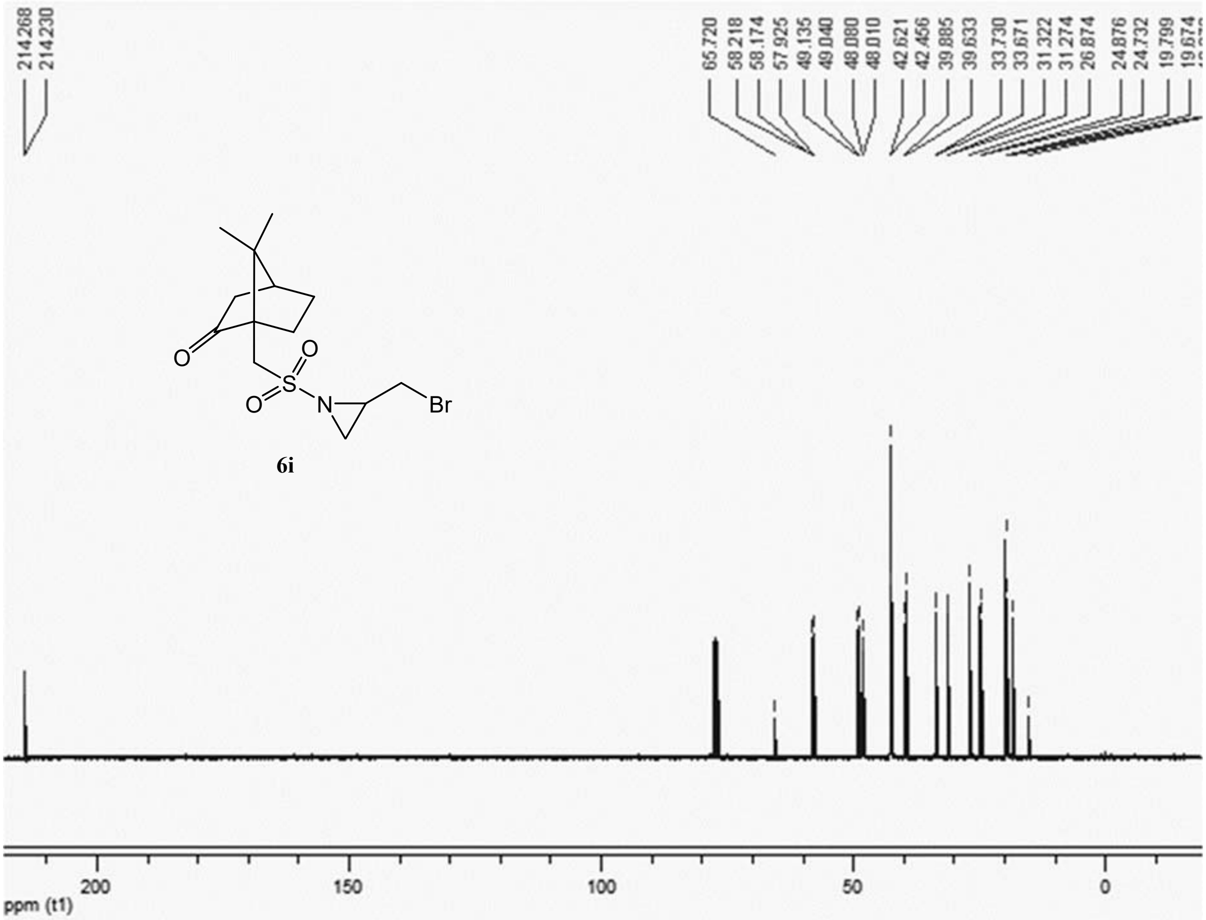

Figure S28. Modelled UV-Vis spectra for monomer thiophene-*N*,*N*'-substituted-isoindigo compounds, calculated with TD-DFT a) B3LYP/6-31+G(2d,p) and b) CAM-B3LYP/6-31+G(2d,p).

a

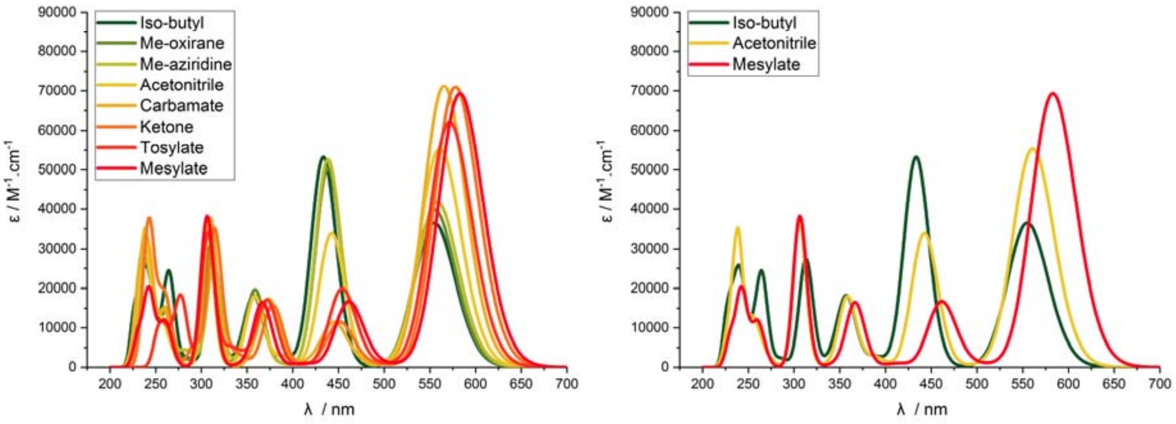

b

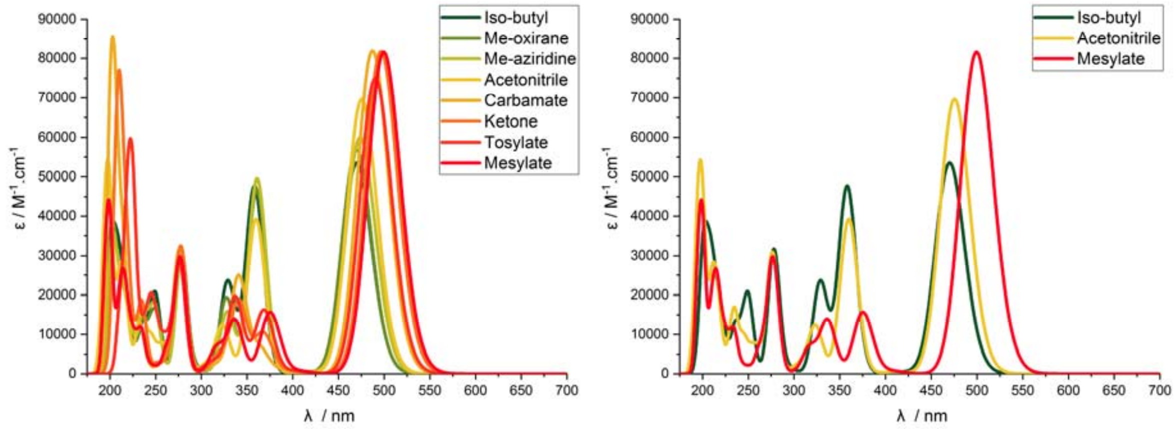

Figure S29. Modelled UV-Vis spectra for dimer thiophene-N,N´substituted-isoindigo compounds, calculated with TD-DFT a) B3LYP/6-31+G(2d,p) and b) CAM-B3LYP/6-31+G(2d,p)

a

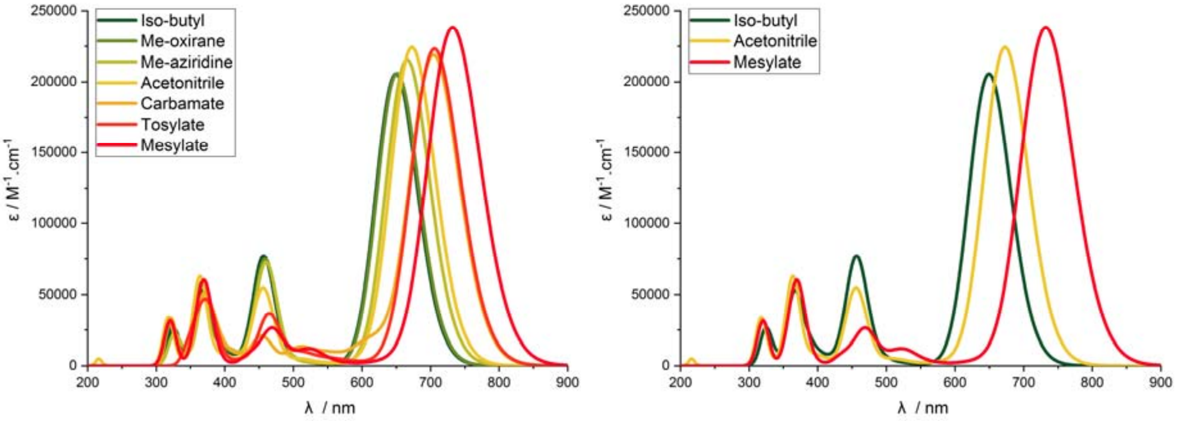

b

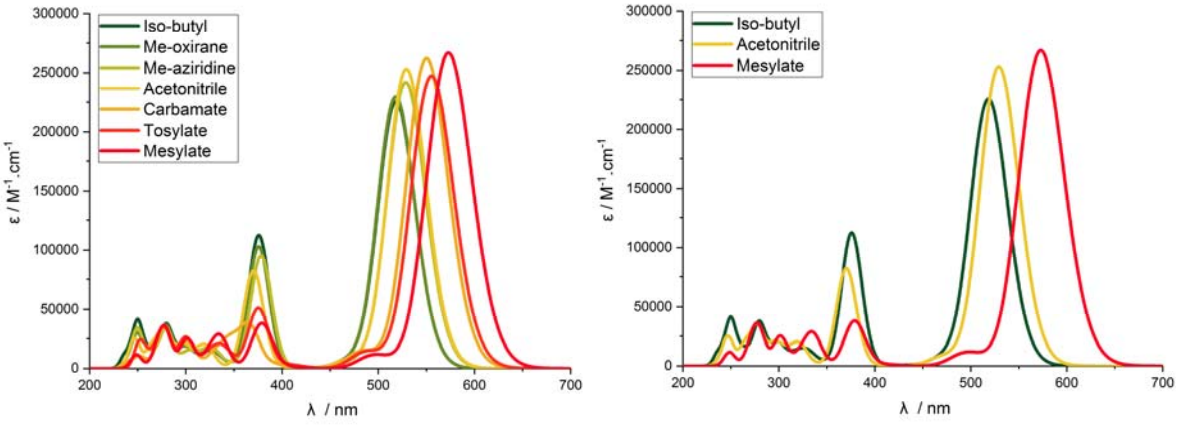

Table S1. Calculated HOMO and LUMO energies for thiophene-isoindigo monomers with **a)** B3LYP and **b)** CAM-B3LYP. The differences (Diff.) to *iso*-butyl substitution and the HOMO from LUMO deviation (Dev.) are included. Units are eV.

**a)**	***iso*-but**	**Me-oxi.**	**Me-azi.**	**AcCN.**	**Mes.**	**Tos.**	**Ket.**	**Carb.**
**HOMO**	-5.6282	-5.8393	-5.7006	-6.0570	-6.0100	-5.8630	-5.9612	-5.8505
**Diff.**	-	-0.2112	-0.0724	-0.4289	-0.3818	-0.2348	-0.3331	-0.2223
**LUMO**	-3.0004	-3.2371	-3.1103	-3.5484	-3.6632	-3.4311	-3.6001	-3.4303
**Diff.**	-	-0.2367	-0.1099	-0.5480	-0.6629	-0.4308	-0.5997	-0.4299
**Dev.**	-	0.0256	0.0376	0.1192	0.2811	0.1959	0.2668	0.2076

**b)**	***iso*-but**	**Me-oxi.**	**Me-azi.**	**AcCN.**	**Mes.**	**Tos.**	**Ket.**	**Carb.**
**HOMO**	-6.8554	-7.0698	-6.9202	-7.2690	-7.2081	-7.0685	-7.1458	-7.0426
**Diff.**	-	-0.2144	-0.0648	-0.4136	-0.3527	-0.2131	-0.2903	-0.1872
**LUMO**	-1.9756	-2.2245	-2.0912	-2.5386	-2.6893	-2.4428	-2.6088	-2.4292
**Diff.**	-	-0.2490	-0.1156	-0.5630	-0.7138	-0.4672	-0.6332	-0.4536
**Dev.**	-	0.0346	0.0509	0.1494	0.3611	0.2542	0.3429	0.2664

Figure S30. HOMO LUMO pairs for **a)** *iso*-butyl, **b)** acetonitrile, and **c)** mesylate substituted thiophene-isoindigo dimers. Isovalue=0.04.

a)

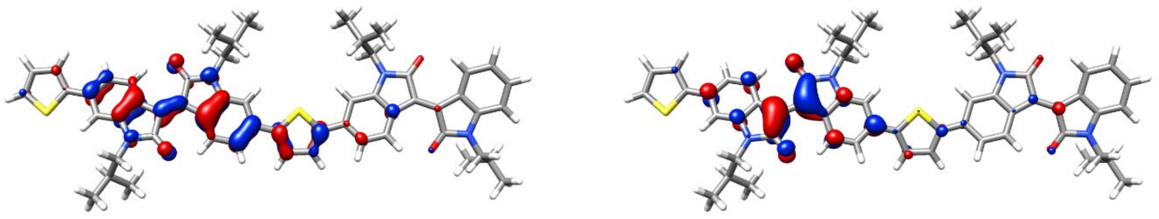

HOMO LUMO

b)

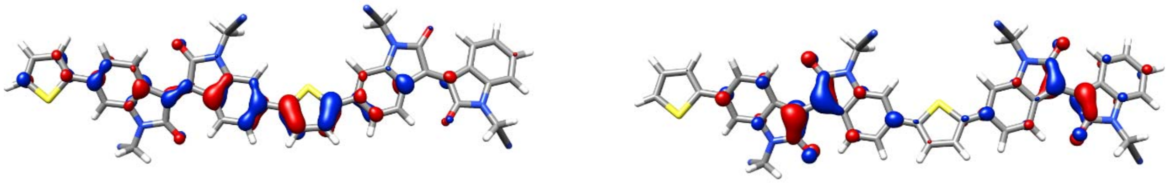

HOMO LUMO

c)

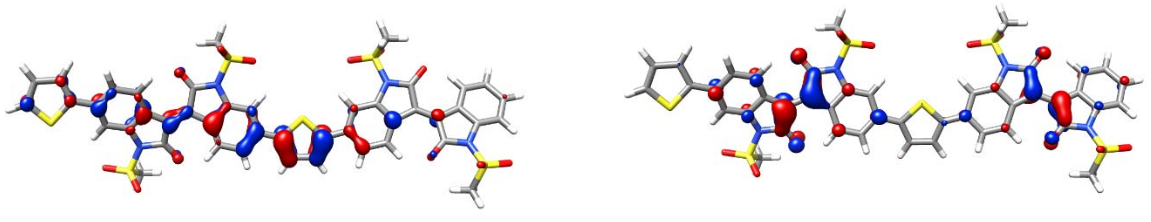

HOMO LUMO

S31: XYZ coordinates for modelled thiophene-isoindigo monomers and dimers. Molecules are identified by the *N*-substitution used and are listed alphabetically, with the monomer followed by the dimer.

Acetonitrile monomer

45

Energy: -1060873.2363986

C	2.23424	-1.84061	0.08594
C	0.88483	-1.51079	0.01640
C	0.49299	-0.17816	-0.19214
C	1.53039	0.78440	-0.29981
C	2.87517	0.47282	-0.24239
C	3.25062	-0.87438	-0.05246
N	0.96908	2.05402	-0.45802
C	-0.41865	1.99389	-0.42115
C	-0.78775	0.53161	-0.24956
C	-3.37423	0.80211	-0.06144
C	-4.41211	-0.16303	-0.13285
C	-5.75410	0.14776	0.02225
C	-6.08634	1.48485	0.26795
C	-5.09052	2.45800	0.36364
C	-3.74195	2.12992	0.20433
C	-2.09492	0.09291	-0.21463
C	-2.47001	-1.36404	-0.39400
N	-3.85658	-1.42708	-0.35182
O	-1.12474	2.97866	-0.57256
C	1.67415	3.29619	-0.67659
O	-1.77115	-2.34425	-0.60713
C	-4.57119	-2.66543	-0.55805
H	2.50759	-2.87317	0.27525
H	0.13891	-2.28359	0.11611
H	3.62997	1.24571	-0.34348
H	-6.52127	-0.61841	-0.02178
H	-7.12998	1.75888	0.39321
H	-5.36212	3.49004	0.56381
H	-2.98684	2.89739	0.26853
H	-3.82467	-3.40561	-0.86432
C	2.43454	3.75280	0.50195
H	0.91369	4.04261	-0.92853
H	2.36184	3.20292	-1.52656
H	-5.30288	-2.55634	-1.36859
C	4.65774	-1.26755	0.00386
S	5.92239	-0.12151	0.41819
C	7.15139	-1.32837	0.24760
C	6.62394	-2.54317	-0.10032
C	5.20923	-2.50894	-0.23790
H	8.18754	-1.07167	0.42299
H	7.22427	-3.43223	-0.25933
H	4.61751	-3.36731	-0.53565
N	3.04765	4.11234	1.41870
C	-5.26951	-3.14664	0.64892
N	-5.83618	-3.52460	1.58792

Acetonitrile dimer

88

Energy: -2121001.3012428

C	9.80622	1.64552	-0.11807
C	8.43239	1.43043	-0.10767
C	7.92094	0.13169	0.05438
C	8.86952	-0.91714	0.18029
C	10.23666	-0.71853	0.19412
C	10.73134	0.59510	0.04617
N	8.19827	-2.13873	0.26816
C	6.82422	-1.96083	0.16138
C	6.58506	-0.46800	0.04676
C	3.98966	-0.50339	-0.19078
C	3.03356	0.54360	-0.10199
C	1.67558	0.35981	-0.27953
C	1.20026	-0.93912	-0.56229
C	2.13458	-1.98898	-0.66683
C	3.49946	-1.78426	-0.49460

C	5.31873	0.08378	-0.00162
C	5.06766	1.56224	0.21331
N	3.69071	1.74753	0.15594
O	6.03624	-2.89405	0.19471
C	8.79307	-3.45136	0.37396
O	5.84322	2.47422	0.45619
C	3.08512	3.04165	0.37199
H	10.17275	2.65444	-0.27451
H	7.75969	2.26573	-0.22171
H	10.91767	-1.54896	0.34913
H	0.99162	1.20173	-0.25010
H	1.78129	-2.99649	-0.85901
H	4.17978	-2.61800	-0.56680
H	3.88173	3.69901	0.73542
C	9.44674	-3.69203	1.67381
H	9.52854	-3.60684	-0.42571
H	7.97912	-4.17113	0.23941
H	2.30979	2.97720	1.14545
C	12.16701	0.86913	0.05989
S	13.34859	-0.37574	-0.31300
C	14.66636	0.72108	-0.07433
C	14.22737	1.97200	0.26799
C	12.81005	2.05646	0.34299
H	15.68419	0.37864	-0.20442
H	14.89260	2.80486	0.46759
H	12.28035	2.95849	0.62792
N	9.97735	-3.88573	2.68695
C	2.49544	3.62055	-0.84981
N	2.01989	4.07491	-1.80532
C	-5.15380	-1.76774	-0.59929
C	-6.50244	-1.47722	-0.42422
C	-6.91070	-0.16606	-0.12969
C	-5.88740	0.80973	-0.00237
C	-4.54500	0.54361	-0.19389
C	-4.15554	-0.77714	-0.50549
N	-6.45754	2.03320	0.35448

C	-7.83247	1.91427	0.52000
C	-8.19038	0.47926	0.17999
C	-10.75816	0.59408	0.63175
C	-11.79693	-0.31061	0.28928
C	-13.12642	-0.10777	0.62439
C	-13.44545	1.05039	1.34242
C	-12.44806	1.95359	1.71224
C	-11.11183	1.73573	1.36771
C	-9.49086	0.01856	0.15652
C	-9.87565	-1.29488	-0.49726
N	-11.25559	-1.40288	-0.39427
O	-8.53760	2.85522	0.84894
C	-5.76306	3.27546	0.60635
O	-9.19103	-2.12406	-1.07825
C	-11.98052	-2.51093	-0.97237
H	-4.86544	-2.79479	-0.79593
H	-7.23515	-2.26286	-0.51900
H	-3.80741	1.33772	-0.14103
H	-13.89300	-0.82888	0.36018
H	-14.47940	1.23686	1.61834
H	-12.70863	2.84381	2.27664
H	-10.35547	2.44976	1.65198
H	-11.25715	-3.07677	-1.56849
C	-5.18723	3.87643	-0.61073
H	-4.96237	3.12665	1.34198
H	-6.50181	3.96101	1.03405
H	-12.76969	-2.14571	-1.64157
C	-2.75407	-1.12355	-0.72098
S	-1.45055	-0.14385	-0.07817
C	-0.22375	-1.20077	-0.74839
C	-0.82267	-2.25125	-1.41768
C	-2.23480	-2.20800	-1.40258
H	-0.25698	-3.01448	-1.93984
H	-2.85705	-2.93460	-1.91253
N	-4.71916	4.34849	-1.56127
C	-12.58666	-3.40182	0.03505

N	-13.08020	-4.09823	0.82060

Aziridine monomer

67

Energy: -1849508.4116658

C	1.76562	2.67006	-0.27840
C	0.66929	1.81431	-0.24273
C	0.80973	0.50726	0.25065
C	2.10532	0.11369	0.67625
C	3.20636	0.94889	0.63604
C	3.04364	2.26256	0.15140
N	2.07716	-1.21835	1.09833
C	0.80844	-1.75397	0.94295
C	-0.07406	-0.65616	0.37333
C	-2.25315	-2.04029	0.01341
C	-3.57740	-1.61910	-0.28052
C	-4.62501	-2.50698	-0.48546
C	-4.34437	-3.87559	-0.40696
C	-3.04993	-4.32337	-0.13904
C	-2.00525	-3.41985	0.07049
C	-1.41387	-0.83488	0.10309
C	-2.36607	0.32011	-0.14528
N	-3.61872	-0.22387	-0.35778
O	0.52512	-2.89262	1.29002
C	3.14647	-1.95698	1.74340
O	-2.17078	1.52974	-0.12036
C	-4.78823	0.60283	-0.60807
C	-5.35501	1.15283	0.69699
H	1.62014	3.68621	-0.62965
H	-0.29814	2.16530	-0.56658
H	4.18478	0.57446	0.91519
H	-5.62889	-2.15966	-0.70924
H	-5.14733	-4.59113	-0.56161
H	-2.84616	-5.38876	-0.08637
H	-1.01271	-3.77904	0.29297
H	-5.52443	0.00762	-1.14935
C	4.14684	-2.56996	0.78374
H	2.66378	-2.76268	2.30519
H	3.66562	-1.29814	2.44844
H	-4.48566	1.43471	-1.24816
H	-4.69475	1.87211	1.17634
C	4.17767	3.18498	0.07801
S	5.62523	2.94599	1.04799
C	6.37629	4.36152	0.38597
C	5.56297	4.98404	-0.52107
C	4.31767	4.31735	-0.69548
H	7.36652	4.65123	0.71119
H	5.84274	5.88612	-1.05442
H	3.55731	4.64863	-1.39355
C	5.51913	-2.94045	1.21640
N	5.30001	-1.72664	0.40170
C	-6.25314	0.33956	1.55978
N	-6.77924	1.52963	0.86012
H	3.71143	-3.18576	-0.00211
H	5.98386	-3.79003	0.72222
H	5.85517	-2.73307	2.22988
H	-6.21894	0.43759	2.64190
H	-6.56915	-0.63041	1.18302
S	5.74302	-1.79939	-1.26832
O	6.22478	-3.14467	-1.61506
O	4.64420	-1.19222	-2.01951
C	7.14218	-0.67000	-1.24324
S	-7.86137	1.28962	-0.45032
O	-8.04207	-0.14392	-0.74374
O	-7.45179	2.20674	-1.51651
C	-9.37031	1.90132	0.31352
H	7.91077	-1.07245	-0.58086
H	6.79685	0.30643	-0.90035
H	7.51601	-0.61223	-2.26803
H	-9.22015	2.94500	0.59109
H	-9.59942	1.28948	1.18777
H	-10.16034	1.80662	-0.43494

Aziridine dimer

132

Energy: -3698273.0214610

C	10.21872	1.41547	-0.83047
C	8.86789	1.26707	-0.53417
C	8.41686	0.12869	0.15404
C	9.39067	-0.84591	0.49807
C	10.73432	-0.71745	0.19980
C	11.17119	0.43881	-0.47833
N	8.77143	-1.92220	1.13894
C	7.40158	-1.72446	1.21210
C	7.10853	-0.38169	0.56785
C	4.51094	-0.43765	0.71021
C	3.54622	0.58871	0.52064
C	2.18169	0.37640	0.61116
C	1.71375	-0.92922	0.87236
C	2.65631	-1.96402	1.03771
C	4.02565	-1.73035	0.96600
C	5.83604	0.14817	0.48768
C	5.56499	1.60356	0.15662
N	4.19554	1.78650	0.21537
O	6.64863	-2.51254	1.76927
C	9.41742	-3.05458	1.77580
O	6.33816	2.52475	-0.07771
C	3.57237	3.07100	-0.06545
C	3.72939	4.03621	1.10683
H	10.54439	2.32177	-1.33012
H	8.16821	2.04148	-0.80756
H	11.42314	-1.52222	0.43071
H	1.48100	1.19828	0.50223
H	2.31011	-2.97883	1.20303
H	4.71731	-2.54552	1.11094
H	2.52595	2.89304	-0.30879
C	9.77578	-4.18093	0.82705
H	8.70577	-3.43485	2.51519
H	10.31687	-2.70976	2.29795
H	4.05314	3.50122	-0.94758
H	4.72651	4.46383	1.17646
C	12.58065	0.62071	-0.82720
S	13.85740	-0.25878	0.00345
C	15.08625	0.49256	-0.96147
C	14.55004	1.37026	-1.86372
C	13.13080	1.44266	-1.78759
H	16.12725	0.24695	-0.79946
H	15.14533	1.94381	-2.56594
H	12.53018	2.06335	-2.44272
C	10.82752	-5.17707	1.15742
N	11.10284	-4.10348	0.17924
C	2.95883	3.86280	2.36694
N	2.68850	5.01987	1.49006
H	8.95299	-4.54207	0.21145
H	10.70039	-6.18285	0.76471
H	11.40313	-5.09060	2.07624
H	3.40292	4.12119	3.32499
H	2.20514	3.07910	2.39099
S	11.12981	-4.54012	-1.49373
O	10.78580	-5.95992	-1.66085
O	10.37599	-3.50898	-2.20705
C	12.88551	-4.32544	-1.81803
S	1.19842	5.05018	0.63377
O	0.43870	3.80217	0.86722
O	1.49891	5.45872	-0.74048
C	0.38163	6.38527	1.50923
H	13.44944	-5.01610	-1.18841
H	13.15522	-3.28936	-1.60814
H	13.03986	-4.56111	-2.87345
H	0.94353	7.30281	1.33156
H	0.36038	6.14079	2.57311
H	-0.63528	6.44454	1.11254
C	-4.69175	-1.55733	0.98708
C	-6.02059	-1.28756	0.67945
C	-6.34514	-0.26126	-0.22200
C	-5.26577	0.49066	-0.75926

C	-3.93924	0.23526	-0.46046
C	-3.62819	-0.82443	0.42104
N	-5.76353	1.48558	-1.60242
C	-7.14917	1.46578	-1.62000
C	-7.59204	0.32179	-0.72465
C	-10.17098	0.66853	-0.87229
C	-11.24151	-0.17853	-0.47937
C	-12.57599	0.18311	-0.60389
C	-12.86208	1.44764	-1.13073
C	-11.83350	2.31242	-1.50759
C	-10.49439	1.93503	-1.38230
C	-8.91499	-0.02084	-0.54020
C	-9.34706	-1.34234	0.06909
N	-10.72854	-1.36164	0.06083
O	-7.80776	2.23340	-2.30818
C	-5.00461	2.42331	-2.41274
O	-8.67862	-2.29149	0.46208
C	-11.48864	-2.49886	0.55407
C	-11.56608	-3.59848	-0.50018
H	-4.47857	-2.35401	1.69134
H	-6.80516	-1.88185	1.12129
H	-3.15584	0.84793	-0.89338
H	-13.37647	-0.48245	-0.29620
H	-13.89880	1.75406	-1.24006
H	-12.07107	3.29311	-1.90892
H	-9.70901	2.60695	-1.69096
H	-12.47943	-2.15251	0.85017
C	-4.69012	3.71996	-1.69538
H	-5.62053	2.65032	-3.28839
H	-4.08264	1.93966	-2.74966
H	-10.98355	-2.88580	1.44201
H	-10.60219	-4.05981	-0.70324
C	-2.24306	-1.16167	0.74062
S	-0.90599	-0.14241	0.23923
C	0.28341	-1.21354	0.95384
C	-0.35033	-2.29282	1.53968
C	-1.75806	-2.26366	1.42128
H	0.18509	-3.07875	2.06027
H	-2.39782	-3.04309	1.81865
C	-3.52656	4.56796	-2.06385
N	-3.47702	3.72909	-0.85031
C	-12.57062	-3.55723	-1.59645
N	-12.66463	-4.59221	-0.54647
H	-5.55539	4.23761	-1.28527
H	-3.61677	5.64322	-1.93114
H	-2.82902	4.25751	-2.83853
H	-12.32914	-3.94401	-2.58327
H	-13.31240	-2.76242	-1.56899
S	-3.57626	4.56075	0.66429
O	-2.70940	5.74726	0.58348
O	-4.98222	4.72100	1.05830
C	-2.80049	3.33667	1.72025
S	-13.94093	-4.47770	0.59570
O	-14.73692	-3.25347	0.39227
O	-13.36336	-4.76231	1.91141
C	-14.92594	-5.88420	0.06115
H	-1.76102	3.22176	1.40728
H	-3.35550	2.40152	1.62927
H	-2.86349	3.72134	2.74116
H	-14.31924	-6.78591	0.14890
H	-15.24608	-5.72266	-0.96967
H	-15.79166	-5.93038	0.72576

Butyl ketone isomer 1

65

H	-6.41961	-5.69064	-0.45072
H	-4.22787	-7.12252	-0.11515
C	-5.40406	-5.32372	-0.38445
C	-4.25080	-6.04290	-0.21559
S	-5.09041	-3.62582	-0.49903
C	-3.09231	-5.22031	-0.17749
C	-3.36134	-3.87335	-0.31525

H	-2.09163	-5.60837	-0.02402
H	-3.89478	-1.22524	0.13835
C	-2.41893	-2.75573	-0.32023
C	-2.85702	-1.43904	-0.06762
O	-4.40051	0.83467	0.52931
H	-0.71182	-3.98167	-0.82740
H	-7.17637	5.22553	0.51671
H	-6.84659	5.08155	2.24883
H	-5.62934	3.17730	0.06234
C	-1.05255	-2.98047	-0.58563
H	-5.30175	3.03491	1.77487
C	-6.47971	5.51798	1.31184
C	-3.42976	1.55753	0.40055
C	-4.95414	3.52165	0.85537
C	-1.92857	-0.41054	-0.06821
H	-6.52569	6.60839	1.41129
C	-5.05609	5.04522	1.00160
N	-2.16334	0.98073	0.10695
C	-0.12929	-1.94689	-0.56647
C	-3.52529	3.06560	0.54476
H	-4.70366	5.52618	0.07790
C	-0.54433	-0.63252	-0.28816
H	-4.37640	5.38303	1.79687
H	-3.15014	3.53658	-0.37156
H	0.91095	-2.15544	-0.76041
H	-2.82618	3.39472	1.32245
C	-0.94132	1.67840	-0.06304
C	0.13880	0.65212	-0.29298
O	-0.82623	2.88525	0.03718
O	2.47897	-1.15883	0.21998
C	1.47566	0.99600	-0.38802
C	2.57679	0.01461	-0.09139
H	0.63093	3.68056	-1.25617
H	4.76374	-1.88606	-0.27650
H	4.58763	-1.45879	1.39107
C	2.14793	2.26113	-0.67254
H	6.36602	-3.78261	0.36467
H	6.18923	-3.34946	2.05692
C	1.68824	3.50498	-1.13600
C	5.21373	-1.27016	0.51111
N	3.80355	0.72003	-0.15316
C	6.79410	-3.15529	1.15968
H	8.30622	-4.63739	1.69692
C	3.54672	2.06404	-0.54224
C	5.09431	0.19305	0.12404
C	6.66763	-1.67752	0.76830
C	8.24305	-3.57907	1.41939
C	2.60125	4.51700	-1.42031
H	7.26850	-1.47648	-0.12722
H	7.09037	-1.04523	1.55870
H	2.23822	5.47821	-1.77212
H	8.86522	-3.42981	0.52848
H	8.68702	-2.99341	2.23359
C	4.46479	3.07247	-0.81587
O	6.06707	0.92070	0.04619
C	3.97108	4.30638	-1.25011
H	5.52298	2.89872	-0.69861
H	4.67408	5.10687	-1.46361

Butyl ketone isomer 2

65

H	-7.20502	-4.55610	-0.34421
H	-5.35697	-6.31015	0.34280
C	-6.16009	-4.36020	-0.14438
C	-5.18038	-5.24858	0.20717
S	-5.54122	-2.74666	-0.27152
C	-3.91144	-4.62908	0.38434
C	-3.92210	-3.26760	0.16870
H	-3.02125	-5.16646	0.69157
H	-4.04552	-0.57740	0.63822
C	-2.80899	-2.32188	0.26600
C	-3.03495	-0.95265	0.51418

H	-1.29786	-3.81055	-0.12663
H	-7.78147	3.71712	-1.09541
H	-6.91342	5.25430	-0.96601
H	-5.85051	2.53107	0.21228
C	-1.48237	-2.76787	0.11007
H	-3.86773	1.64930	1.64225
H	-2.81574	3.04928	1.40289
H	-4.98560	4.04308	0.34191
C	-7.01214	4.34154	-1.56668
C	-3.14077	2.10255	0.95629
C	-5.12635	3.17174	-0.31614
C	-1.94786	-0.10305	0.60857
H	-7.38249	4.63388	-2.55537
C	-5.67859	3.59721	-1.67855
N	-1.97372	1.27062	0.84065
C	-0.39685	-1.90343	0.21907
C	-3.81003	2.41282	-0.39580
H	-5.79226	2.70808	-2.30892
C	-0.60347	-0.54151	0.48654
H	-4.93623	4.22715	-2.18274
H	0.60644	-2.28238	0.10228
O	-3.29959	2.07535	-1.44232
C	-0.69490	1.80305	0.78787
C	0.26101	0.63809	0.59363
O	-0.47083	2.99457	0.94573
O	2.35431	-1.58250	0.89064
C	1.63114	0.78302	0.59210
C	2.58300	-0.38784	0.75570
H	1.27731	3.70835	0.17220
O	5.22171	-0.60859	-1.47411
C	2.49933	1.96642	0.50515
H	7.73923	-1.23257	-2.30138
H	6.84305	-2.73308	-2.27336
C	2.28364	3.33157	0.26697
C	5.70792	-0.98575	-0.42955
N	3.86271	0.13870	0.81242
C	7.58623	-2.14817	-1.71884
H	9.28721	-3.18578	-2.60033
C	3.84185	1.51892	0.61402
C	5.02660	-0.69793	0.92111
C	7.00626	-1.77622	-0.35230
C	8.89758	-2.93118	-1.60862
C	3.37365	4.20065	0.17252
H	5.75003	-0.25726	1.61923
H	7.72959	-1.18068	0.22741
H	6.82713	-2.67562	0.25693
H	4.69695	-1.65125	1.34993
H	3.19411	5.25649	-0.00733
H	9.66922	-2.35030	-1.08802
H	8.75997	-3.86857	-1.05542
C	4.93140	2.37238	0.52342
C	4.68137	3.73136	0.30535
H	5.94771	1.99938	0.60051
H	5.51768	4.42119	0.23059

Butyl ketone isomer 3

65

H	7.30134	4.63147	-0.47602
H	5.44353	6.27500	0.42538
C	6.24832	4.41997	-0.34709
C	5.26362	5.25236	0.11188
S	5.62301	2.85707	-0.75629
C	3.98469	4.62939	0.14192
C	3.99310	3.32111	-0.29329
H	3.08839	5.12194	0.50199
H	4.08111	0.58802	-0.28772
C	2.87022	2.38737	-0.39213
C	3.07841	0.99243	-0.37956
H	1.38046	3.93982	-0.55124
H	6.55638	-5.28574	1.95433
O	5.52927	-3.41758	0.24388
H	7.28885	-3.75906	2.44340

C	1.55182	2.86962	-0.50072
H	2.92809	-3.03221	-0.91060
H	4.00506	-1.63865	-0.90300
C	6.48348	-4.46384	2.67212
C	3.16222	-2.10366	-0.38643
C	4.80241	-3.22550	1.20195
C	1.98233	0.15153	-0.46591
H	6.64996	-4.86375	3.67758
C	5.11870	-3.78356	2.58359
N	1.99669	-1.24200	-0.50368
C	0.45672	2.01273	-0.56932
C	3.52336	-2.40471	1.07328
H	4.31230	-4.48586	2.84414
C	0.64402	0.62221	-0.54235
H	5.03015	-2.96625	3.31422
H	2.69509	-2.93122	1.56461
H	-0.54072	2.41874	-0.63498
H	3.66038	-1.46959	1.63619
C	0.71192	-1.74204	-0.60988
C	-0.23202	-0.54965	-0.65807
O	0.46694	-2.94143	-0.60273
O	-2.33237	1.62310	-0.13790
C	-1.60041	-0.67553	-0.76091
C	-2.55862	0.47560	-0.50344
H	-1.23998	-3.53705	-1.51015
H	-4.79860	1.07592	1.66390
H	-5.72277	-0.34819	1.22606
C	-2.46767	-1.82101	-1.08074
H	-6.57374	2.30619	3.08175
H	-7.25457	0.69990	3.11720
C	-2.24643	-3.15113	-1.46644
C	-5.55127	0.70471	0.95687
N	-3.83849	-0.00869	-0.69366
C	-7.32122	1.66375	2.59169
H	-8.98843	2.41407	3.76056
C	-3.81104	-1.36084	-1.03680
C	-5.02183	0.80781	-0.47887
C	-6.86277	1.45786	1.15382
C	-8.71926	2.26773	2.70952
C	-3.33202	-3.97783	-1.76918
H	-4.74355	1.84029	-0.69940
H	-5.79430	0.51546	-1.19371
H	-3.14702	-5.00768	-2.06003
H	-8.77039	3.23373	2.19940
H	-9.46851	1.61616	2.24922
O	-7.50641	1.86657	0.20407
C	-4.89701	-2.17536	-1.32770
C	-4.64137	-3.50126	-1.69480
H	-5.91318	-1.79702	-1.28210
H	-5.47474	-4.15938	-1.92562

Butyl ketone isomer 4

65

H	7.50253	4.37413	-0.36417
H	5.70130	6.05273	0.58517
C	6.43996	4.20136	-0.25804
C	5.48391	5.05306	0.22481
S	5.75614	2.68407	-0.74068
C	4.18002	4.48277	0.21346
C	4.14047	3.19512	-0.27681
H	3.29985	4.99519	0.58538
H	4.11204	0.46208	-0.34527
C	2.98186	2.31347	-0.42899
C	3.13086	0.91163	-0.45535
H	1.56358	3.93239	-0.57310
H	5.84396	-5.78243	2.20408
H	7.09919	-4.62252	1.77135
C	1.68823	2.85470	-0.55217
H	2.77724	-3.10171	-1.09025
H	3.92208	-1.75400	-1.17393
H	5.55646	-3.06357	0.32223
H	4.44183	-4.40490	0.42590

C	6.26418	-4.80106	2.45965
C	3.09274	-2.17935	-0.59476
C	4.70095	-3.44698	0.90110
C	2.00297	0.12105	-0.59302
H	6.62981	-4.82511	3.48815
C	5.19738	-3.72805	2.31742
N	1.96186	-1.26881	-0.68102
C	0.56006	2.04757	-0.67217
C	3.52495	-2.47114	0.84871
C	0.68777	0.65010	-0.68559
O	4.76166	-3.12684	3.28015
H	2.67439	-2.88877	1.39817
H	-0.41714	2.49752	-0.74972
H	3.79643	-1.54258	1.36198
C	0.66234	-1.71039	-0.85742
C	-0.23337	-0.47960	-0.86047
O	0.37313	-2.89618	-0.94461
O	-2.24367	1.76800	-0.30747
C	-1.60575	-0.54782	-0.97005
C	-2.51833	0.63087	-0.67017
H	-1.35971	-3.38282	-1.85397
O	-7.28545	0.55747	3.02323
H	-4.87173	0.98192	1.55725
H	-5.95245	-0.21787	0.87576
C	-2.51814	-1.64811	-1.32161
C	-2.35108	-2.96633	-1.77069
C	-5.62172	0.82113	0.77507
N	-3.81908	0.19063	-0.83228
C	-7.54028	1.51360	2.31667
H	-9.15675	2.17700	3.57146
C	-3.84372	-1.14581	-1.22965
C	-4.97023	1.04671	-0.59619
C	-6.80874	1.75851	0.99898
C	-8.60461	2.52922	2.69784
C	-3.47010	-3.74138	-2.08776
H	-4.60473	2.07398	-0.67899
H	-6.49490	2.81216	0.96180
H	-7.55011	1.65308	0.19034
H	-5.69469	0.88189	-1.40409
H	-3.32634	-4.76294	-2.42721
H	-8.13258	3.49144	2.93547
H	-9.29662	2.71007	1.86645
C	-4.96238	-1.90942	-1.53424
C	-4.76053	-3.22517	-1.96483
H	-5.96359	-1.50090	-1.44399
H	-5.62057	-3.84448	-2.20507

***iso*-Butyl monomer**

61

Energy: -1093144.8626662

C	2.12788	-2.17768	-0.51394
C	0.83559	-1.66014	-0.48996
C	0.61022	-0.33883	-0.07407
C	1.74993	0.43015	0.28455
C	3.03775	-0.07832	0.28111
C	3.24308	-1.41473	-0.11895
N	1.36273	1.72380	0.62943
C	-0.00779	1.87247	0.49997
C	-0.56359	0.53544	0.02966
C	-3.05983	1.22541	-0.25729
C	-4.22091	0.42682	-0.43578
C	-5.48815	0.96854	-0.60754
C	-5.61031	2.36213	-0.61523
C	-4.48611	3.17434	-0.46105
C	-3.21613	2.61902	-0.28292
C	-1.90837	0.31279	-0.17565
C	-2.50382	-1.07762	-0.33186
N	-3.87446	-0.92421	-0.44441
O	-0.59432	2.90800	0.78355
C	2.23139	2.78466	1.11885
O	-1.95477	-2.17221	-0.32447
C	-4.78600	-2.04511	-0.61823
C	-5.59758	-2.41373	0.64102

H	2.27695	-3.19302	-0.86642
H	0.00225	-2.27900	-0.78329
H	3.87466	0.53408	0.59938
H	-6.35781	0.33306	-0.73783
H	-6.59214	2.80960	-0.74490
H	-4.59368	4.25498	-0.47261
H	-2.35771	3.25828	-0.14931
H	-4.16708	-2.89418	-0.92304
C	3.04803	3.50575	0.02615
C	3.99464	4.51609	0.69015
C	2.14459	4.18366	-1.01194
C	-6.59160	-3.53059	0.29144
C	-4.68950	-2.81710	1.80976
H	2.90560	2.35314	1.86935
H	1.57771	3.50005	1.62681
H	-5.46528	-1.80821	-1.44724
H	-6.17242	-1.52701	0.94474
H	3.65994	2.75387	-0.49240
H	4.66580	4.03108	1.40945
H	3.42986	5.28893	1.22726
H	4.61528	5.01969	-0.05911
H	1.50074	4.93637	-0.54141
H	1.49609	3.46001	-1.51631
H	2.74710	4.68314	-1.77935
H	-7.20948	-3.78867	1.15854
H	-6.06482	-4.44069	-0.02318
H	-7.26446	-3.23511	-0.52298
H	-4.08112	-3.69210	1.55149
H	-5.28729	-3.06726	2.69382
H	-4.00610	-2.00808	2.08693
C	4.58299	-2.00429	-0.12961
S	6.02696	-1.01242	-0.26391
C	7.06058	-2.40150	-0.19548
C	6.34024	-3.55956	-0.08286
C	4.93548	-3.33435	-0.04607
H	8.13419	-2.27999	-0.24797
H	6.79486	-4.54235	-0.02037
H	4.20553	-4.12772	0.06940

***iso*-Butyl dimer**

120

Energy: -2185544.0033456

C	-9.92608	1.60088	-0.86111
C	-8.55617	1.39101	-0.73185
C	-8.07388	0.23078	-0.10554
C	-9.03942	-0.70643	0.35266
C	-10.40424	-0.50079	0.24496
C	-10.87072	0.68016	-0.36843
N	-8.40145	-1.81387	0.90802
C	-7.02622	-1.67295	0.82804
C	-6.74857	-0.33580	0.15898
C	-4.15439	-0.49324	0.03175
C	-3.17861	0.49927	-0.25718
C	-1.82228	0.23072	-0.32412
C	-1.37513	-1.08855	-0.10042
C	-2.33155	-2.08522	0.17757
C	-3.69264	-1.80268	0.23929
C	-5.47179	0.14336	-0.05650
C	-5.17938	1.58745	-0.42928
N	-3.80194	1.72185	-0.49712
O	-6.24484	-2.49770	1.28331
C	-9.04532	-2.95262	1.54700
O	-5.94760	2.52296	-0.60988
C	-3.14397	2.97432	-0.83890
C	-2.49176	3.70346	0.35406
H	-10.27123	2.49231	-1.37450
H	-7.86137	2.12626	-1.10625
H	-11.10555	-1.22642	0.64293
H	-1.11563	1.01431	-0.57580
H	-2.00043	-3.09801	0.38205
H	-4.39404	-2.58802	0.47378
H	-3.91568	3.60843	-1.28517
C	-9.63004	-3.99486	0.57090

C	-10.37168	-5.07434	1.37245
C	-8.54920	-4.60855	-0.32823
C	-1.74550	4.94298	-0.16007
C	-3.52010	4.07478	1.43015
H	-9.83446	-2.57081	2.20692
H	-8.28006	-3.41947	2.17433
H	-2.39144	2.76655	-1.61047
H	-1.75486	3.02347	0.80515
H	-10.36084	-3.48481	-0.07284
H	-11.16848	-4.64428	1.99152
H	-9.68363	-5.61167	2.03776
H	-10.82921	-5.81149	0.70353
H	-7.78512	-5.12080	0.26871
H	-8.04472	-3.84595	-0.93034
H	-8.98972	-5.33929	-1.01624
H	-1.24009	5.46251	0.66142
H	-2.43977	5.65451	-0.62530
H	-0.98652	4.67924	-0.90687
H	-4.28876	4.74411	1.02567
H	-3.03260	4.58434	2.26924
H	-4.02666	3.18906	1.82672
C	-12.30325	0.95092	-0.49772
S	-13.49184	-0.34306	-0.48999
C	-14.80153	0.77732	-0.66837
C	-14.35340	2.06858	-0.73642
C	-12.93700	2.16695	-0.64029
H	-15.81982	0.41561	-0.71759
H	-15.01100	2.92439	-0.84416
H	-12.40155	3.10974	-0.64541
C	4.97760	-1.95880	0.18343
C	6.33018	-1.63433	0.22576
C	6.74863	-0.31310	0.00311
C	5.73583	0.65598	-0.23201
C	4.38879	0.34326	-0.29255
C	3.98774	-0.99409	-0.08918
N	6.30995	1.91597	-0.38823

C	7.68609	1.84469	-0.24804
C	8.03675	0.38789	0.01815
C	10.60204	0.65941	0.38314
C	11.63366	-0.31605	0.43137
C	12.96315	0.00585	0.67134
C	13.28417	1.35062	0.88498
C	12.29069	2.33043	0.86246
C	10.95665	1.99672	0.61429
C	9.33280	-0.05487	0.17710
C	9.71944	-1.52446	0.11993
N	11.09618	-1.58913	0.24229
O	8.41918	2.81486	-0.38171
C	5.61069	3.15127	-0.71134
O	9.01797	-2.51448	-0.04563
C	11.83438	-2.84327	0.23863
C	12.60126	-3.13286	-1.06838
H	4.68131	-2.98261	0.38626
H	7.06203	-2.40245	0.42039
H	3.65329	1.10987	-0.51169
H	13.73063	-0.76051	0.69882
H	14.31831	1.62763	1.07167
H	12.55257	3.37042	1.03362
H	10.20091	2.76575	0.58425
H	11.09547	-3.62986	0.41789
C	4.89267	3.81745	0.48096
C	4.11307	5.04118	-0.02152
C	5.87044	4.19774	1.60018
C	13.41878	-4.42144	-0.89848
C	11.66045	-3.22115	-2.27684
H	4.89134	2.93574	-1.51145
H	6.36794	3.83005	-1.11503
H	12.52933	-2.83363	1.08817
H	13.30220	-2.30339	-1.24052
H	4.17046	3.09590	0.88900
H	3.38904	4.76970	-0.79954
H	4.79208	5.79299	-0.44391

H	3.56170	5.51571	0.79762
H	6.62431	4.90780	1.23967
H	6.39853	3.32126	1.98934
H	5.33642	4.66369	2.43629
H	14.00473	-4.63353	-1.79954
H	12.76193	-5.28244	-0.71966
H	14.11607	-4.35171	-0.05453
H	10.92862	-4.02752	-2.14773
H	12.22787	-3.42026	-3.19328
H	11.10488	-2.28945	-2.42410
C	2.58043	-1.38012	-0.15315
S	1.29079	-0.21225	0.07217
C	0.04491	-1.42548	-0.15829
C	0.62825	-2.65790	-0.37892
C	2.04230	-2.63250	-0.37639
H	0.05035	-3.55384	-0.57585
H	2.65260	-3.50701	-0.57181

Carbamate monomer

49

Energy: -1181765.9988141

C	-2.15519	-2.07496	-0.38814
C	-0.85412	-1.60004	-0.32987
C	-0.60686	-0.24140	-0.06284
C	-1.73056	0.60654	0.10794
C	-3.03446	0.14079	0.07016
C	-3.26229	-1.22882	-0.17308
N	-1.29093	1.94477	0.27545
C	0.13243	1.99098	0.18097
C	0.60110	0.57194	-0.03573
C	3.13587	1.04761	-0.37219
C	4.27382	0.20968	-0.25993
C	5.56192	0.66287	-0.52052
C	5.71715	1.99197	-0.92375
C	4.61228	2.83279	-1.07434
C	3.32694	2.37168	-0.80239
C	1.94284	0.24520	-0.10807
C	2.44046	-1.14874	0.17753
N	3.86331	-1.10281	0.09162
O	0.79893	2.98981	0.33415
C	-2.15672	3.02773	0.50521
O	1.80181	-2.12599	0.50270
C	4.75757	-2.16095	0.32427
H	-2.31932	-3.12208	-0.61957
H	-0.03046	-2.27934	-0.48249
H	-3.85408	0.82237	0.24534
H	6.40925	0.00250	-0.41254
H	4.75095	3.85984	-1.39901
H	2.48244	3.03466	-0.90427
O	-1.51415	4.19841	0.49002
O	-3.35094	2.89967	0.68483
O	5.96381	-2.02165	0.34763
C	-4.62037	-1.77003	-0.20788
S	-6.01928	-0.74116	-0.47093
C	-7.10191	-2.08754	-0.36622
C	-6.43183	-3.26088	-0.14392
C	-5.02440	-3.08081	-0.05525
H	-8.16699	-1.93383	-0.47725
H	-6.92488	-4.22128	-0.03991
H	-4.33171	-3.89061	0.14456
O	4.12617	-3.32499	0.50289
H	6.71681	2.36655	-1.12618
C	-2.34845	5.34370	0.73755
C	4.99272	-4.44245	0.76415
H	-1.67083	6.19650	0.70495
H	-2.82587	5.26599	1.71737
H	-3.11900	5.43088	-0.03249
H	5.58166	-4.26871	1.66808
H	5.66823	-4.61013	-0.07845
H	4.32301	-5.29177	0.89755

Carbamate dimer

96

Energy: -2362781.8149462

C	-4.34415	-0.12799	-0.21630
C	-5.69212	-0.43205	-0.09709
C	-6.63845	0.59699	0.05270
C	-6.15399	1.92969	0.10736
C	-4.81232	2.24573	-0.00634
C	-3.87907	1.20201	-0.18605
N	-7.23501	2.81486	0.35285
C	-8.44145	2.06417	0.48560
C	-8.07453	0.61229	0.29508
C	-10.38730	-0.45162	0.83046
C	-10.93240	-1.72610	0.52521
C	-12.19060	-2.11657	0.96914
C	-12.92631	-1.21516	1.74256
C	-12.40638	0.03722	2.07537
C	-11.14551	0.42236	1.62775
C	-9.02126	-0.39492	0.31213
C	-8.77618	-1.73256	-0.33759
N	-9.97479	-2.48651	-0.19203
O	-9.53964	2.55024	0.63858
C	-7.09992	4.21223	0.41372
O	-7.81018	-2.10757	-0.96459
C	-10.08663	-3.79003	-0.71108
H	-3.63994	-0.94126	-0.36391
H	-6.01435	-1.46014	-0.14863
H	-4.49357	3.27411	0.06070
H	-12.58970	-3.08934	0.72266
H	-12.98774	0.72311	2.68427
H	-10.75809	1.39716	1.87869
O	-8.22773	4.80999	0.80521
O	-6.06667	4.79431	0.15016
O	-9.15468	-4.49977	-0.99638
C	-2.46372	1.52415	-0.33295
S	-1.21415	0.31597	-0.10062
C	0.06587	1.46192	-0.44878
C	-0.47687	2.70564	-0.71407
C	-1.88750	2.74064	-0.64938

H	0.12565	3.56522	-0.98485
H	-2.46939	3.63016	-0.86255
O	-11.38419	-4.13462	-0.85993
H	-13.91441	-1.50396	2.09016
C	-8.15671	6.24535	0.86827
C	-11.58964	-5.46464	-1.37094
H	-7.91155	6.66066	-0.11224
H	-7.40140	6.56066	1.59238
H	-9.15064	6.56173	1.18359
H	-11.13879	-6.20529	-0.70596
H	-11.14992	-5.56063	-2.36623
H	-12.67159	-5.59046	-1.41366
C	9.70271	-2.02447	0.94279
C	8.38940	-1.73152	0.60572
C	8.06127	-0.47449	0.06723
C	9.11135	0.46859	-0.08084
C	10.42360	0.18988	0.25641
C	10.73955	-1.08678	0.76761
N	8.57782	1.69798	-0.54729
C	7.16258	1.57711	-0.68612
C	6.80505	0.16706	-0.29007
C	4.21876	0.36704	-0.42885
C	3.19515	-0.61165	-0.53791
C	1.85013	-0.28171	-0.54553
C	1.47227	1.07145	-0.43089
C	2.47947	2.04861	-0.29225
C	3.82354	1.71009	-0.29330
C	5.50660	-0.30926	-0.35739
C	5.19811	-1.78133	-0.44188
N	3.78273	-1.89966	-0.56549
O	6.43787	2.44531	-1.12053
C	9.36169	2.82436	-0.84663
O	5.96362	-2.71729	-0.50010
C	3.15991	-3.15853	-0.67067
H	9.93194	-3.01455	1.32536
H	7.62544	-2.48252	0.73202

H	11.18260	0.94823	0.14406
H	1.10183	-1.05164	-0.66319
H	2.20215	3.08937	-0.16283
H	4.56842	2.48422	-0.19692
O	8.61607	3.89850	-1.12005
O	10.57647	2.81289	-0.84164
O	3.66199	-4.20346	-0.34057
C	12.12717	-1.40234	1.10191
S	12.53962	-2.72915	2.17733
C	14.23330	-2.40686	2.02021
C	14.47125	-1.34126	1.19388
C	13.27727	-0.77073	0.67422
H	14.95091	-3.01990	2.54890
H	15.46543	-0.97771	0.95798
H	13.26695	0.06545	-0.01584
O	1.92610	-3.03698	-1.20562
C	9.36522	5.07979	-1.45396
C	1.20647	-4.27821	-1.33038
H	8.61262	5.84066	-1.65944
H	9.98734	4.90364	-2.33488
H	10.00179	5.37998	-0.61788
H	1.08332	-4.74960	-0.35239
H	1.74179	-4.96076	-1.99415
H	0.23973	-4.00638	-1.75399

Ketone monomer

47

Energy: -1087364.4941888

C	-2.28906	-1.77946	-0.41051
C	-0.94101	-1.46121	-0.35827
C	-0.53519	-0.14728	-0.06413
C	-1.54899	0.82510	0.13640
C	-2.89860	0.51478	0.10386
C	-3.28725	-0.81440	-0.16402
N	-0.95043	2.09855	0.33304
C	0.45537	1.97404	0.19721
C	0.75996	0.51571	-0.03288
C	3.33821	0.68897	-0.34094
C	4.36898	-0.27369	-0.18948
C	5.70642	0.02854	-0.42118
C	6.02048	1.32664	-0.83484
C	5.02281	2.28493	-1.02558
C	3.68691	1.97600	-0.78301
C	2.05538	0.03352	-0.09717
C	2.38880	-1.40380	0.19612
N	3.80030	-1.52510	0.17308
O	1.23408	2.90002	0.32103
C	-1.66023	3.29475	0.61276
O	1.63201	-2.31555	0.47691
C	4.54319	-2.69858	0.45985
H	-2.57697	-2.79427	-0.66386
H	-0.20354	-2.22717	-0.53876
H	-3.63055	1.28478	0.29586
H	6.46976	-0.72227	-0.28813
H	5.28549	3.28387	-1.36114
H	2.92467	2.72668	-0.91917
C	-0.87754	4.57563	0.78734
O	-2.87316	3.27066	0.70809
O	5.75902	-2.67241	0.41273
C	-4.69924	-1.19137	-0.19388
S	-5.97098	0.00270	-0.39764
C	-7.20204	-1.21064	-0.31894
C	-6.67193	-2.46184	-0.14745
C	-5.25245	-2.45089	-0.07770
H	-8.24308	-0.92964	-0.40635
H	-7.27318	-3.36083	-0.06694
H	-4.65766	-3.34310	0.08255
C	3.79189	-3.95938	0.82115
H	-0.15917	4.49961	1.60742
H	-1.60799	5.35971	0.99298
H	-0.30032	4.81998	-0.10801
H	3.11666	-4.26926	0.01952
H	3.17209	-3.81790	1.71008

H	4.54489	-4.72840	1.00090
H	7.06077	1.58259	-1.01598

Ketone dimer

92

Energy: -2173447.1068198

H	-14.32227	1.25141	1.81808
H	-13.84699	-0.80196	0.54889
C	-13.29991	1.03564	1.52059
H	-12.98853	-2.85276	1.22509
C	-13.03483	-0.13559	0.80436
H	-14.00892	-2.83702	-0.23225
H	-12.48985	2.81906	2.41841
C	-13.05639	-3.18215	0.18599
C	-12.27261	1.91956	1.85063
C	-11.89752	-2.70946	-0.67430
H	-13.05837	-4.27344	0.13775
C	-11.72193	-0.40078	0.43021
O	-11.52166	-3.33797	-1.63546
N	-11.21830	-1.51838	-0.27157
C	-10.96536	1.67380	1.43461
C	-10.67057	0.51782	0.69396
H	-10.18567	2.38363	1.66069
C	-9.81799	-1.36398	-0.46152
C	-9.43115	-0.03875	0.15219
O	-9.11455	-2.17357	-1.02535
O	-8.58950	2.84998	0.44394
C	-8.14296	0.45591	0.08627
C	-7.82842	1.92659	0.26182
H	-7.18415	-2.30948	-0.34314
C	-6.86339	-0.18088	-0.21024
C	-6.45133	-1.51834	-0.34262
O	-6.23578	4.27743	0.66057
N	-6.43446	2.08259	0.02367
H	-4.77787	4.57729	-1.40749
C	-5.85495	0.81965	-0.20838
C	-5.82075	3.37158	-0.02270
C	-4.68255	3.56398	-1.00980
C	-5.10615	-1.82840	-0.48224
H	-4.81643	-2.87065	-0.56149
H	-3.71767	3.51007	-0.49261
C	-4.50810	0.51493	-0.32482
H	-4.68705	2.84006	-1.82732
C	-4.11256	-0.82911	-0.48375
H	-3.75639	1.29232	-0.29741
H	-2.78327	-3.14178	-1.58141
C	-2.70595	-1.19049	-0.63490
C	-2.17190	-2.34839	-1.16676
S	-1.41343	-0.12615	-0.11136
C	-0.75877	-2.38089	-1.16727
H	-0.18485	-3.20224	-1.58097
C	-0.17176	-1.24851	-0.63624
H	0.98711	1.18592	-0.35909
C	1.24977	-0.95052	-0.48469
C	1.70416	0.37731	-0.34286
H	1.03310	3.35590	-0.46510
H	1.86371	-3.02086	-0.56966
H	2.04289	4.41089	-1.44990
C	2.19715	-1.99267	-0.47956
C	1.96002	3.39826	-1.04864
H	1.89585	2.67581	-1.86528
C	3.06090	0.62344	-0.20453
C	3.16384	3.18616	-0.14714
O	3.68077	4.10116	0.44861
C	3.55094	-1.74304	-0.30410
N	3.71208	1.86794	-0.07420
C	4.01586	-0.42752	-0.13654
H	4.24288	-2.56914	-0.26176
C	5.11339	1.65423	0.06195
C	5.33875	0.16226	0.03034
O	5.92558	2.54473	0.18207
O	5.93859	-2.66870	0.89275
C	6.59157	-0.40074	0.19798

C	6.78017	-1.84173	0.61915
H	7.78247	2.18277	-0.61421
C	7.93611	0.15576	0.10729
N	8.17720	-2.04483	0.78779
O	8.14458	-4.31852	1.08921
C	8.45340	1.38131	-0.34875
C	8.70097	-3.26823	1.30719
C	8.86618	-0.86662	0.43684
H	10.07368	-2.19359	2.64248
C	9.93317	-3.17747	2.19015
H	9.81913	-3.93452	2.96980
C	9.82415	1.56985	-0.45274
H	10.19449	2.51785	-0.82800
C	10.23527	-0.69109	0.31452
H	10.83167	-3.43634	1.61827
C	10.73908	0.55130	-0.12082
H	10.92369	-1.49142	0.55027
C	12.17821	0.78359	-0.23881
H	12.35291	2.94077	-0.11483
C	12.85304	1.98652	-0.23639
S	13.32002	-0.54054	-0.41253
C	14.26321	1.85318	-0.36538
C	14.66522	0.54882	-0.46807
H	14.94977	2.69261	-0.37473
H	15.66906	0.16163	-0.58013

Mesylate monomer

49

Energy: -1633575.5582775

C	-2.15856	-2.20500	-0.41259
C	-0.88034	-1.67023	-0.37084
C	-0.69374	-0.30255	-0.10023
C	-1.85647	0.49018	0.09266
C	-3.13632	-0.03307	0.06933
C	-3.30162	-1.41233	-0.17909
N	-1.45552	1.83827	0.29147
C	-0.05981	1.95610	0.13206
C	0.47677	0.56267	-0.08051
C	2.99151	1.14657	-0.39870
C	4.16914	0.36725	-0.24988
C	5.43786	0.87686	-0.49005
C	5.53457	2.20846	-0.90687
C	4.39430	2.99337	-1.09029
C	3.12651	2.47325	-0.84115
C	1.83163	0.29397	-0.14471
C	2.39235	-1.06997	0.15524
N	3.79567	-0.94741	0.14070
O	0.53703	3.01128	0.25153
S	-2.47164	3.25651	0.31812
O	1.81567	-2.10106	0.45873
S	4.81561	-2.35813	0.22739
H	-2.27696	-3.25639	-0.65129
H	-0.02698	-2.30746	-0.54447
H	-3.98926	0.60640	0.24839
H	6.31750	0.26122	-0.36885
H	4.49045	4.02063	-1.42864
H	2.25134	3.08995	-0.97547
O	6.18667	-1.85571	0.28081
C	-1.86643	4.10006	1.78528
O	-3.82495	2.77075	0.57809
O	-2.17585	4.05607	-0.86301
O	4.39951	-3.29198	-0.81057
C	-4.63249	-2.01552	-0.20226
S	-6.08266	-1.05044	-0.42662
C	-7.09713	-2.44805	-0.32542
C	-6.36900	-3.59225	-0.13298
C	-4.97107	-3.34700	-0.06421
H	-8.16987	-2.34374	-0.41888
H	-6.81453	-4.57640	-0.03788
H	-4.23791	-4.12647	0.10992
C	4.37672	-3.01678	1.84126
H	6.51850	2.62765	-1.09777
H	-2.05319	3.46322	2.65135

H	-2.44371	5.02536	1.85586
H	-0.80514	4.30484	1.64685
H	3.30724	-3.22577	1.84391
H	4.65458	-2.28393	2.60038
H	4.96094	-3.93296	1.95818

Mesylate dimer

96

Energy: -3266400.4005276

C	-9.80316	1.51334	1.31957
C	-8.47678	1.31530	0.96984
C	-8.09747	0.16442	0.25470
C	-9.11577	-0.77609	-0.05810
C	-10.44477	-0.58359	0.27147
C	-10.80886	0.58825	0.96860
N	-8.51965	-1.87900	-0.72529
C	-7.11899	-1.71910	-0.76601
C	-6.81425	-0.37109	-0.17024
C	-4.22329	-0.44582	-0.32275
C	-3.23832	0.57879	-0.32516
C	-1.88293	0.31870	-0.41172
C	-1.45200	-1.02395	-0.47705
C	-2.41642	-2.05338	-0.43350
C	-3.77270	-1.77783	-0.36286
C	-5.53523	0.16222	-0.16368
C	-5.28259	1.64106	-0.06006
N	-3.89425	1.83327	-0.22283
O	-6.37125	-2.52867	-1.28735
S	-9.25222	-3.41578	-1.10283
O	-6.07335	2.56267	0.04762
S	-3.16873	3.38887	0.09051
H	-10.06507	2.39431	1.89550
H	-7.73450	2.04843	1.24583
H	-11.18739	-1.31646	-0.01132
H	-1.17378	1.13433	-0.43767
H	-2.09536	-3.08932	-0.43643
H	-4.48380	-2.58930	-0.34296
O	-1.75280	3.23924	-0.23653
C	-8.77386	-3.62444	-2.82274
O	-10.69524	-3.19535	-1.03811
O	-8.61348	-4.44218	-0.28998
O	-3.59035	3.83441	1.41179
C	-12.20141	0.84309	1.33009
S	-13.39476	-0.44471	1.38265
C	-14.64462	0.65511	1.85270
C	-14.17724	1.93775	1.96634
C	-12.79149	2.04415	1.67100
H	-15.64878	0.28995	2.02215
H	-14.80114	2.77957	2.24590
H	-12.25103	2.98392	1.68257
C	-3.95296	4.40486	-1.16745
H	-9.22280	-2.81902	-3.40606
H	-9.17729	-4.59289	-3.12891
H	-7.68554	-3.61357	-2.87672
H	-5.03096	4.36459	-1.01282
H	-3.66317	4.02459	-2.14822
H	-3.56730	5.41635	-1.01736
C	4.88796	-1.95658	-0.34374
C	6.23320	-1.64401	-0.22531
C	6.65134	-0.30114	-0.20770
C	5.64739	0.69619	-0.34180
C	4.30212	0.39963	-0.46255
C	3.90221	-0.95393	-0.45682
N	6.27438	1.96842	-0.35532
C	7.67416	1.82040	-0.31073
C	7.95799	0.34349	-0.18285
C	10.55749	0.44412	-0.24308
C	11.55015	-0.47262	0.19282
C	12.90901	-0.22850	0.04998
C	13.29345	0.96884	-0.56216
C	12.34249	1.87551	-1.03427
C	10.98132	1.62138	-0.88267
C	9.23954	-0.14813	-0.01057

C	9.50904	-1.50304	0.58741
N	10.90628	-1.63498	0.69858
O	8.44122	2.76614	-0.28929
S	5.55321	3.51558	-0.72293
O	8.72714	-2.35906	0.96706
S	11.60886	-2.87043	1.71076
H	4.59020	-2.99924	-0.31672
H	6.95954	-2.43390	-0.11678
H	3.57861	1.19308	-0.58739
H	13.64193	-0.94153	0.39913
H	12.66050	2.79195	-1.52209
H	10.25517	2.33424	-1.24068
O	11.01946	-2.77010	3.03898
C	6.10878	4.50717	0.66902
O	4.11025	3.31551	-0.61697
O	6.15336	4.01508	-1.95244
O	13.05370	-2.76416	1.52086
C	2.49443	-1.31861	-0.57209
S	1.21446	-0.18265	-0.20063
C	-0.03403	-1.35117	-0.58100
C	0.54232	-2.55025	-0.96394
C	1.95295	-2.53252	-0.95752
H	-0.03748	-3.41174	-1.27469
H	2.55811	-3.37846	-1.26307
C	11.02643	-4.36796	0.90546
H	14.35231	1.18337	-0.67648
H	5.68954	4.08403	1.58321
H	5.71664	5.51233	0.49498
H	7.19856	4.50327	0.67599
H	11.43498	-4.39862	-0.10582
H	9.93704	-4.35381	0.90856
H	11.41444	-5.19672	1.50296

Oxirane monomer

53

Energy: -1136628.8554916

C	2.11014	-2.14407	0.04992
C	0.81098	-1.64852	-0.01257
C	0.58443	-0.27979	-0.22744
C	1.73003	0.55101	-0.35025
C	3.02654	0.07011	-0.30184
C	3.23470	-1.31095	-0.10512
N	1.33431	1.87810	-0.51494
C	-0.04380	1.99092	-0.44862
C	-3.13839	1.17274	-0.16119
C	-4.28321	0.33790	-0.26042
C	-5.58083	0.82004	-0.16056
C	-5.75506	2.19128	0.05649
C	-4.65128	3.03571	0.18065
C	-3.34912	2.53934	0.07568
C	-1.95165	0.30826	-0.26694
C	-2.50860	-1.09720	-0.42150
N	-3.88726	-0.98728	-0.44665
O	-0.62372	3.06278	-0.55806
C	2.19915	3.03262	-0.69776
O	-1.92859	-2.16752	-0.55600
C	-4.75496	-2.14152	-0.61314
C	-5.17320	-2.75890	0.70923
H	2.25591	-3.20128	0.24529
H	-0.02574	-2.31983	0.09827
H	3.87122	0.73912	-0.43038
H	-6.43635	0.15683	-0.23953
H	-6.76123	2.59401	0.13460
H	-4.79956	4.09680	0.35775
H	-2.50548	3.20574	0.15932
H	-4.20721	-2.88476	-1.19727
C	2.66230	3.63563	0.61628
H	1.63313	3.78252	-1.25527
H	3.05980	2.73399	-1.30608
H	-5.63617	-1.83765	-1.18859
H	-5.72921	-2.10489	1.38440
C	4.58448	-1.87401	-0.05754
S	5.98000	-0.89324	0.36287

C	7.05533	-2.23904	0.18094
C	6.38319	-3.37659	-0.17605
C	4.98196	-3.16983	-0.31055
H	8.11551	-2.11068	0.35328
H	6.87077	-4.33048	-0.34529
H	4.28991	-3.94688	-0.61522
C	1.93075	4.75226	1.22798
O	3.16408	4.97118	0.52276
C	-4.42367	-3.88373	1.28263
O	-5.68169	-4.09231	0.61891
H	3.24354	2.97579	1.26391
H	1.98075	4.90441	2.30593
H	1.03472	5.12902	0.73686
H	-4.43604	-4.04776	2.35994
H	-3.54632	-4.25666	0.75620
C	-0.60053	0.58395	-0.28725

Oxirane dimer

104

Energy: -2272512.0239863

C	9.81844	1.78377	0.35117
C	8.46015	1.49097	0.42582
C	8.01806	0.15915	0.36926
C	9.01302	-0.84330	0.21279
C	10.36713	-0.56689	0.14899
C	10.79379	0.77536	0.22649
N	8.40889	-2.09754	0.13318
C	7.03006	-1.98009	0.17130
C	6.71158	-0.50531	0.35581
C	4.11129	-0.65821	0.34122
C	3.12418	0.30063	0.69746
C	1.76471	0.05498	0.62336
C	1.32302	-1.20331	0.16218
C	2.28837	-2.15481	-0.22201
C	3.65291	-1.89684	-0.13544
C	5.42451	-0.02887	0.51277
C	5.11846	1.37833	0.99542
N	3.74184	1.47966	1.11097
O	6.28325	-2.94795	0.10652
C	9.07489	-3.38207	-0.01079
O	5.87402	2.29361	1.29500
C	3.09061	2.69111	1.58289
C	2.71566	3.63717	0.45640
H	10.12918	2.82311	0.36854
H	7.74387	2.29001	0.53348
H	11.09287	-1.36862	0.05867
H	1.04836	0.80921	0.93258
H	1.96416	-3.11099	-0.61902
H	4.36216	-2.65448	-0.42874
H	3.78589	3.19691	2.25693
C	9.35682	-3.74022	-1.45892
H	8.42139	-4.14175	0.42436
H	10.00548	-3.36219	0.56680
H	2.20195	2.41066	2.15880
H	2.01790	3.24202	-0.28504
C	12.21405	1.12252	0.17528
S	13.40023	0.05399	-0.55796
C	14.69440	1.14878	-0.20178
C	14.24327	2.27178	0.43758
C	12.83676	2.25732	0.65075
H	15.70701	0.90035	-0.48999
H	14.89260	3.08097	0.75357
H	12.30275	3.04700	1.16727
C	8.41237	-4.56326	-2.22497
O	9.62550	-5.12433	-1.69605
C	3.61782	4.72585	0.05994
O	2.45370	4.98236	0.86316
H	10.00740	-3.05118	-2.00167
H	8.37762	-4.47604	-3.31069
H	7.49244	-4.89561	-1.74611
H	3.57526	5.11931	-0.95541
H	4.57413	4.83154	0.57016
C	-5.05333	-1.91304	0.24751

C	-6.39845	-1.56805	0.15889
C	-6.77617	-0.30966	-0.33574
C	-5.73013	0.57722	-0.70793
C	-4.38927	0.24494	-0.63219
C	-4.02916	-1.03226	-0.15240
N	-6.27020	1.78856	-1.13729
C	-7.64961	1.78113	-1.01621
C	-8.04707	0.40259	-0.51081
C	-10.62458	0.76554	-0.34948
C	-11.67792	-0.17225	-0.17997
C	-13.01307	0.19777	-0.09946
C	-13.32109	1.56015	-0.18219
C	-12.30847	2.50912	-0.32745
C	-10.96755	2.12469	-0.40987
C	-9.35951	0.01384	-0.34106
C	-9.77129	-1.43569	-0.14191
N	-11.15311	-1.46250	-0.09331
O	-8.34391	2.73862	-1.32916
C	-5.54179	2.94577	-1.63232
O	-9.08762	-2.44986	-0.07787
C	-11.90087	-2.69975	0.05962
C	-12.18935	-3.03727	1.51138
H	-4.79103	-2.88217	0.65854
H	-7.15484	-2.27362	0.46411
H	-3.62613	0.94643	-0.95332
H	-13.79626	-0.54209	0.03173
H	-14.35944	1.87522	-0.12566
H	-12.56004	3.56408	-0.38201
H	-10.19646	2.86773	-0.53702
H	-11.30560	-3.50212	-0.38256
C	-5.09902	3.88339	-0.52352
H	-6.20495	3.48539	-2.31249
H	-4.67662	2.59672	-2.20647
H	-12.83513	-2.61690	-0.50631
H	-12.77789	-2.30076	2.06270
C	-2.62961	-1.44166	-0.06419
S	-1.32551	-0.27034	-0.00260
C	-0.10003	-1.52258	0.07798
C	-0.70012	-2.76649	0.05541
C	-2.11140	-2.72130	-0.02240
H	-0.13774	-3.69135	0.11569
H	-2.73254	-3.60864	-0.06848
C	-5.92263	5.03857	-0.14457
O	-4.74640	5.19980	-0.95487
C	-11.30065	-3.93060	2.26513
O	-12.56003	-4.39699	1.75238
H	-4.42758	3.45414	0.22325
H	-5.84944	5.44607	0.86347
H	-6.87110	5.20126	-0.65443
H	-11.24500	-3.84736	3.35031
H	-10.41518	-4.33188	1.77426

Tosyl monomer

69

Energy: -1923548.0407293

C	1.06343	3.10720	-1.30792
C	0.22994	2.00331	-1.20742
C	0.61574	0.89108	-0.43918
C	1.89037	0.93832	0.18665
C	2.72051	2.04329	0.11417
C	2.30365	3.15970	-0.64037
N	2.12194	-0.30465	0.83008
C	1.05161	-1.18680	0.58979
C	0.02584	-0.42320	-0.21276
C	-1.72584	-2.31856	-0.55889
C	-3.10247	-2.27931	-0.90937
C	-3.85819	-3.43031	-1.09100
C	-3.22169	-4.66317	-0.91641
C	-1.86579	-4.73568	-0.59313
C	-1.11469	-3.57538	-0.41848
C	-1.21025	-0.94911	-0.53672
C	-2.38592	-0.07277	-0.88994
N	-3.48971	-0.92454	-1.07591

O	1.01704	-2.32459	1.02010
S	3.47252	-0.78637	1.82579
O	-2.46323	1.14195	-0.93102
S	-5.07177	-0.27663	-1.42393
H	0.75413	3.94107	-1.92897
H	-0.72071	2.00297	-1.71769
H	3.66140	2.05216	0.64572
H	-4.90055	-3.37173	-1.36841
H	-1.38630	-5.70281	-0.47435
H	-0.06974	-3.63784	-0.15856
O	-5.94779	-1.44474	-1.49626
C	4.20436	-2.13783	0.91810
O	2.92985	-1.27683	3.08390
O	4.39145	0.34978	1.78386
O	-4.92471	0.63266	-2.55104
C	3.14181	4.35406	-0.73788
S	4.86444	4.30570	-0.39820
C	5.03649	5.99571	-0.72976
C	3.84305	6.56713	-1.08233
C	2.76892	5.63583	-1.08807
H	6.00704	6.46623	-0.64625
H	3.73112	7.61859	-1.32343
H	1.74476	5.90916	-1.31573
C	-5.48149	0.64548	0.04810
H	-3.79870	-5.57443	-1.04782
C	3.84841	-3.45207	1.22507
C	4.45953	-4.49034	0.52581
C	5.41550	-4.23673	-0.46806
C	5.75158	-2.90412	-0.75076
C	5.15766	-1.85033	-0.06219
C	-5.19637	2.01041	0.11448
C	-5.55558	2.71276	1.26234
C	-6.18789	2.07559	2.33946
C	-6.46920	0.70498	2.23616
C	-6.12290	-0.01728	1.09781
C	6.09182	-5.37246	-1.19718
C	-6.53988	2.84236	3.59118
H	3.10478	-3.64665	1.98791
H	4.18796	-5.51715	0.75715
H	6.49278	-2.68896	-1.51624
H	5.43456	-0.82240	-0.27005
H	-4.70241	2.50095	-0.71480
H	-5.34116	3.77672	1.32128
H	-6.97309	0.19792	3.05503
H	-6.36067	-1.07209	1.01295
H	7.01789	-5.66814	-0.68656
H	6.36091	-5.08893	-2.21983
H	5.44933	-6.25735	-1.24507
H	-6.74256	3.89566	3.37260
H	-5.71268	2.81204	4.31267
H	-7.41983	2.41986	4.08682

Tosylate dimer

136

Energy: -3846344.7744932

C	-10.03800	-1.99855	-1.03394
C	-8.69540	-1.73080	-0.80616
C	-8.31415	-0.77935	0.15582
C	-9.35472	-0.09302	0.84156
C	-10.69519	-0.34905	0.62181
C	-11.05542	-1.33254	-0.32432
N	-8.76809	0.87825	1.69203
C	-7.36758	0.86779	1.55243
C	-7.03107	-0.22159	0.56522
C	-4.45721	0.13089	0.44810
C	-3.39826	-0.71520	0.01763
C	-2.07271	-0.31992	0.02202
C	-1.75024	0.98276	0.45824
C	-2.79210	1.84264	0.86193
C	-4.11675	1.43257	0.85716
C	-5.72470	-0.56315	0.25488
C	-5.35994	-1.89929	-0.33904
N	-3.95508	-1.93931	-0.43009

O	-6.63186	1.60660	2.18114
S	-9.54120	1.89707	2.87992
O	-6.07272	-2.83131	-0.66392
S	-3.13296	-3.38854	-0.94931
H	-10.30060	-2.76352	-1.75849
H	-7.94065	-2.27570	-1.35077
H	-11.45087	0.20963	1.15229
H	-1.30398	-0.99767	-0.32080
H	-2.55702	2.84008	1.21756
H	-4.88719	2.10672	1.19655
O	-3.61596	-4.48555	-0.12409
C	-9.14602	3.55020	2.33439
O	-8.90289	1.63783	4.16183
O	-10.97391	1.67675	2.68583
O	-1.71700	-3.02459	-0.97834
C	-12.46958	-1.63144	-0.54258
S	-13.03215	-2.41743	-2.00997
C	-14.67574	-2.38612	-1.46579
C	-14.78904	-1.80381	-0.23183
C	-13.53856	-1.37437	0.29124
H	-15.45902	-2.79591	-2.08935
H	-15.73211	-1.69007	0.29146
H	-13.42618	-0.91443	1.26667
C	-3.69200	-3.60983	-2.63034
C	-8.04712	4.21508	2.88078
C	-7.78709	5.52175	2.47439
C	-8.60094	6.16846	1.53326
C	-9.70263	5.47470	1.01039
C	-9.98518	4.17006	1.40519
C	-4.77833	-4.44553	-2.89496
C	-5.17098	-4.63202	-4.21824
C	-4.49791	-4.00138	-5.27451
C	-3.40897	-3.16951	-4.97396
C	-2.99544	-2.97155	-3.65950
C	-8.28884	7.57382	1.07884
C	-4.91593	-4.23698	-6.70587
H	-7.41695	3.71355	3.60478
H	-6.93776	6.05002	2.90002
H	-10.35401	5.96615	0.29220
H	-10.85155	3.64577	1.01660
H	-5.30202	-4.92626	-2.07805
H	-6.01663	-5.28012	-4.43381
H	-2.87345	-2.67512	-5.78049
H	-2.14042	-2.34375	-3.43290
H	-9.19744	8.11574	0.79735
H	-7.62989	7.56347	0.20046
H	-7.77883	8.14427	1.86158
H	-5.98850	-4.44236	-6.78162
H	-4.38768	-5.10112	-7.13011
H	-4.68722	-3.37313	-7.33826
C	4.10785	0.06631	0.84027
C	5.47290	-0.18152	0.87008
C	6.38725	0.84120	0.56533
C	5.85227	2.10551	0.19407
C	4.49543	2.36667	0.16313
C	3.59482	1.33453	0.50590
N	6.92558	2.95996	-0.16564
C	8.15044	2.27180	-0.07666
C	7.83908	0.88172	0.42382
C	10.23958	-0.12530	0.34726
C	10.81054	-1.25767	0.98922
C	12.13329	-1.63474	0.79747
C	12.90901	-0.87091	-0.08015
C	12.36853	0.22636	-0.75252
C	11.04364	0.60335	-0.54492
C	8.82545	-0.04245	0.71515
C	8.58226	-1.21699	1.63035
N	9.80897	-1.88781	1.77173
O	9.22198	2.78876	-0.33204
S	6.88071	4.65803	-0.56633
O	7.56601	-1.55596	2.20962
S	9.97131	-3.23493	2.87158

H	3.42793	-0.73544	1.11197
H	5.83313	-1.15726	1.15509
H	4.13526	3.33799	-0.13898
H	12.54689	-2.48946	1.31248
H	12.98423	0.80136	-1.43782
H	10.63834	1.46438	-1.05232
O	9.50121	-2.79269	4.17608
C	7.54160	4.71642	-2.22353
O	7.78126	5.34770	0.34529
O	5.45759	4.99080	-0.62531
O	11.33954	-3.71168	2.67618
C	2.16087	1.60668	0.50291
S	0.97833	0.31497	0.44498
C	-0.36448	1.44042	0.48663
C	0.11135	2.73817	0.54317
C	1.52029	2.83169	0.54936
H	-0.53899	3.60527	0.56220
H	2.05292	3.77416	0.60828
C	8.84889	-4.45522	2.21008
H	13.94814	-1.14687	-0.23719
C	8.89789	4.98212	-2.41896
C	9.38207	5.06098	-3.72257
C	8.53672	4.88062	-4.82671
C	7.17846	4.61637	-4.59512
C	6.67086	4.53770	-3.30133
C	7.55121	-4.54976	2.71630
C	6.70723	-5.53584	2.21162
C	7.13791	-6.42472	1.21585
C	8.44859	-6.30395	0.73079
C	9.31183	-5.32891	1.22338
C	9.06963	4.99902	-6.23418
C	6.22028	-7.50694	0.70044
H	9.55140	5.11378	-1.56555
H	10.43711	5.26654	-3.88449
H	6.50799	4.47666	-5.43929
H	5.61637	4.35291	-3.12695
H	7.21720	-3.85866	3.48018
H	5.69468	-5.61599	2.59884
H	8.80042	-6.98687	-0.03827
H	10.33103	-5.25201	0.86026
H	8.99848	6.03432	-6.59311
H	8.50366	4.37320	-6.93169
H	10.12320	4.70717	-6.28978
H	5.17559	-7.17950	0.69931
H	6.27953	-8.40291	1.33241
H	6.48608	-7.80763	-0.31793
